# Identification of plant promoter constituents by analysis of local distribution of short sequences

**DOI:** 10.1186/1471-2164-8-67

**Published:** 2007-03-08

**Authors:** Yoshiharu Y Yamamoto, Hiroyuki Ichida, Minami Matsui, Junichi Obokata, Tetsuya Sakurai, Masakazu Satou, Motoaki Seki, Kazuo Shinozaki, Tomoko Abe

**Affiliations:** 1 Application and Development Group, RIKEN FRS, Hirosawa 2-1, Wako, Saitama 351-0198, Japan; 2 Center for Gene Research, Nagoya University, Furo-cho, Chikusa-ku, Nagoya, Aichi 464-8602, Japan; 3 Graduate School of Science and Technology, Chiba University, Matsudo 648, Matsudo, Chiba 271-8510, Japan; 4 RIKEN Genomic Sciences Center, Suehirocho 1-7-22, Tsurumiku, Yokohama, Kanagawa 230-0045, Japan; 5 RIKEN Plant Science Center, Suehirocho 1-7-22, Tsurumiku, Yokohama, Kanagawa 230-0045, Japan

## Abstract

**Background:**

Plant promoter architecture is important for understanding regulation and evolution of the promoters, but our current knowledge about plant promoter structure, especially with respect to the core promoter, is insufficient. Several promoter elements including TATA box, and several types of transcriptional regulatory elements have been found to show local distribution within promoters, and this feature has been successfully utilized for extraction of promoter constituents from human genome.

**Results:**

LDSS (Local Distribution of Short Sequences) profiles of short sequences along the plant promoter have been analyzed *in silico*, and hundreds of hexamer and octamer sequences have been identified as having localized distributions within promoters of *Arabidopsis thaliana *and rice. Based on their localization patterns, the identified sequences could be classified into three groups, pyrimidine patch (Y Patch), TATA box, and REG (Regulatory Element Group). Sequences of the TATA box group are consistent with the ones reported in previous studies. The REG group includes more than 200 sequences, and half of them correspond to known *cis*-elements. The other REG subgroups, together with about a hundred uncategorized sequences, are suggested to be novel *cis*-regulatory elements. Comparison of LDSS-positive sequences between *Arabidopsis *and rice has revealed moderate conservation of elements and common promoter architecture. In addition, a dimer motif named the YR Rule (C/T A/G) has been identified at the transcription start site (-1/+1). This rule also fits both *Arabidopsis *and rice promoters.

**Conclusion:**

LDSS was successfully applied to plant genomes and hundreds of putative promoter elements have been extracted as LDSS-positive octamers. Identified promoter architecture of monocot and dicot are well conserved, but there are moderate variations in the utilized sequences.

## Background

The determination of complete genome sequences has allowed analysis by various statistical methods that have furthered understanding of the function of genomes. Analysis of promoter structure is one of the most important issues. Understanding of promoter structure allows predictions concerning promoter positions and expression profiles, and sheds light on hidden transcriptional networks.

Several functional elements have been identified as promoter constituents for precise and regulated transcriptional initiation: TATA box, Initiator (Inr) motif, Downstream Promoter Element (DPE, found from *drosophila*), TFIIB-Recognition Element (BRE), and so-called *cis*-regulatory elements [1-3]. In addition, some mammalian promoters are associated with CpG islands [4,5], which is related to the Sp1 recognition site [6] and have some relationship with gene regulation by DNA-methylation [3,7]. Human transcriptional regulatory elements are reported to make clusters (modules) at the promoter region as well as the 3' end of a gene [8]. Transcription start sites (TSS) in plant promoters have a CG-compositional strand bias, or GC-skew, where C is more frequently observed in the (+) strand than G [9,10]. Some of these features are well understood and some are not, but all these features are useful to understand individual promoters. Some of the above features have been utilized for promoter prediction [11-13]. Although these studies obtain certain success, our current knowledge of promoters is still insufficient [13].

Availability of microarray data on co-regulated gene expression on a genomic scale has enabled the prediction of novel *cis*-elements involved in gene regulation. Several approaches have been developed for this detection of consensus sequences in a co-regulated promoter set (Gibbs Motif Sampling [14,15], MEME [16]), and detection of over-represented sequence in co-regulated promoters with a set of reference sequences [17,18]. These approaches are also applicable to chromatin immunoprecipitation (ChIP) data [19,20]. In addition, identification of conserved promoter sequences by comparative genomics supports the prediction of regulatory elements [21-24].

Studies on plant transcription factors and functional *cis*-regulatory elements have been summarized in several databases, and the collective information of *cis*-elements and/or transfactor-binding DNA sequences are utilized for interpretation of plant promoters (PLACE: [25], AGRIS: [26], AthaMap: [27,28]). Basis of these databases are published articles reporting analyses of individual promoters or transfactors, rather than large scale genomic analyses. Therefore, lack of large scale functional analyses of transcription factors in plant science is reflected in these databases as well.

In contrast to the above fact-based approaches, *in silico *prediction of plant promoter elements by survey of the *Arabidopsis *genome is also reported. Molina and Grotewold applied the MEME and Gibbs sampling methods to *Arabidopsis *core promoter regions with genomic scale, and detected several motifs including a plant TATA motif and microsatellites [29].

Recent studies on mammalian promoter elements have revealed that some of them have localized appearance along the promoter region, exemplified by the TATA box [30], and binding sites for NRF-1, Sp1, CREB, ATF, and E2F [31]. These studies evoke the idea that localized distribution is a signature of a functional element of the promoter. Recently, this feature was successfully utilized for extraction of functional sequences from human promoters [32]. Large-scale deletion analysis of human promoters suggested that there is some relationship between presence of functional elements and distance from TSS [33].

In this report, we have detected hundreds of short sequences showing localized distribution in plant promoters by comprehensive analyses of short sequences. The extracted sequences are mentioned as "LDSS (Local Distribution of Short Sequence)-positive" in this work. These sequences includes TATA boxes, various regulatory sequences identified in previous studies, a novel sequence group that would be a general component of a core promoter, and also many novel sequences that share many characteristics with regulatory sequences. Our analyses have also revealed conservation of the promoter architecture between monocot and dicot plants.

## Results

### Patterns of distribution of peaks

Typically, DNA elements recognized by a protein (complex) is within the range of 5 to 15 bp long [34]. Within this range, we decided to analyze localization patterns of hexamer and octamer sequences. Our results suggest that sequences longer than 9 bps would not provide enough number of appearance to survive statistical analysis.

**Figure 1:**
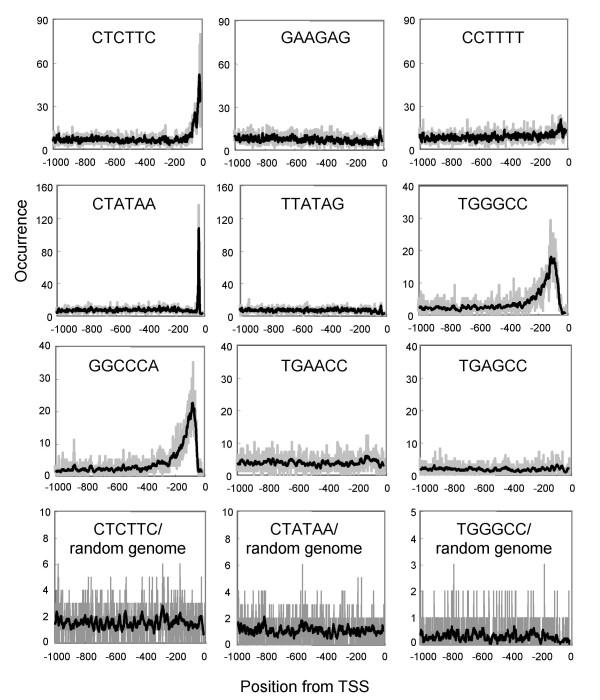
**Examples of distribution of peaks**. Several examples of hexamer analysis against *Arabidopsis *promoters are shown. The vertical axis indicates the total count of the whole promoter database. Gray and solid lines show raw and average with 15 bin (width of window), respectively. Instead of the promoter database, a set of 3,000 random fragments of 1 kb length from the *Arabidopsis *genome were used for the occurrence analysis as negative controls (shown as "random genome" in the bottom columns).

For each hexamer sequence, a distribution profile in relation to distance from the TSS was analyzed for *Arabidopsis thaliana*. Looking through all the distribution profiles, we noticed that there are quite a few patterns. Most sequences have a flat distribution profile with no special tendency (Fig. 1, GAAGAG). Sometimes the base line has a slight slope with a higher frequency toward the TSS. There are also groups with peaks, and they can be classified according to the peak position. We refer these sequences as LDSS-positive.

One example of a LDSS-positive sequence, (Fig. 1, CTCTTC) has a peak of appearance at the TSS. Its complementary sequence (Fig. 1, GAAGAG) has a distinct distribution profile, showing that its appearance is sensitive to the direction of transcription. Although hexamers with this type of distribution profile tend to have only C and T in the sequence (see later), there seems to be weak sequence preference, and not all the sequences filled with C and T show a peak-positive distribution (Fig. 1, CCTTTT is a peak-negative example).

A second example (Fig. 1, CTATAA) is a TATA box-related sequence. This has a peak around -35 bp, and the peak is very sharp. The complementary sequence showed a different pattern with no peak (Fig. 1, TTATAG).

A third example (Fig. 1, TGGGCC) has a relatively wide and low peak. Complementary sequence of this sequence shows the same peak (Fig. 1, GGCCCA). Peak position and direction-insensitivity suggest that sequences with this type of distribution profile are so-called *cis*-regulatory sequences involved in transcriptional regulation [34]. In fact, TGGGCC in Figure 1 is reported to be necessary for meristematic expression in *Arabidopsis*, and mutation to TGAACC abolished the expression (Element II of *Arabidopsis *PCNA-2, [35]). Interestingly, distribution of the mutated sequence does not have any peaks (Fig. 1, TGAACC), demonstrating a good correlation between functionality and peak distribution. In addition, one base substitution, TGAGCC, also caused the loss of the peak (Fig. 1). It is common that one base substitution drastically changes the distribution profile (data not shown).

As controls, a set of random genomic sequences of 1 kb length was used for the distribution analysis instead of the promoter database. When sequences with distribution patterns of peak-positive sequences were applied to this analysis, they were found to have no peaks in the random genome fragments (Fig. 1, CTCTTC/random genome, CTATAA/random genome, TGGGCC/random genome).

Beside LDSS-positive elements, there are many LDSS-negative sequences. Among them, frequently observed sequences beyond the theoretical occurrence rate (0.24 per a 1 kb region) are rich in AT and might promote promoter context, and rare sequences are rich in GC and they might disturb promoter function when located within the promoter region. Therefore, it might be possible to utilize these LDSS-negative sequences as well for evaluation of promoter context.

### Parameters for peak evaluation

**Figure 2:**
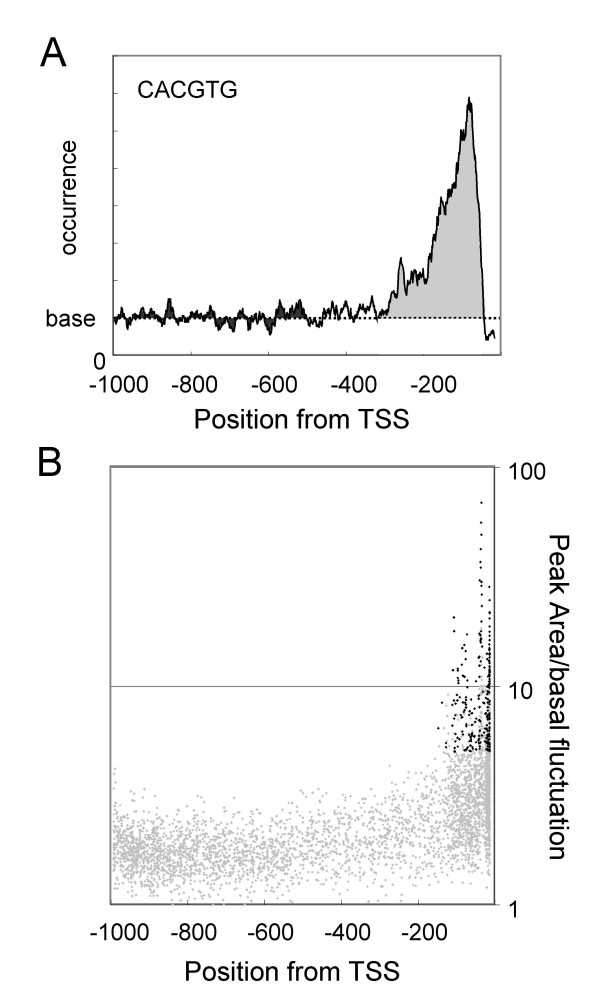
**Parameters for peak detection**. (A) Graph is a distribution profile of CACGTG in *Arabidopsis *promoters. Average with 15 bin is shown. The dotted line indicates the Base Line, which is an average of -1,000 to -500. The light grey area shows Peak Area. The dark grey area is Δarea, an indication of the fluctuation from the Base Line from -1,000 to -500. In addition, the following parameters have been defined: Relative Peak Area (RPA) = Peak Area/total area; Relative Peak Height (RPH) = peak height/Base Line; Peak Area/basal fluctuation = Peak Area/Δarea per peak width; Peak height/SD = peak height/standard deviation of occurrence from -1000 to -500. Several parameters of this graph are shown in Table 2 (CACGTG). (B) All the hexamers were analyzed to obtain various parameters, and (Peak Area/basal fluctuation) and peak position were calculated. The graph shows the results. Each dot shows the data of an individual hexamer. Among the 4,096 hexamers (grey dots), 247 peak positive hexamers have been selected (solid dots). The graph demonstrates that hexamers with a significant value have a peak position from -200 to -13 (the most downstream position after smoothing).

Figure 2A shows a close-up of a typical distribution profile of the regulator type. In order to detect peak-positive sequence, we calculated several parameters. Curve fitting with Gaussian did not give good results (data not shown), because the peak shape is not symmetrical, as seen in the figure. Through analysis of distribution profiles of all the hexamers, we noticed that all of the observed peaks were located downstream of -200 bp. This enabled a base line to be established (Base in the figure) as an average of occurrence between -1,000 and -500 bp. Then we calculated the Relative Peak Height (RPH), and Relative Peak Area (RPA) for evaluation of peak strength. Fluctuation around the base line between -1,000 and -500 bp was also evaluated (see figure legend).

Figure 2B shows the relationship between peak position and a parameter of peak strength. As shown, all the strong peaks locate downstream of -200 bp while weak peaks are scattered throughout the promoters. One important point of the figure is the continuous distribution of hexamers across the vertical axis. The continuous nature was also observed when RPH or RPA was represented in the graph on the vertical axis (data not shown). These results mean that there is no clear way to separate peaky and flat groups. In this study, we took a strategy to list sequences with strong peaks, leaving out a flat group and a group with ambiguous peaks.

Considering peak height, peak area, and fluctuation from the base line, we selected 247 sequences from all the hexamers as peak positive (Fig. 2B, black dots, Table S1 [see Additional file 1]).

### Peak-positive hexamers can be classified according to their peak position

The LDSS-positive hexamers identified were then classified into three major groups as mentioned above. The first group, including CTCTTC, in Figure 1, localize from -100 to -13 bp. They typically have a peak at the most downstream region of the promoter (position -13, Table 1), but peak positions distribute from -13 to -60. Most of their sequences are composed of only C and T, we refer to this group as Y Patch (Y for pyrimidine). As shown in the table, Y Patch sequences are found in the majority of *Arabidopsis *promoters.

**Table 1:** Y Patch and TATA Box identified from *Arabidopsis *hexamer analysis

Sequence	Peak position^1 ^(bp)	Peak width^2 ^(bp)	#promoter^3^	Relative Peak Height (RPH)	Relative Peak Area (RPA)
Y Patch					
TCTCTC	-13	158	6,741	10.96	0.25
CCTCTC	-13	107	3,106	8.13	0.20
CTTCTC	-13	88	5,916	7.64	0.15
CTCCTC	-13	81	3,180	7.23	0.12
CTCTTC	-13	91	5,393	7.02	0.14
CTCTCC	-13	108	3,153	6.95	0.16
TCCCTC	-13	93	2,140	6.13	0.15
TTCTTC	-13	75	8,829	5.78	0.11
TTCTCT	-13	109	8,314	5.77	0.12
TATA Box					
TATAAA	-35	30	10,704	9.0	0.10
TATATA	-36	27	10,315	6.38	0.07
ATATAA	-35	27	10,062	6.14	0.07
ATAAAT	-35	27	10,572	5.14	0.05
TAAATA	-34	25	9,801	4.65	0.04
ATATAT	-35	24	10,412	3.84	0.04
TTATAA	-36	23	9,172	3.36	0.03
TTATAT	-36	23	9,639	3.10	0.03

^1^In this analysis, -13 is the position for an average from -20 to -6 that covers a region from -20 to -1, so -13 is the most downstream position.

^2^Peak width at the bottom of the peak.

^3^Number of promoters containing the element out of 15,607 *Arabidopsis *promoters (-1,000 to -1). Number of promoters containing an element within the peak area can be roughly estimated by #promoters × RPA. For example, TATAAA is found in approx. 1,070 promoters within the peak area (10,704 × 0.10).

The second group contains TATA box-related sequences. An example is shown as CTATAA in Figure 1. The characteristics of this group are high peak height, narrow peak width, and stringent peak position (Table 1, TATA Box). Similar to Y Patch, the TATA box group sequences are also found in the majority of *Arabidopsis *promoters, although promoters with the TATA Box within the peak are is about 1,000 or less for each sequence.

The third group, including TGGGCC in Figure 1, is referred to as REG, for Regulatory Element Group, in this study. The peak positions of this group locate around -80 bp, and they have a wide peak width in comparison with that of the TATA box group (Table 2). Another feature of the group is high coverage of Peak Area against total area. This means high specificity of localization within a promoter. As shown in Relative Peak Area (RPA) of the table, around 50% to 30% of a REG sequence is found in the peak area. These ratios are much higher than those of the Y Patch (25 to 10%) or TATA box (11 to 5%) groups. Compared to these, the number of promoters containing a REG sequence is smaller, consistent with the idea that each REG is not a component of the general core promoter but a specific regulator of gene expression. In fact, Table 2 contains several known *cis*-regulatory elements, including Element II of *Arabidopsis *PCNA-2 (GGCCCA, TGGGCC, and AGCCCA) [35] and G-box/ABRE (CACGTG, CGTGGC, CCACGT, and GCCACG) [36].

**Table 2:** REGs identified from *Arabidopsis *hexamer analysis

Sequence	Peak position (bp)	Peak width (bp)	#promoter	Relative Peak Height (RPH)	Relative Peak Area (RPA)
AGGCCC	-76	326	2,005	14.78	0.54
GGCCCA	-73	347	1,225	12.26	0.53
GGGCCT	-106	240	1,764	10.31	0.47
TGGGCC	-107	262	2,867	9.29	0.46
GGGCCC	-91	256	711	9.51	0.44
GCCCAT	-76	320	2,925	8.41	0.43
GCCCAA	-72	366	3,068	7.78	0.42
AGCCCA	-85	284	2,963	7.53	0.39
CACGTG	-80	273	3,039	6.85	0.38
AAGCCC	-86	299	2,593	7.48	0.37
CGGCCC	-62	189	732	7.66	0.36
CCACGT	-83	260	2,367	5.66	0.35
ATGGGC	-97	295	2,836	6.29	0.35
CGTGGC	-97	251	1,459	5.96	0.35
TAGGCC	-75	311	1,435	6.18	0.34
CGTGTC	-79	289	1,909	5.57	0.33
AAGGCC	-77	287	1,935	6.27	0.33
GCGCGT	-59	244	632	5.56	0.32
GCCACG	-83	215	1,411	6.64	0.31
ACGCGC	-65	190	655	5.08	0.31
GGGCCG	-85	196	711	6.01	0.30
CACGCG	-138	182	884	5.22	0.30

In addition to these three groups, there is also small number of exceptional hexamers with peak positions in the core promoter (-13 to -60). They might constitute a minor type(s) within the core promoter (Table S1, "others" [see Additional file 1]. See also Table S2 and S3 for these elements). The complete list of the extracted sequences is shown in Table S1. The table shows 103 Y Patch, 39 TATA-related, 38 REG, and 22 unclassified hexamer sequences.

### Directional preference relative to transcription

**Figure 3:**
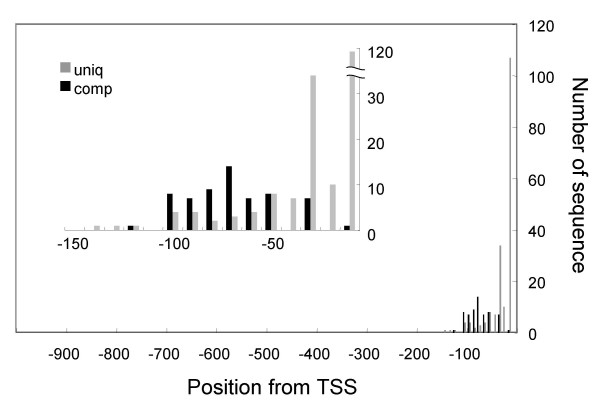
**Directional preference of LDSS-positive hexamers**. When the corresponding complementary sequence was not found in the LDSS-positive group, the hexamer was counted as "uniq", which means orientation-sensitive. When found, the sequence was counted as "comp", meaning direction-insensitive. The number of both hexamers were counted according to the peak position from the TSS, and summarized in a bar graph. The inset graph is an enlargement to show more detail around the TSS.

Subsequently, we examined if the orientation of the hexamers is critical. The identified hexamers were tested to determine if their complementary sequences were also included or not. If the complementary sequence was also found in this positive group, the original sequence is considered as direction-insensitive, and if not, direction-sensitive. As shown in Figure 3, the downstream region from -50, that is known to be the core promoter region [1] and includes the Y Patch and TATA box groups, is occupied with direction-sensitive sequences ("uniq" in the figure), while the upstream region, containing the REG group, is rich in direction-insensitive sequences ("comp" in the figure). These findings are consistent with the established idea that the core promoter determines position and direction of transcription, and *cis*-elements are direction insensitive. These findings further support the idea that the Y Patch and TATA box sequences are core promoter elements and REG sequences are the *cis*-elements [34].

### Comparison of *Arabidopsis *and rice promoters

Subsequently, we analyzed the distribution of octamer sequences. The average of octamer appearance rates is 15.7-fold less than the one of hexamers, consistent with a mathematical expectation of 16-fold difference (data not shown). Because rare sequences tend to show more fluctuations by chance, statistical evaluation was more critical for octamer analysis. We prepared random distribution populations and used them for statistical evaluation of each octamer (Figure S1 [see Additional file 2]). In this study, we have set a p value of 1 × 10^-5 ^as a threshold. In addition, data of the complementary sequences was merged only for REG detection to increase total count of an octamer in the database. Through the octamer analyses, we have identified 350 and 418 LDSS-positive core elements (Table S2 [see Additional file 3] and S3 [see Additional file 4]), and 308 and 242 REG sequences from *Arabidopsis *and rice, respectively (Table S4 [see Additional file 5] and S5 [see Additional file 6]). Sum of the p values for all the extracted octamers of individual species were around 1 × 10^-3 ^each, so false-positive sequences by pure random distribution are not likely to be included in the lists.

**Figure 4:**
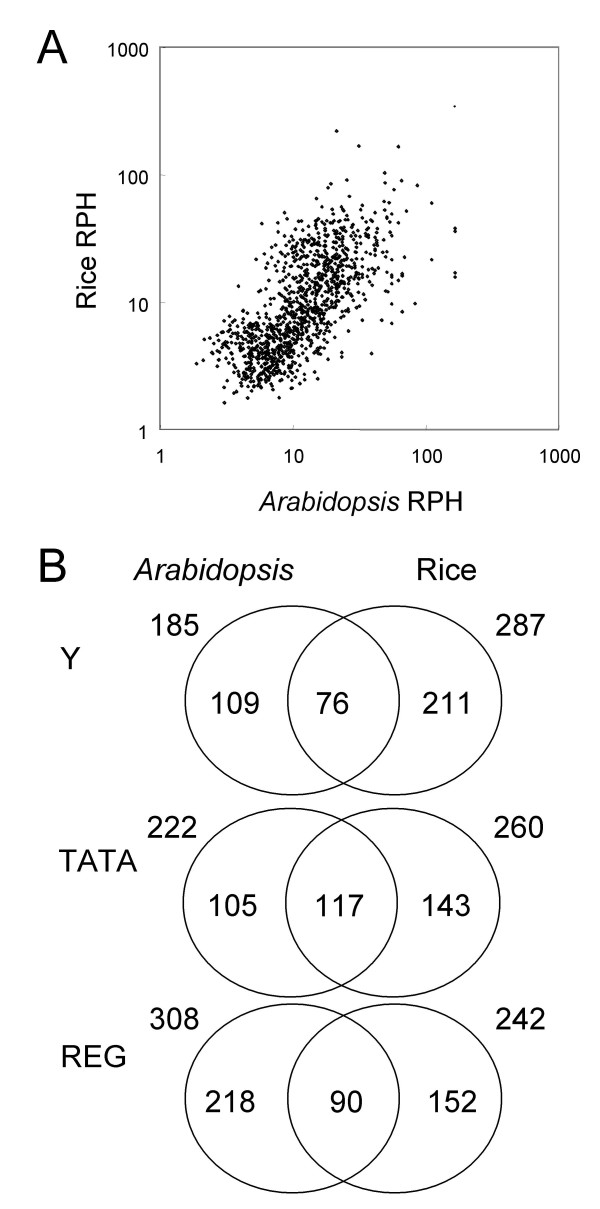
**Comparison of *Arabidopsis *and rice octamers**. (A) 987 octamers that are LDSS-positive in either *Arabidopsis *or rice promoters were selected and their Relative Peak Height (RPH) was compared and expressed as a scatter plot. Each dot is data from an individual octamer sequence. (B) LDSS-positive octamer sequences of *Arabidopsis *and rice were compared, and common sequences found in both sets were identified. The figure shows the number of octamer sequences. Classification into the Y and TATA groups were done based on distribution profiles as shown in Figure 5. The REG group has a peak position between -51 and -200.

For comparison of *Arabidopsis *and rice elements, Relative Peak Height (RPH) values of all the positive octamers in either of the two promoter databases were represented (Fig. 4A). If a sequence has the same RPH value in the *Arabidopsis *and rice databases, a dot appears on the diagonal line. As shown in the figure, we found that RPH values are moderately conserved between *Arabidopsis *and rice (Fig. 4B). The figure also indicates that a considerable number of the sequences have a large difference in the parameter between *Arabidopsis *and rice. Figure 4B shows Venn diagram of the number of positive octamers in *Arabidopsis *and rice. As shown in the figure, approximately 30 to 50% of the octamers are conserved between *Arabidopsis *and rice for both core groups of Y Patch and TATA box, and the REG group. Presence of all the three categories in *Arabidopsis *and rice, and sequential conservation as shown in the figure indicate that promoter architecture of these plant species is essentially conserved. On the other hand, divergence of the positive sequences might reflect differentiation of the corresponding *trans*-factors between these species.

### Classification of *Arabidopsis *LDSS-positive octamers by distribution profiles

**Figure 5:**
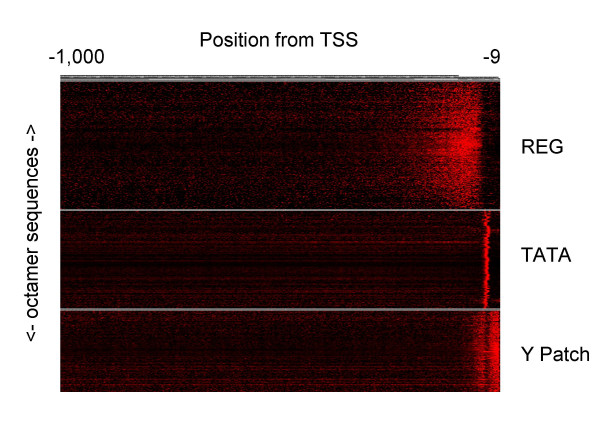
**Clustering of LDSS-positive sequences based on distribution profiles**. Distribution profiles of each LDSS-positive octamer of *Arabidopsis *were subjected to hierarchical clustering. Three major clusters are shown.

All the LDSS-positive sequences from *Arabidopsis *were subjected to clustering analysis according to their distribution profiles. As expected from previous hexamer analyses, major clusters are REGs, TATA box, and Y Patch (Fig. 5). As shown in the figure, distribution profiles within each group (clusters) are quite similar, suggesting functional conservation within each group. The observed clear classification of the LDSS-positive sequences, represented in Figure 5, suggest that the local distribution is a quite useful feature in extraction of putative functional elements in the promoter.

### Clustering of *Arabidopsis *REGs based on presence and absence in promoters

Subsequently, we did classification of 308 *Arabidopsis *REGs with the aid of the promoter database. For each promoter, number of appearance for each REG was scored, and two-dimensional REG-promoter clustering was performed. This REG-promoter association has revealed that 10,334 out of 12,951 *Arabidopsis *promoters have at least one REG at the region of -400 to -40 bp. This high coverage (80%) is due to the long list of REG sequences.

**Figure 6:**
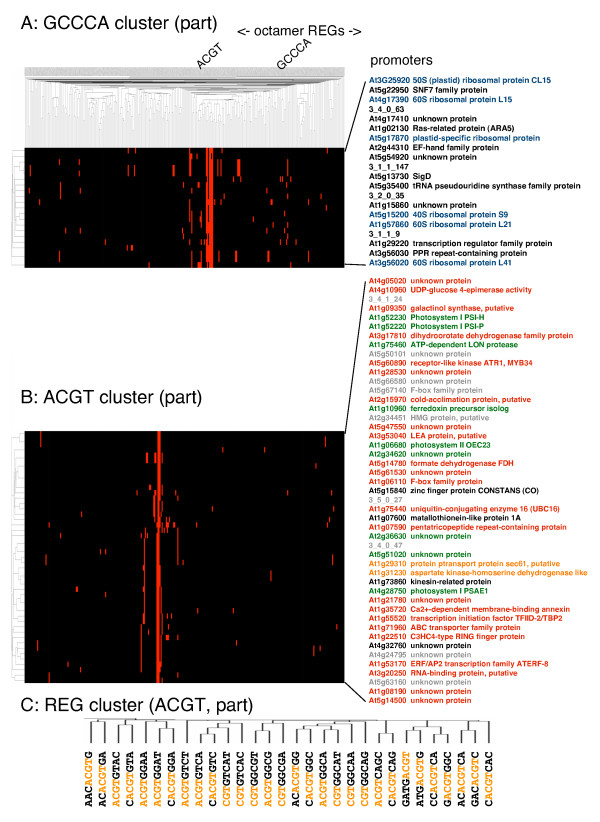
**REG-promoter clustering**. For each *Arabidopsis *promoter, number of each octamer REG within a region from -400 to -40 bp was scored, and subjected to 2D hierarchical clustering. The vertical axis shows promoters and the horizontal axis does REGs. The matrix means number of REG sequences. Two small promoter clusters are shown in the figure together with the whole REGs. (A) A part of promoter cluster rich in GCCCA motif for meristematic expression. Ribosomal proteins are shown in blue. (B) A part of promoter cluster rich in ACGT motif for environmental response. Promoter names are expressed in color according to expression data from AtGenExpress. Red: abiotic stress-positive, orange: abiotic stress-negative, green: light-positive, black: no response to abiotic stress or light, grey: no expression data found. (C) An example of clustered REGs. A part of the ACGT cluster shown in the top of Panel A is enlarged. ACGT in the octamers are highlighted with orange.

This 2D clustering puts co-localized REGs proximal, and promoters with similar REG compositions also come close. Two promoter clusters are shown in Figure 6A and 6B. One cluster of promoters (A) are rich in GCCCA-containing REGs, and another cluster (B) have ACGT-containing REGs. GCCCA-containing REGs is the same kind as TGGGCC (Figure 1) and known to show cell cycle-dependent expression and meristematic expression (Group 1, Table 3). Interestingly, this promoter group is rich in ribosomal proteins. As shown in Figure 6A, as high as 38% (6 out of 16) of the annotated promoters are for ribosomal proteins (Fig. 6A, blue). In contrast, ribosomal promoters are not included in the ACGT-containing promoter clusters (Fig. 6B). Instead, the latter cluster is rich in photosynthesis-related genes and stress-responsive genes, both of which would show environmental responses. In fact, as many as 34 out of 38 genes in this cluster with expression data are responsive to light (Fig. 6B, green) or abiotic stress including salt, drought, and cold (Fig. 6B, red and orange), according to public microarray data [37,38]. Although this clustering is not so accurate as to distinguish between light and stress responses, it has been proved to classify genes with respect to gene expression with a certain range of accuracy. The results are reasonable because *cis*-elements for light response (G-box: CACGTG, [36]) and stress response (ABRE: ACGTGTC, [39]) are related sequences both of which belong to the ACGT motif for environmental responses (Group 2, Table 3). Therefore, clustering of promoters appears reasonable, although the accuracy may not be enough for pinpoint speculation of gene function.

**Table 3:** Classification of octamer REGs

Group	Motif^1^	Motif name	Comment	Trans factor	Expression	Reference	At^1^	Rice^1^	At & Rice^2^
1	GCCCA	Element II of *Arabidopsis *PCNA-2, Site IIa of rice PCNA		PCF1, PCF2, TCP20	cell cycle/meristematic expression	[35, 60]	36	68	71
2	ACGT	"ACGT Core", G-box, ABRE,		bZIP family (GBF, TGA1, etc.), PIF3	environmental response (light, UV, drought, ABA)	[36, 61]	33	4	9
3	ACGCGC	CGCG box		AtSR1(CaMBP)	stress response?	[62]	7	1	0
4	CCGAC	DRE	DRE core	DREB/CBF	stress response	[39]	9	3	0
5	AACCG(G/A)	novel	overlapping with GT1 box (TTAACC)	?	not known	this study	36	1	0
6	AAACG(C/G)	novel		?	not known	this study	13	1	2
7	ACCCCT	novel		?	not known	this study	4	0	0
8	ACCCT	novel		?	not known	this study	4	0	0
9	ACGGGC	novel		?	not known	this study	2	5	1
10	CCATGG	novel		?	not known	this study	1	1	2
11	CCAACGG	novel		?	not known	this study	1	4	6
12	GGGACCC	novel		?	not known	this study	4	3	4
Rest							74	66	1
Total							308	242	90

^1^Number of octamer sequences. This classification is not completely mutually exclusive.

**Figure 7:**
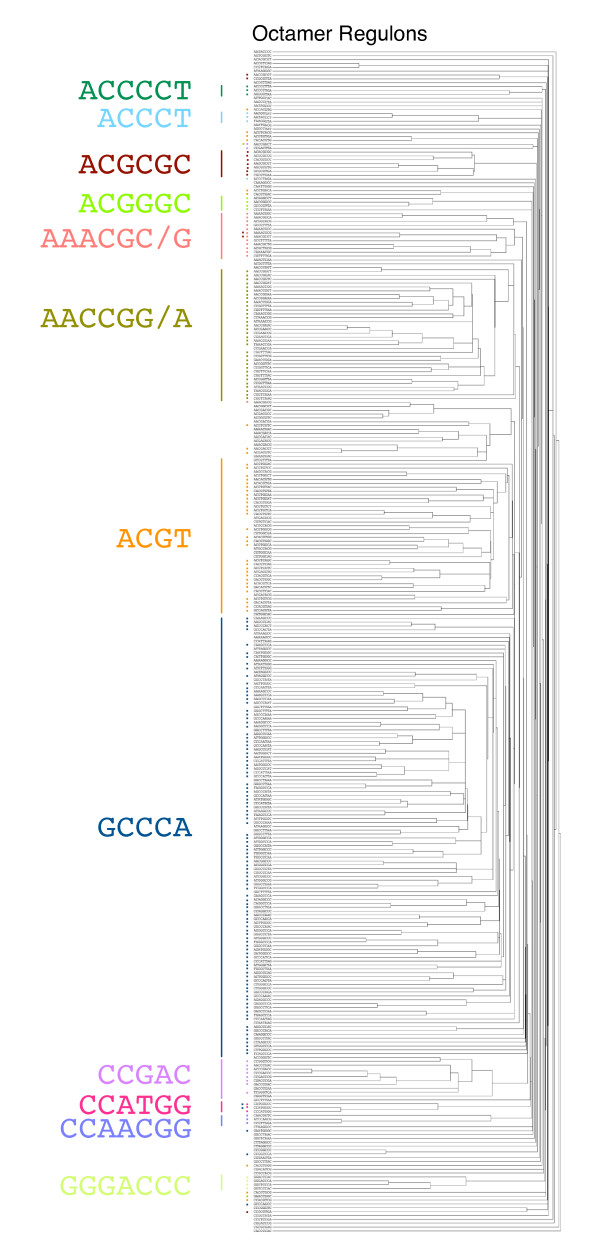
**Clustering of REGs**. Aided by REG-promoter clustering, *Arabidopsis *REGs were subjected to classification. Colored dots in the figure mean presence of the corresponding motif in the REG sequence. The tree is the same as one in Figure 6A.

Clustering of REGs turned out to be reliable as well, and thus useful for REG classification. According to this 2D method, overlapping REGs (e.g., CACGTGGA and ACGTGGAT, Fig. 6C) have a bias toward coexistence by chance. However, similar but mutually exclusive sequences (e.g., ACGTGGAT and ACGTGGAA, Fig. 6C) are also clustered into the same group, suggesting that REGs with the same role are clustered together. This is explained by existence of multiple copies of the same kind of a *cis*-element in a promoter as different octamer expression. Figure 7 shows the whole tree of *Arabidopsis *REGs. This figure demonstrates that REGs with related sequences are clustered together with high reliability. According to these results, 12 motifs have been extracted from *Arabidopsis *REGs (Fig. 7), and are summarized in Table 3.

One group has a GGCCCA core sequence that is known as Site IIa or Element II (Group 1, Table 3). Element II is necessary for cell cycle-related expression and for meristematic expression [35]. Many sequences containing GGCCCA in the center of an octamer were found in REG group of both *Arabidopsis *and rice (Table 4). As seen in the table, this group is a good indicator of conservation.

**Table 4:** Several REG groups were identified from *Arabidopsis *and rice octamer analysis

*Arabidopsis*	Rice
*GGCCCA*	
AGGCCCAA#	AGGCCCAA#
AGGCCCAC#	AGGCCCAC#
AGGCCCAG#	AGGCCCAG#
AGGCCCAT#	AGGCCCAT#
CGGCCCAA#	CGGCCCAA#
CGGCCCAT#	CGGCCCAC
GGGCCCAA#	CGGCCCAG
GGGCCCAG#	CGGCCCAT#
GGGCCCAT#	GGGCCCAA#
TGGCCCAA	GGGCCCAC
TGGCCCAG#	GGGCCCAG#
TGGCCCAT#	GGGCCCAT#
	TGGCCCAC
	TGGCCCAG#
	TGGCCCAT#
**ACGT**, *ACGT***	
ACACGTCA	ACACGTGG#
ACACGTGA	CACGTCAC#
ACACGTGG#	CACGTCTC
CACGTCAC#	CACGTGGC#
CACGTCAG	CACGTGGG#
CACGTCAT	CACGTGTC#
CACGTCTC#	
CACGTGAC	
CACGTGCG	
CACGTGGA	
CACGTGGC#	
CACGTGGG#	
CACGTGGT	
CACGTGTA	
CACGTGTC#	
CACGTGTG	
CACGTGTT	
CCACGTAG	
CCACGTCA	
CCACGTCG	
GACGTCGT	

REGs found in both *Arabidopsis *and rice are indicated with a sharp (hash) symbol. An asterisk indicates any base and is used to restrict the position of the motif in the octamer sequence.

Another group shown in the table has the bZIP protein-binding motif containing ACGT core sequence. This group mediates various environmental signals [36]. Both species have this group in common, but *Arabidopsis *has wider variations than rice (Table 4).

Classification of *Arabidopsis *and rice REGs are shown in Table 3. The largest group is the Group 1, which includes Element II of the *Arabidopsis *PCNA-2 involved in cell cycle-related expression, as mentioned above. As shown in the table, this group is well conserved between *Arabidopsis *and rice and has many members for both species. There are several other REG groups, some of which are rich in only *Arabidopsis *and some are found from both (several examples in Table 4 and summarized in Table 3). Comparison between *Arabidopsis *and rice suggests both conserved and differentiated types of REGs.

The identified *Arabidopsis *REG sequences were referred to the PLACE database that is a collection of reported plant *cis*-regulatory elements [40]. The comparison revealed that 155 out of 308 *Arabidopsis *REGs show 100 % match with at least one of the *Arabidopsis *PLACE entries, giving an estimation that 50 % of the REGs are of established *cis*-regulatory elements (Table S6 [see Additional file 7]). These results again provide strong evidence for biologically meaningful extraction of sequences by the LDSS method. From another point of view, 21 out of 48 *Arabidopsis *PLACE entries have been found in the REG list (Table 5). Comparison with another *cis*-element database, AGRIS [41], resulted in lower match than PLACE (27%) among *Arabidopsis *motif entries shorter than 9 bps (data not shown). These results suggest that not all of the *cis*-regulatory elements are detected by the LDSS strategy. One of the valuable finding of this analysis is the identification of a large number of novel REGs.

**Table 5:** PLACE *cis*-elements found and not found in *Arabidopsis *REGs

	**PLACE discription**	**sequence**
**found**		
1	**ACGTATERD1 **ACGT sequence required for etiolation-induced expression of erd1 (early responsive to dehydration) in Arabidopsis;	ACGT
2	**ABRELATERD1 **ABRE-like sequence (from -199 to -195) required for etiolation-induced expression of erd1 (early responsive to dehydration) in Arabidopsis;	ACGTG
3	**LTRECOREATCOR15 **Core of low temperature responsive element (LTRE) of cor15a gene in Arabidopsis;	CCGAC
4	**SORLIP1AT **one of "Sequences Over-Represented in Light-Induced Promoters (SORLIPs) in Arabidopsis; Computationally identified phyA-induced motifs;	GCCAC
5	**SORLIP2AT **one of "Sequences Over-Represented in Light-Induced Promoters (SORLIPs) in Arabidopsis; Computationally identified phyA-induced motifs;	GGGCC
6	**WBOXATNPR1 **"W-box" found in promoter of Arabidopsis NPR1 gene; They were recognized specifically by salicylic acid (SA)-induced WRKY DNA binding proteins;	TTGAC
7	**CACGTGMOTIF **"CACGTG motif"; "G-box; Binding site of Arabidopsis GBF4;	CACGTG
8	**MYB2CONSENSUSAT **MYB recognition site found in the promoters of the dehydration-responsive gene rd22 and many other genes in Arabidopsis; Y = C/T; K = G/T;	YAACKG
9	**MYBCORE **Binding site for all animal MYB and at least two plant MYB proteins ATMYB1 and ATMYB2, both isolated from Arabidopsis; ATMYB2 is involved in regulation of genes that are responsive to water stress in Arabidopsis;	CNGTTR
10	**SITEIIATCYTC **"Site II element" found in the promoter regions of cytochrome genes (Cytc-1, Cytc-2) in Arabidopsis; Y = C/T;	TGGGCY
11	**ACGTABREMOTIFA2OSEM **Experimentally determined sequence requirement of ACGT-core of motif A in ABRE of the rice gene, OSEM; DRE and ABRE are interdependent in the ABA-responsive expression of the rd29A in Arabidopsis; K = G/T;	ACGTGKC
12	**DPBFCOREDCDC3 **A novel class of bZIP transcription factors, DPBF-1 and 2 (Dc3 promoter-binding factor-1 and 2) binding core sequence; Found in the carrot Dc3 gene promoter; Dc3 expression is normally embryo-specific, and also can be induced by ABA; The Arabidopsis abscisic acid response gene ABI5 encodes a bZIP transcription factor; abi5 mutant have a pleiotropic defects in ABA response; ABI5 regulates a subset of late embryogenesis-abundant genes; GIA1 (growth-insensitivity to ABA) is identical to ABI5;	ACACNNG
13	**GADOWNAT **Sequence present in 24 genes in the GA-down regulated d1 cluster found in Arabidopsis seed germination;	ACGTGTC
14	**WUSATAg **Target sequence of WUS in the intron of AGAMOUS gene in Arabidopsis;	TTAATGG
15	**CDA1ATCAB2 **CDA-1 (CAB2 DET1-associated factor 1) binding site in DtRE (dark response element) f of chlorophyll a/b-binding protein2 (CAB2) gene in Arabidopsis;	CAAAACGC
16	**EMBP1TAEM **Binding site of trans-acting factor EMBP-1; wheat Em gene; Binding site of ABFs; ABFs (ABRE binding factors) were isolated from Arabidopsis by a yeast one-hybrid screening system; Involved in ABA-mediated stress-signaling pathway;	CACGTGGC
17	**HEXAT **"Hex motif" ; Binding site of Arabidopsis bZIP protein TGA1 and G box binding factor GBF1; G-Box-like element;	TGACGTGG
18	**UPRMOTIFIAT **"Motif I" in the conserved UPR (unfolded protein response) cis-acting element in Arabidopsis genes coding for SAR1B, HSP-90, SBR-like, Ca-ATPase 4, CNX1, PDI, etc.;	CCACGTCA
19	**RAV1AAT **Binding consensus sequence of Arabidopsis transcription factor, RAV1; The expression level of RAV1 were relatively high in rosette leaves and roots;	CAACA
20	**DRECRTCOREAT **Core motif of DRE/CRT (dehydration-responsive element/C-repeat) cis-acting element found in many genes in Arabidopsis and in rice; R = G/A;	RCCGAC
21	**ELRECOREPCRP1 **ElRE (Elicitor Responsive Element) core of parsley (P.c.) PR1 genes; consensus sequence of elements W1 and W2 of parsley PR1-1 and PR1-2 promoters; Box W1 and W2 are the binding site of WRKY1 and WRKY2, respectively; W-box found in thioredoxin h5 gene in Arabidopsis (Laloi et al.);	TTGACC
**not found**		
22	**ARR1AT**"ARR1-binding element" found in Arabidopsis; ARR1 is a response regulator; N = G/A/C/T;	NGATT
23	**ARFAT **ARF (auxin response factor) binding site found in the promoters of primary/early auxin response genes of Arabidopsis; AuxRE; Binding site of Arabidopsis ARF1 (Auxin response factor1);	TGTCTC
24	**HEXAMERATH4 **hexamer motif of Arabidopsis histone H4 promoter;	CCGTCG
25	**IBOX **"I box"; "I-box"; Conserved sequence upstream of light-regulated genes; Sequence found in the promoter region of rbcS of tomato and Arabidopsis;	GATAAG
26	**MYB1AT **MYB recognition site found in the promoters of the dehydration-responsive gene rd22 and many other genes in Arabidopsis; W = A/T;	WAACCA
27	**MYB2AT **Binding site for ATMYB2, an Arabidopsis MYB homolog; ATMYB2 is involved in regulation of genes that are responsive to water stress in Arabidopsis;	TAACTG
28	**MYCATERD1 **MYC recognition sequence necessary for expression of erd1 (early responsive to dehydration) in dehydrated Arabidopsis; NAC protein bound specifically to the CATGTG motif (Tran et al., 2004);	CATGTG
29	**MYCATRD22 **Binding site for MYC (rd22BP1) in Arabidopsis dehydration-responsive gene, rd22; MYC binding site in rd22 gene of Arabidopsis; ABA-induction;	CACATG
30	**PREATPRODH **"PRE (Pro- or hypoosmolarity-responsive element) found in the promoter region of proline dehydrogenase (ProDH) gene in Arabidopsis;	ACTCAT
31	**RAV1BAT **Binding consensus sequence of an Arabidopsis transcription factor, RAV1; The expression level of RAV1 were relatively high in rosette leaves and roots;	CACCTG
32	**SREATMSD **"sugar-repressive element (SRE)" found in 272 of the 1592 down-regulated genes after main stem decapitation in Arabidopsis;	TTATCC
33	**TBOXATGAPB **"Tbox" found in the Arabidopsis GAPB gene promoter; Mutations in the "Tbox" resulted in reductions of light-activated gene transcription;	ACTTTG
34	**AGCBOXNPGLB **"AGC box" repeated twice in a 61 bp enhancer element in tobacco (N.p.) class I beta-1,3-glucanase (GLB) gene; "GCC-box"; Binding sequence of Arabidopsis AtERFs;	AGCCGCC
35	**GAREAT **GARE (GA-responsive element); Occurrence of GARE in GA-inducible, GA-responsible, and GA-nonresponsive genes found in Arabidopsis seed germination was 20, 18, and 12%, respectively;	TAACAAR
36	**LEAFYATAG **Target sequence of LEAFY in the intron of AGAMOUS gene in Arabidopsis;	CCAATGT
37	**LTREATLTI78 **Putative low temperature responsive element (LTRE); Found in Arabidopsis low-temperature-induced (lti) genes, lti78/cor78/rd29A and lti65;	ACCGACA
38	**MYBATRD22 **Binding site for MYB (ATMYB2) in dehydration-responsive gene, rd22; MYB binding site in rd22 gene of Arabidopsis thaliana; ABA-induction;	CTAACCA
39	**SORLIP5AT **one of "Sequences Over-Represented in Light-Induced Promoters (SORLIPs) in Arabidopsis; Computationally identified phyA-induced motifs;	GAGTGAG
40	**ABREZMRAB28 **ABRE; ABA and water-stress responses; Found in maize (Z.m.) rab28; maize rab28 is ABA-inducible in embryos and vegetative tissues; Found in the Arabidopsis alcohol dehydrogenase (Adh) gene promoter;	CCACGTGG
41	**CCA1ATLHCB1 **CCA1 binding site; CCA1 protein (myb-related transcription factor) interact with two imperfect repeats of AAMAATCT in Lhcb1*3 gene of Arabidopsis ; Related to regulation by phytochrome;	AAMAATCT
42	**E2FANTRNR **"E2Fa element" found in the tobacco RNR (Ribonucleotide reductase) gene promoter and in the Arabidopsis CDC6 gene promoter; Binding site of tobacco and Arabidopsis E2F; Involved in upregulation of the promoter at G1/S transition;	TTTCCCGC
43	**L1BOXATPDF1 **"L1 box" found in promoter of Arabidopsis PROTODERMAL FACTOR1 (PDF1) gene; Y = C/T;	TAAATGYA
44	**OCTAMERMOTIFTAH3H4 **"Octamer motif" found in promoter of wheat histone genes H3 and H4, and corn histone genes H3 and H4; Arabidopsis histone H4; "histone-specific octamer";	CGCGGATC
45	**PIATGAPB **"PI" found in the Arabidopsis GAPB gene promoter; Mutations in the "PI" resulted in reductions of light-activated gene transcription;	GTGATCAC
46	**RYREPEATVFLEB4 **"RY repeat motif"; quantitative seed expression; Gene: Vicia faba LeB4; Soybean glycinin (Gy2); other dicot and monocot seed protein genes; Binding site of Arabidopsis B3-domain-containing transcription factor FUS3;	CATGCATG
47	**UP2ATMSD **"Up2" motif found in 193 of the 1184 up-regulated genes after main stem decapitation in Arabidopsis; W = A/T;	AAACCCTA
48	**ZDNAFORMINGATCAB1 **"Z-DNA-forming sequence" found in the Arabidopsis chlorophyll a/b binding protein gene (cab1) promoter; Involved in light-dependent developmental expression of the gene; "Z-box";	ATACGTGT

### Characterization of transcription start site

**Figure 8:**
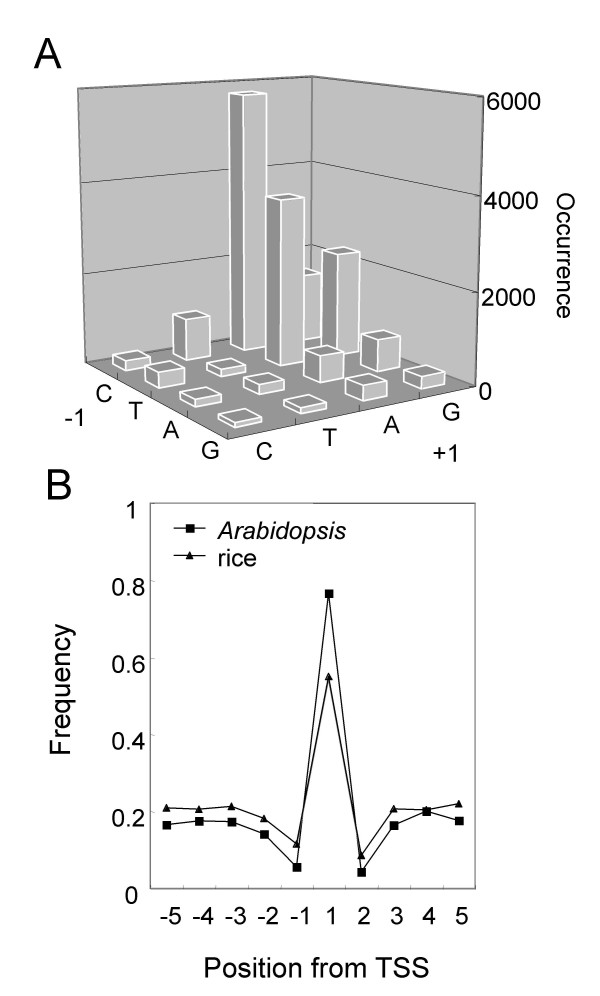
**Identification of YR Rule**. (A) Dinucleotide sequences at the -1/+1 position relative to *Arabidopsis *TSS, determined by information of the fl-cDNAs, were counted. As shown, most of the TSS have (C/T)(A/G), and this YR Rule applies to 77% of the analyzed TSSs. (B) Frequency of dinucleotide sequences fitting with YR Rule was scanned from -5 to +5 of *Arabidopsis *and rice TSS. Position of the downstream site of the dimer is shown. For example, the -1/+1 position is indicated as "1". Theoretically frequency of YR in non-biased sequence is 0.25.

We then analyzed sequence characteristics around the TSS. In this region, the Initiator motif (Inr: YYAN(T/A)YY, TSS is underlined) is known in some mammalian promoters [1], and it is also functional in plants [42]. A survey of *Arabidopsis *TSS revealed that a limited number of promoters (less than 10%) have the Inr motif around the TSS. Thus, we looked for a more general rule. We surveyed which base is preferred at the -1/+1 position among *Arabidopsis *TSS. The most frequently observed sequence was CA (TSS is underlined), and TA was the second. As summarized in Figure 8A, there is a strong preference of a dimer sequence at the -1/+1 position. The graph clearly shows most of the TSS is A or G, and the -1 position is likely to be C or T. This "YR Rule" (YR, TSS underlined, Y: C or T, R: A or G) applies to as many as 77% of the *Arabidopsis *promoters that is a much higher frequency than expected random appearance (25%). Similar analysis for the -2/-1 and +1/+2 positions did not reveal clear extension of the rule. When the YR Rule was applied to the -6/-5 to +4/+5 positions, we found that the ratio of YR Rule-positive is highest at the -1/+1 position in the local region examined (Fig. 8B, *Arabidopsis*). The figure shows that this rule is also applicable to rice TSS (Fig. 8B, rice). These analyses have revealed that sequence preference at TSS is well conserved between *Arabidopsis *and rice.

### An example of *Arabidopsis *promoter

**Figure 9:**
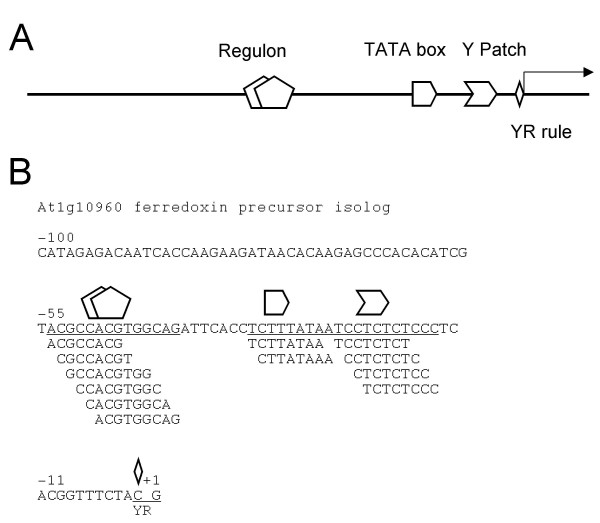
**Illustration of YR Rule, Y Patch, TATA box, and REG**. (A) Expected appearance positions relative to the TSS are as follows: YR Rule (-1/+1), Y Patch (-100 to -1), TATA box (-50 to -20), REG (-20 to -400). Among them, only the REG is orientation-insensitive, and the other groups are sensitive. In many cases the Y Patch locates between the TATA boxes and the TSS, but it is also observed upstream of the TATA boxes. (B) An example of an *Arabidopsis *promoter that has a Y Patch and TATA box. At1g10960 is one of the promoters clustered in Figure 6B. The promoter sequence from -100 to +1 is shown together with octamer motifs. Marks on the sequence are the same as illustrated in (A).

Our simple LDSS analysis has successfully revealed three distinct groups consisting of hundreds of short sequences. Figure 9A illustrates the architecture of plant promoters based on these findings.

Tight positioning of the TATA boxes relative to the TSS fits with the general idea that the TATA boxes determine the position of the TSS. In addition, the YR Rule of *Arabidopsis *would be another important determinant as well. The Y Patches locate between the TATA boxes and the TSS, but they can be upstream of the TATA boxes, considering the wide distribution profiles (Figure 5). The role of the Y Patch is not known. The above three elements are orientation-sensitive, and constituents of a core promoter. REGs appear upstream of the TATA box, and they exist in an orientation-insensitive manner. Rice promoters share the above characteristics, showing architectural conservation between dicots and monocots.

An example of an *Arabidopsis *promoter that has the Y Patch and TATA box is shown in Figure 9B. Octamer analysis of the promoter revealed one cluster of Group 2 REGs (Table 3), one cluster of Y Patches, one cluster of TATA box, and YR Rule. An interesting feature of the figure is the multiple hits of a locus, detecting a longer element. This demonstrates that octamer analysis can detect long functional units as clusters of octamers.

## Discussion

### Characteristics of LDSS analysis

In this study, we have identified hundreds of novel sequences solely based on local distribution in the promoter region of *Arabidopsis *and rice. Biological information, such as microarray data, was not used at all for sequence extraction, and it becomes useful only during interpretation of the extracted sequence. This method is equally sensitive in detection of major and minor motifs in a promoter population as demonstrated by simultaneous detection of major TATA elements and minor REG elements. This feature is an advantage of the LDSS method over other methods of detection of consensus sequences among promoter populations, such as Gibbs Sampling method. We successfully applied the LDSS method to *Arabidopsis *and rice promoters, and of course, it is applicable to bacterial and mammalian research as well.

The observed localized distribution is a direct result of the selection pressure. While the localization is an indication of a beneficial role for the organism, the relationship between local distribution of a sequence and its functionality is indirect. Therefore, the question arises if all regulatory elements can be picked up by the LDSS strategy.

When we compared REG sequences with established *cis*-elements in the PLACE database, it was found that 27 out of 48 *Arabidopsis *PLACE entries are absent in the extracted REGs (Table 5). These results indicate that not all of the functional elements are LDSS-positive, and thus some would not be detected by this method. There are two possibilities for the presence of *cis*-elements that do not show local distribution. One possibility is that these elements are relatively "new" so there has not been selection pressure for a long enough period. Another possibility is that there has not been any selection pressure because of functional differences from the LDSS-positive elements. The latter idea suggests localization-insensitive classes of regulatory elements that are distinct from REGs. So called long range-regulators [43,44] might be one of the classes.

Generally, any functional sequences in the genome are recognized by *trans*-acting factors that are DNA-binding proteins. Promoter elements and their *trans-*factors have a relationship of co-evolution. Therefore, differentiation of REGs in the two species would reflect a different status of the corresponding *trans*-factors. Functional comparison of DNA-binding proteins of *Arabidopsis *and rice is expected to give some answers as to why these two species have differentiated REG sequences. As for the conserved REGs, it is reasonable that cell cycle-related elements (Group 1, Table 3) comprise the most conserved group, because the cell cycle is one of the most conserved activities in organisms.

REG sequences can be extracted form mammalian promoters as well. However, our preliminary analyses suggest that the LDSS method can detect much less REGs than of plants (YYY and JO, unpublished results). This may be reflected by different promoter architecture between plants and animals.

### Y Patch

The discovery that the Y Patch is conserved in monocots and dicots is one of the major achievements of this study. A related motif is reported by Molina and Grotewold from *Arabidopsis *core promoter analysis using the Gibbs-sampling method (Motif 1 with a typical sequence, TTCTTCTTC, [29]). The biochemical role of Y Patch is not known, but its position, direction sensitivity, and its abundant nature strongly suggest that it is a general component of the core promoter. Our LDSS analyses suggest that human and mouse do not share this element with plants and thus this is a plant-specific core element (YYY and JO, unpublished results).

### YR Rule

At the TSS, the Initiator (Inr) motif (Y Y A N T/A Y Y, TSS is underlined) is known as a recognition site by TFIID [3]. Following their rules, the YR Rule can be considered as a less stringent form of Inr. According to this point of view, the YR Rule might be recognized by TFIID. The high coverage of the YR Rule is a useful feature for prediction of TSS. Recently, Carninci et al., have reported the same rule is applicable to mouse and human promoters as well [45], revealing conservation of YR Rule between plants and mammals.

This rule is not an artifact by the Cap-Trapper method that is the basis of TSS mapping of this study and mammalian studies mentioned above [45], because it is applicable to human TSS determined by another method (Oligo-Cap method, [46]) as well (YYY and JO, unpublished results).

A plant consensus around TSS (A/T n T/a C/t A/c a/t, TSS is underlined) is reported by Shahmuradov *et al *based on 217 dicot promoters (actual consensus is expressed by a matrix, [47]). This consensus also largely overlaps with YR Rule.

The TFIIB-Recognition Element (BRE) is another core promoter element of animal genes. It is located just upstream of the TATA box and has a GC-rich sequence, (G/C)(G/C)(G/C)CGCC [1,48]. Our analysis did not detect the BRE as a LDSS-positive element, although CC is preferred at the neighboring sequence of the TATA box at the upstream side in both *Arabidopsis *and rice promoters (Table S2 [see Additional file 3] and S3 [see Additional file 4]).

### LDSS analysis provides useful information toward precise promoter prediction

The hundreds of octamer sequences identified by the LDSS analysis can be used for promoter prediction. The presence of the TATA box is an important feature of a promoter, but there are many false-positives in the genome. For example, a TATA octamer sequence with the highest specific localization is found within the peak area 30% of times in the promoter region, meaning that 70% are found outside of the peak area. This is essentially consistent with a previous study, where more than 200,000 putative TBP-binding sites were detected from the *Arabidopsis *genome [27]. Utilization of preferential sequence around the TATA box, and coexistence with the Y Patch and REG are expected to elevate accuracy of prediction. Although such a combinational approach is incorporated into several promoter prediction programs [13], motifs to be detected have been limited so far. Our long list of the LDSS-positive octamers is expected to serve as a thick dictionary for precise interpretation of plant genomes.

## Conclusion

In this report, we showed that LDSS can be applied to plant genomes. We have successfully extracted hundreds of promoter elements as LDSS-positive octamers. All the observed behaviors of the isolated elements suggest functionality of these elements. Promoter architectures of monocot and dicot revealed in this study are well conserved, but there are moderate variations in the utilized sequences.

## Methods

### Preparation of promoter databases

Cap-Trapper [49] is one of the most reliable methods for identification of the 5' end of mRNA and thus suitable for determination of TSS. So-called full-length (fl) cDNAs of *Arabidopsis *and rice were made by the Cap-Trapper method, and around ten to twenty thousand of non-redundant fl-cDNA clones for each species have been completely sequenced [50,51]. Therefore, we decided to use the information from the fl-cDNAs for positioning of promoters. Genome sequences of promoter regions from -1,000 to -1 bp were prepared with the aid of information of the 5' ends of fl-cDNAs of *Arabidopsis *[50,52] and rice [51]. The established *Arabidopsis *promoter database [50,53] and a rice database with 11,370 promoters, prepared in this study, were utilized for our analysis.

Positions of rice fl-cDNA clones of rice [51] were mapped on to corresponding BAC clones according to description of "MappingData.txt" obtained from the KOME web site [54], and promoter regions from -1 kb to +200 bp relative to the TSS, that are 1.2 kbp long, were collected. BAC and fl-cDNA sequences were obtained from DDBJ. Special care was taken for 5' end of fl-cDNA sequences, and ones with less than 2 bp mismatch with the corresponding genomic sequences were used for the promoter mapping. Sequences of non-redundant 11,370 rice promoters have been prepared. For analyses of the TSS region, as shown in Figure 6, rice fl-cDNA sequences with no mismatch to the 5' end (6,209 promoters) were used. Establishment of the *Arabidopsis *promoter database is described elsewhere [50,53]. Earlier analyses with *Arabidopsis *hexamers have been done using the distributed database containing 15,607 promoters. This database is based on distinct TSS and allows multiple promoters belonging to a single gene. A smaller set of 12,951 promoters was re-selected from the 15,607-version so as to pick-up one promoter from one gene, and used for octamer analyses. For preparation of random genomic fragments, non-overlapping *Arabidopsis *BAC clones were selected by consulting a TAIR web site [55], they were successively cut into 1 kb pieces and serial numbers were given to the fragments. Sequences corresponding to 3,000 randomly chosen numbers based on the Mersenne Twister method [56] were used as random genomic fragments of 1 kb length.

The programs used in this study will be freely provided upon request for non-profit purposes. A searchable web site to obtain results in this work will be released.

### Generation of random distribution

Random distribution samples were generated with respect to Total Area, that is indication of total count in a promoter database. For each Total Area, 1,000 samples were prepared, and their RPA values were subjected to statistical analysis. Average and standard deviation are functions of Total Area (Figure S1 [Additional File 2]) and affected by a smoothing window. Model RPA populations of random distribution were calculated as the following equations:

REG detection (smoothing with a 21-bin (width of window), and Total Area < 2,000): log_10_(average) = -0.1861Ln(Total Area) – 0.5329, SD = 0.17 CORE detection (smoothing with a 3-bin, and Total Area < 10,000): log_10_(average) = -0.1784Ln(Total Area) – 0.8026, SD = 0.13

These models were utilized for estimation of p value for each octamer distribution.

### Sequence analysis

Sequence analysis was achieved by a combination of home-made Perl and C^++ ^programs and also Excel software (Microsoft Japan, Tokyo). The first step of the analysis was the preparation of index files for each promoter with all the possible 4,096 hexamer and 65,536 octamer sequences. Information of the index files was then rearranged for each hexamer and octamer sequence, and the occurrence of the short sequences was summarized according to the promoter position. Summarized distribution data of each hexamer was then subjected to smoothing with a bin of 15 bp. Generally, smoothing with a wide bin lowers the peak height of a sharp peak, and with a narrow bin capturing a wide and low peak is not always possible. Considering these tendencies, a bin of 21 bp was used for identification of octamer REGs, and a bin of 3 bp was used for octamer core elements. Octamer REGs were extracted after merging the distribution data of the complementary sequence to increase the count of occurrence. As for extraction of octamer Core elements that is orientation-sensitive, merging was avoided. Positions of octamers and hexamers were counted from the first base of the sequence. For example, the position of a hexamer sequence that locates from -6 to -1 is expressed as -6. Positions of average values for line smoothing are indicated at the centre of the region. Therefore, positions closest to TSS vary depending on the bin length as well.

Thresholds for distribution of peaks are as follows:

Hexamer: (peak height/Base Line > 3) & (peak height/SD > 5) & (Peak Area/basal fluctuation) > 5),

Octamer Core: (p value < 10^-4^) & (peak height/Base Line > 5) & (peak height/SD > 10) & (Peak Area/basal fluctuation > 6) & (peak position > -51),

Octamer REG: (p values < 10^-4^) & (peak height/Base Line > 3) & (Peak Area/total area > 0.1) & (peak height/SD > 5) & (Peak Area/basal fluctuation > 6) & (peak position <-50).

Fitting the distribution data with the Gaussian curve was achieved using Igor Pro (Hulinks, Tokyo). All the LDSS-positive octamers together with above parameters can be viewed at our web site ([57]).

Clustering analyses were achieved with Cluster [58] and visualized with TreeView [59]. For clustering of LDSS-positive elements based on distribution profiles, peak value of each profile was adjusted to 5.0. For REG-promoter clustering, number of each REG appeared at a region between -400 to -40 bp was scored for each promoter and a REG-promoter table was prepared. Among the Cluster options, the hierarchical clustering method (centroid linkage) gave the most natural results over the *k*-means and SOM methods.

Among the PLACE database [40], 48 entries with definition sequences of 8 bases or less and also with description containing "Arabidopsis" were subjected to REG survey.

## Abbreviations

LDSS – Local Distribution of Short Sequences

TSS- transcription start site

## Authors' contributions

YYY designed and performed the analyses including writing Perl programs. HI and TA prepared rice promoter database and wrote C^++ ^programs. MM, TS, MSatou, MSeki, and KS prepared *Arabidopsis *promoter database. JO contributed in identification of YR Rule. All authors read and approved the final manuscript.

## Supplementary Material

Additional file 1 Complete list of LDSS-positive hexamers of Arabidopsis (Table S1.pdf). Contains hexamer sequences and parameters. Click here for file

Additional file 2 Characteristics of random distribution (FigS1.pdf). Contains graphs to show relationship between a LDSS parameter and a size of population (Total Area). Click here for file

Additional file 3 Arabidopsis core octamers (Table S2.pdf). Contains octamer sequences and parameters. Click here for file

Additional file 4 Rice core octamers (Table S3.pdf). Contains octamer sequences and parameters. Click here for file

Additional file 5 Arabidopsis REG octamers (Table S4.pdf). Contains octamer sequences and parameters. Click here for file

Additional file 6 Rice REG octamers (Table S5.pdf). Contains octamer sequences and parameters. Click here for file

Additional file 7 Relationship between Arabidopsis REG and PLACE entry (Table S6.xls). A table showing which octamer REG corresponds to which PLACE entry, and vice versa. Click here for file
