# Supplementary material for: Identification of plant promoter constituents by analysis of local distribution of short sequences
Source: BMC Genomics. 2007 Mar 8;8:67. doi: 10.1186/1471-2164-8-67 (PMC1832190; doi:10.1186/1471-2164-8-67)
Supplement: Additional file 1 — Complete list of LDSS-positive hexamers of Arabidopsis (Table S1.pdf). Contains hexamer sequences and parameters. [file 1471-2164-8-67-S1.pdf]

Table S1 Complete list of LDSS-positive hexamers of Arabidopsis

Sequence	Peak position	Peak width	RPH	RPA	Peak Area/basal fluctuation	Peak hight-Base line)/sd	Occurence/ Promoter
Y Patch							
TCTCTC	-13	158	10.957	0.248	21.554	85.602	0.621
CCTCTC	-13	107	8.132	0.196	17.656	47.494	0.219
CTTCTC	-13	88	7.640	0.146	24.684	73.983	0.476
CTCCTC	-13	81	7.233	0.121	14.686	45.880	0.226
CTCTTC	-13	91	7.020	0.141	16.948	47.119	0.433
CTCTCC	-13	108	6.951	0.155	13.863	43.499	0.224
TCCCTC	-13	93	6.130	0.149	11.754	26.398	0.144
CGCCTC	-13	93	5.869	0.145	6.404	14.451	0.053
TTCTTC	-13	75	5.781	0.112	20.336	49.874	0.897
TTCCTC	-13	76	5.750	0.100	12.297	35.972	0.345
TCTTCC	-13	77	5.662	0.109	14.086	34.758	0.374
TCTCCC	-13	128	5.538	0.132	7.509	23.176	0.159
TCCTCT	-13	102	5.485	0.126	13.907	37.109	0.341
CTCCGC	-13	72	5.170	0.105	6.749	14.457	0.055
TTCTCC	-13	84	5.004	0.084	11.454	36.129	0.361
TCCTCC	-13	61	4.955	0.091	11.603	24.780	0.206
CTTCTT	-13	77	4.883	0.111	21.725	45.347	0.813
CGTCTC	-13	103	4.864	0.122	7.358	18.187	0.133
CTTCCC	-13	73	4.828	0.106	10.498	21.883	0.152
CTCCCC	-13	52	4.734	0.092	7.125	9.617	0.058
ACTCTC	-13	87	4.658	0.102	11.177	25.898	0.277
TCCTTC	-13	82	4.648	0.108	13.532	29.667	0.288
CGCTCT	-13	75	4.447	0.069	5.043	14.516	0.068
TTCCCC	-13	58	4.323	0.082	8.533	14.041	0.127
TCTCGC	-13	58	4.307	0.061	5.500	13.638	0.091
CCTCTT	-13	64	3.933	0.092	12.224	19.078	0.267
CCTTTC	-13	84	3.427	0.064	5.122	13.637	0.215
CTCGCC	-13	19	3.424	0.029	6.482	8.327	0.055
CTTTCC	-13	91	3.281	0.067	6.426	16.120	0.244
CTCGTC	-13	100	3.266	0.090	5.401	10.983	0.107
TTCTCT	-13	109	5.767	0.120	18.742	56.966	0.781
TCTCTT	-13	98	4.966	0.110	16.214	42.212	0.786
TTTCTC	-13	103	4.666	0.082	10.745	36.662	0.762
CTCACT	-13	70	4.641	0.095	10.477	21.745	0.203
TCACTC	-13	73	4.583	0.091	10.262	22.459	0.238
TCTCAC	-13	89	4.182	0.087	8.256	20.802	0.278
TCTTTC	-13	94	4.167	0.076	11.943	34.639	0.620
GTCTTC	-13	85	4.112	0.090	10.494	22.650	0.256
CTCTGC	-13	55	3.839	0.055	5.893	14.526	0.146
TTCGTC	-13	83	3.799	0.107	10.503	16.126	0.206
CTCTTT	-13	95	3.652	0.083	12.717	26.062	0.644
ATCTCT	-13	93	3.458	0.085	11.588	23.898	0.538
TTCGCT	-13	75	3.295	0.068	5.243	10.263	0.106
CATTTC	-13	18	3.076	0.019	11.698	19.123	0.426
CTCTCT	-14	256	9.833	0.263	12.881	68.138	0.590
TCTTCT	-14	83	6.096	0.132	28.357	64.274	0.915
CTTCCT	-14	79	5.867	0.124	15.450	35.903	0.294
CCCTCT	-14	80	5.714	0.148	11.048	20.570	0.127

TCTCCT	-14	84	5.163	0.114	14.538	34.533	0.335
CCTCCT	-14	59	5.141	0.119	14.893	24.066	0.154
CTCTCG	-14	80	4.668	0.091	8.435	21.445	0.124
TCTCCG	-14	73	3.734	0.060	6.267	16.997	0.145
CGTCTT	-14	118	4.893	0.131	9.328	24.130	0.175
CACTCT	-14	74	4.716	0.099	9.784	21.627	0.229
CTTTCT	-14	93	4.313	0.078	10.082	30.155	0.609
CTTCAC	-14	96	3.948	0.073	7.208	20.706	0.275
TCGTCT	-15	87	5.351	0.130	13.044	24.894	0.206
CCTTCC	-15	94	5.034	0.127	11.242	24.937	0.129
GTCTCC	-15	75	3.534	0.079	6.056	11.568	0.140
CTCTCA	-15	93	5.108	0.108	10.467	28.157	0.319
TCCTCA	-15	71	3.130	0.049	5.717	15.106	0.225
CTCCCT	-16	81	4.923	0.128	10.080	19.325	0.108
GTCGTC	-16	169	3.441	0.124	5.972	14.330	0.129
CTCTGT	-16	73	3.406	0.064	7.408	16.024	0.274
CCTTCT	-17	83	4.280	0.109	9.901	19.488	0.265
CCGTCT	-17	72	3.703	0.080	5.669	10.638	0.082
CTTCGT	-17	98	4.697	0.106	9.331	24.025	0.183
CGCTTC	-18	77	3.695	0.084	5.527	10.794	0.084
CTTCGC	-18	88	3.663	0.092	5.962	10.854	0.074
TCTCTG	-18	78	3.394	0.071	10.038	20.172	0.350
CCTCAC	-19	76	4.958	0.098	7.740	17.304	0.114
CGCTCC	-19	20	3.802	0.035	7.036	8.482	0.038
TCCCCG	-19	23	3.554	0.039	5.138	6.855	0.035
CTCCCA	-19	64	3.107	0.064	5.869	10.037	0.130
GCCTCT	-19	76	3.087	0.076	6.226	10.281	0.111
GTCTCT	-19	99	3.943	0.091	8.408	19.607	0.315
CTCCGT	-19	72	3.885	0.072	5.307	11.786	0.093
ACCTCT	-19	89	3.154	0.074	7.028	14.424	0.185
CCCTTT	-19	122	3.075	0.100	7.993	14.288	0.206
CCCCTC	-20	68	6.622	0.159	9.164	15.417	0.051
CCCTCC	-20	85	5.387	0.178	13.266	19.028	0.073
CCGCTC	-20	22	3.225	0.030	5.480	6.947	0.035
TCTTCG	-20	56	3.811	0.072	9.913	16.945	0.219
CCTTCG	-20	53	3.526	0.060	5.985	10.894	0.074
TTCGCC	-20	20	3.027	0.026	7.191	8.789	0.080
CCCTTC	-21	117	5.246	0.144	6.331	15.810	0.104
CATCTC	-21	98	3.465	0.078	5.795	14.369	0.281
CACTCC	-21	66	3.212	0.086	6.358	8.380	0.102
AGTCTC	-21	82	3.248	0.072	6.615	13.833	0.218
ACCCCT	-22	210	5.208	0.196	5.150	15.015	0.067
CCACTC	-22	95	4.425	0.117	7.497	15.718	0.136
CCCTCA	-22	87	3.904	0.100	7.502	13.846	0.088
ACCCTC	-23	106	4.571	0.145	8.802	16.295	0.097
CCCCTT	-24	88	4.074	0.125	7.448	11.644	0.082
ACCCTT	-24	158	3.746	0.136	7.512	17.055	0.169
CTCCTT	-42	82	4.348	0.103	13.469	26.977	0.277
TCCCCT	-42	58	3.829	0.094	9.015	11.866	0.093
TTCCCT	-42	75	3.169	0.083	8.760	13.300	0.219
TCGCTT	-43	85	3.091	0.074	5.641	10.261	0.125
TCCCCC	-48	56	4.394	0.091	7.090	10.158	0.049
CACCTC	-48	87	3.367	0.073	5.119	11.761	0.113

CCGCCT	-49	63	4.629	0.105	5.536	10.022	0.050
CCCCAC	-49	206	3.864	0.190	5.148	10.280	0.083
TATA Box-related							
AATACC	-31	22	3.086	0.026	9.425	12.828	0.213
AAATAC	-33	23	3.478	0.030	15.121	21.141	0.599
TAAATA	-34	25	4.650	0.043	26.079	38.206	1.164
ATAAAC	-34	29	3.970	0.039	23.190	35.144	0.696
ATAAAA	-34	574	3.287	0.185	5.926	28.588	1.800
ATATAT	-35	24	3.840	0.039	28.718	41.848	1.463
ATATAA	-35	27	6.139	0.068	48.968	75.988	1.194
TATAAG	-35	24	3.969	0.041	19.662	28.310	0.394
TATAAA	-35	30	9.025	0.102	68.633	114.653	1.307
ATAAAT	-35	27	5.135	0.051	29.819	48.840	1.312
TTATAT	-36	23	3.102	0.027	16.446	23.270	1.083
TATATA	-36	27	6.382	0.075	55.721	84.089	1.414
TTATAA	-36	23	3.362	0.030	17.767	25.244	0.990
CTTATA	-37	24	4.127	0.042	15.780	22.795	0.408
CTATAA	-37	28	8.796	0.102	42.130	64.164	0.440
CTATAT	-37	27	7.491	0.091	34.715	51.835	0.513
GCTATA	-37	23	4.610	0.053	12.938	16.781	0.176
ACTATA	-37	22	3.790	0.037	16.307	22.682	0.437
TCTATA	-38	29	7.233	0.086	36.709	58.987	0.483
CCTTAT	-38	155	3.871	0.098	5.508	16.360	0.216
CGTATA	-38	29	3.175	0.028	6.898	11.495	0.165
GTATAT	-38	23	3.561	0.034	17.015	23.198	0.504
CTTTAT	-38	32	3.553	0.036	16.085	26.461	0.549
GGCTAT	-39	27	3.265	0.030	6.243	9.852	0.114
CCTATA	-39	29	9.560	0.115	30.441	50.313	0.195
CCCTAT	-39	83	6.157	0.089	6.274	23.365	0.099
CCTCTA	-39	71	3.233	0.056	5.163	12.388	0.151
CCTTTA	-39	128	3.950	0.081	5.313	18.492	0.249
GCCTAT	-40	27	5.899	0.062	12.332	18.427	0.098
CTCTAT	-40	60	5.878	0.090	17.336	42.243	0.322
CGCCTA	-40	39	5.069	0.069	6.965	12.645	0.040
CACTAT	-40	39	3.503	0.036	7.992	17.646	0.223
TCCTAT	-40	29	4.218	0.044	13.619	22.217	0.196
TCTCTA	-40	97	3.759	0.078	10.993	28.389	0.445
CTCCTA	-41	46	3.225	0.047	6.998	12.950	0.134
TCCCTA	-41	43	3.022	0.036	5.488	10.761	0.128
CCCTTA	-41	124	4.278	0.120	5.724	13.567	0.116
CTTCTA	-41	31	3.077	0.030	9.058	15.294	0.305
CCACTA	-41	98	3.047	0.068	5.905	12.139	0.190
REG							
ACACGC	-51	157	3.817	0.180	6.849	11.012	0.079
ACCCGG	-53	198	4.458	0.225	5.911	11.471	0.050
CCTCCC	-53	57	4.365	0.113	7.890	10.260	0.064
CCCACC	-54	186	4.527	0.167	5.859	14.092	0.093
CGCGTT	-57	169	3.524	0.175	5.155	8.209	0.059
CCGCGT	-58	190	5.061	0.243	5.269	9.651	0.036
GCGCGT	-59	244	5.556	0.321	5.938	11.044	0.044
CGACCC	-59	126	5.894	0.188	7.519	15.152	0.060
GTCGTT	-59	226	3.695	0.152	6.094	16.064	0.176
CGTCGT	-60	192	4.408	0.190	5.929	13.075	0.136

TACCCT	-60	106	3.114	0.106	6.023	8.891	0.108
GCCCAC	-60	195	4.943	0.221	5.930	12.555	0.077
CCGACC	-60	201	5.974	0.218	6.628	18.121	0.071
GACGTC	-61	108	3.992	0.134	7.476	12.917	0.089
CGGCCC	-62	189	7.658	0.356	8.677	14.986	0.050
ACGTCG	-62	203	4.155	0.178	5.712	13.362	0.094
CACGTC	-62	243	4.054	0.230	5.589	10.622	0.110
CGCGCG	-64	147	7.457	0.249	5.339	8.752	0.017
GAGCCC	-64	197	4.411	0.209	5.050	9.862	0.055
CCCGAC	-64	148	5.053	0.176	5.851	12.900	0.045
ACGCGC	-65	190	5.080	0.305	7.665	10.450	0.046
ACGACG	-65	209	3.527	0.161	5.599	11.706	0.129
CGCCAC	-66	226	4.161	0.198	5.055	11.839	0.078
CGACGT	-67	183	3.949	0.184	5.975	11.579	0.091
CCGGTT	-67	294	4.642	0.267	7.369	17.834	0.188
GCCGTT	-71	162	3.032	0.145	5.244	7.813	0.093
GCCCAA	-72	366	7.775	0.421	9.854	28.535	0.244
GACCCG	-72	100	5.920	0.189	10.563	16.892	0.065
GGCCCA	-73	347	12.255	0.528	17.190	44.968	0.248
GCACGT	-73	133	3.477	0.133	5.161	8.362	0.065
ACCCGA	-74	106	3.692	0.111	6.706	11.913	0.097
ACGTCA	-74	193	3.432	0.172	6.555	11.665	0.157
TGGCCC	-75	194	4.801	0.237	7.974	14.689	0.084
CCCAAT	-75	314	3.867	0.242	8.630	19.827	0.271
CCCATT	-75	238	3.939	0.221	8.893	17.435	0.274
TAGGCC	-75	311	6.183	0.337	8.214	19.678	0.099
GCCCAT	-76	320	8.416	0.428	13.223	35.172	0.228
AGGCCC	-76	326	14.777	0.544	14.413	42.859	0.149
GCCCAG	-76	155	4.262	0.196	5.091	7.500	0.049
AAGGCC	-77	287	6.272	0.326	8.384	20.674	0.137
AGGGTA	-79	148	3.964	0.123	5.088	13.121	0.109
ACGTGT	-79	249	4.907	0.283	10.987	21.026	0.233
CGTGTC	-79	289	5.572	0.334	7.746	16.683	0.134
CACGTG	-80	273	6.851	0.375	15.353	32.495	0.242
ACACGT	-81	251	4.059	0.242	7.608	14.457	0.229
GCCACG	-83	215	6.642	0.309	8.429	18.826	0.096
CCACGT	-83	260	5.662	0.349	11.134	20.800	0.174
CCCATA	-83	245	3.307	0.184	5.997	11.953	0.183
GGGCCG	-85	196	6.007	0.302	7.219	12.514	0.050
ATTGGG	-85	244	3.227	0.162	6.202	13.988	0.229
AGCCCA	-85	284	7.525	0.393	14.884	35.664	0.238
AAGCCC	-86	299	7.480	0.373	12.510	33.600	0.197
AAAGCC	-87	224	3.305	0.179	7.181	14.233	0.299
ACCGGT	-89	167	3.885	0.210	7.385	10.078	0.148
GGGCCC	-91	256	9.513	0.442	8.893	18.489	0.047
AACGGC	-92	227	3.567	0.218	5.710	9.407	0.114
GGGCTT	-95	195	4.788	0.236	10.297	19.303	0.174
AACCGG	-95	258	5.134	0.295	10.590	21.625	0.195
ATGGGC	-97	295	6.294	0.348	11.985	30.311	0.220
ACGTGG	-97	248	4.611	0.297	11.758	20.033	0.164
CGTGGC	-97	251	5.963	0.344	10.873	21.195	0.100
CAACGG	-97	192	3.402	0.172	5.291	9.825	0.127
AATGGG	-98	194	3.361	0.165	8.570	16.840	0.261

GGCCTG	-98	183	5.649	0.223	5.790	13.925	0.050
TGGGCT	-99	238	5.353	0.273	9.081	20.570	0.221
TCCACG	-102	231	3.359	0.198	6.019	10.717	0.132
AAACCG	-102	264	3.353	0.196	8.307	15.891	0.323
AGGGGT	-103	108	3.588	0.118	5.587	9.410	0.069
TAACCG	-103	157	3.084	0.135	7.356	11.352	0.156
ACGCGT	-105	147	3.486	0.167	5.006	6.920	0.073
ACGGCC	-105	180	4.000	0.204	5.194	8.837	0.047
GTGGCA	-105	178	3.143	0.132	5.047	10.311	0.132
GGGCCT	-106	240	10.314	0.469	17.814	35.438	0.130
GGCCTT	-106	221	3.770	0.218	7.097	12.660	0.128
TGGGCC	-107	262	9.288	0.456	20.513	42.269	0.229
GGGCCA	-107	157	5.625	0.228	8.571	16.437	0.084
TTGGGC	-109	234	5.677	0.298	11.792	24.547	0.218
CTGGGC	-111	143	4.679	0.192	6.589	12.040	0.045
GGCCTA	-111	260	5.148	0.274	8.192	18.665	0.102
GACACG	-127	249	4.699	0.238	5.487	12.715	0.114
CGACAC	-127	163	3.146	0.127	5.268	10.402	0.090
CACGCG	-138	182	5.215	0.299	8.382	11.490	0.061
CGGGCC	-147	147	6.943	0.256	6.416	11.541	0.025
others							
CTCGAG	-13	19	3.533	0.018	5.292	12.653	0.109
TCACAC	-13	111	3.194	0.086	7.073	14.345	0.249
CCTCGA	-14	20	3.010	0.023	6.262	9.470	0.081
CGTCAC	-16	217	3.029	0.186	5.984	9.543	0.110
CCCCAA	-17	109	3.617	0.078	5.439	14.457	0.159
CCCTAA	-18	66	3.513	0.097	11.046	13.358	0.198
CTCACA	-18	75	3.122	0.052	5.085	12.104	0.228
ACCCTA	-19	117	4.561	0.151	9.459	18.052	0.193
TCGCCG	-19	20	4.171	0.039	8.719	11.653	0.089
GTCGCC	-20	20	3.906	0.035	7.300	7.976	0.049
AACCCT	-21	132	5.329	0.177	14.068	30.478	0.266
CCGTCG	-22	53	3.455	0.052	5.510	10.267	0.071
ACACCC	-22	25	3.206	0.029	5.785	8.350	0.069
ACACTC	-22	68	3.166	0.057	5.562	11.879	0.180
AACCTC	-22	97	3.110	0.069	6.401	15.068	0.214
AACCCC	-26	138	5.219	0.127	6.395	21.226	0.118
ATACCC	-26	101	4.131	0.091	5.570	13.676	0.107
AAACCC	-27	140	5.136	0.159	12.106	30.612	0.420
AAAACC	-28	183	3.401	0.109	8.335	24.647	0.714
AGCCCT	-29	67	3.382	0.092	6.119	7.821	0.063
TAAACC	-30	272	3.743	0.163	8.398	26.301	0.492
CCCTAG	-49	69	5.302	0.140	10.498	16.429	0.073

In this table, classification is done primarily based on peak positions.
If more than one purine residues (A/G) appear in the sequence, it is excluded from Y Patch.
