# Supplementary material for: Identification of plant promoter constituents by analysis of local distribution of short sequences
Source: BMC Genomics. 2007 Mar 8;8:67. doi: 10.1186/1471-2164-8-67 (PMC1832190; doi:10.1186/1471-2164-8-67)
Supplement: Additional file 2 — Characteristics of random distribution (FigS1.pdf). Contains graphs to show relationship between a LDSS parameter and a size of population (Total Area). [file 1471-2164-8-67-S2.pdf]

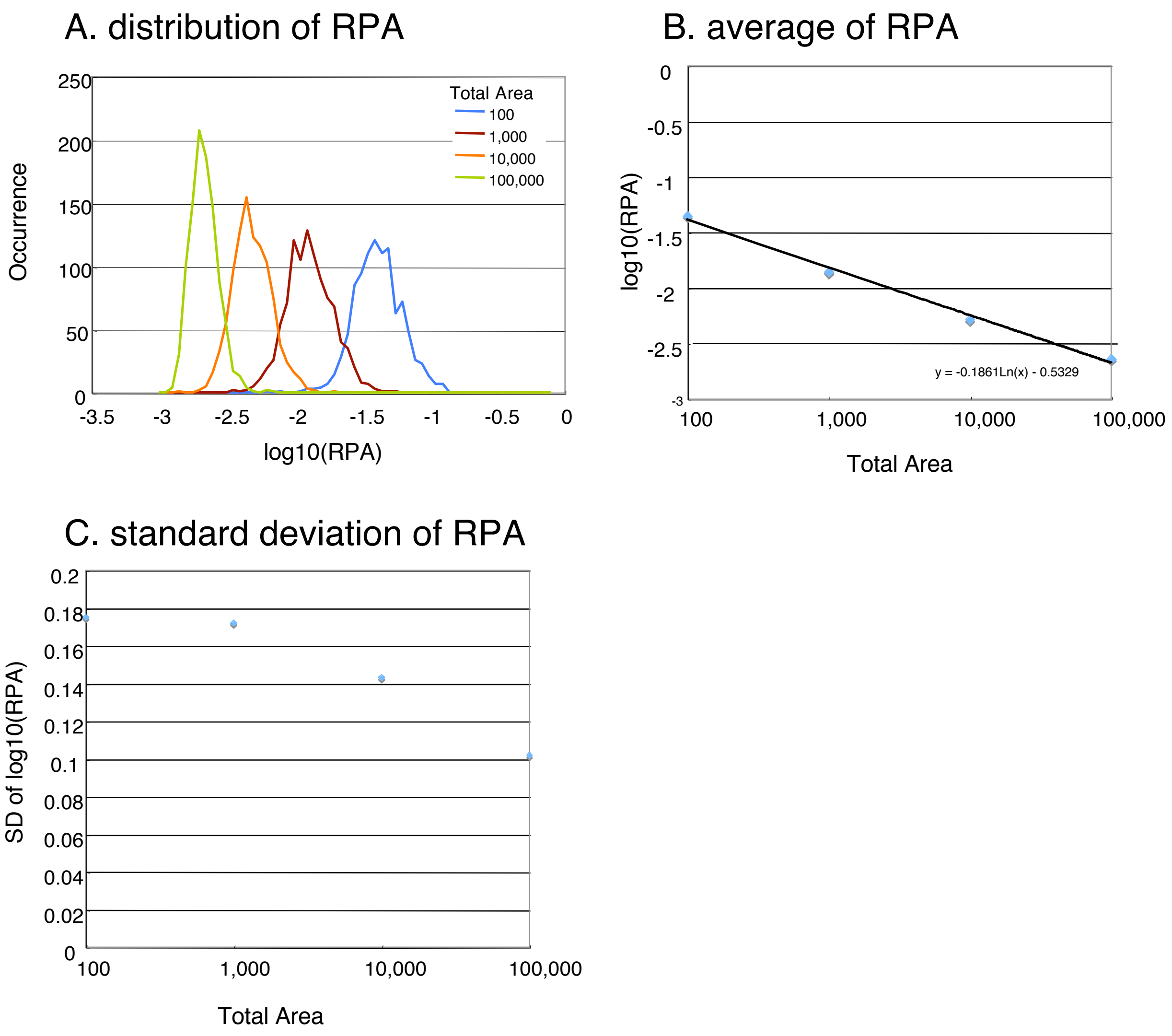

Characteristics of random distribution

Samples of random distribution were generated and subjected to LDSS analysis after smoothing with a bin of 21 bp. This figure represent characteristics of RPA (Relative Peak Area) defined in Figure 3. As shown in panel A, a distribution profile of RPA depends on Total Area, that is total count of the sequence in a promoter database. The results show that rare sequences with small Total Area tend to have big RPA value by chance. When smoothing with a bin of 3 bp, an equation for calculation of average was: Average = -0.1784Ln(Total Area)-0.8026. The standard deviation was 0.13 when Total Area is less than 10,000.

Figure S1
