# Supplementary material for: Identification of plant promoter constituents by analysis of local distribution of short sequences
Source: BMC Genomics. 2007 Mar 8;8:67. doi: 10.1186/1471-2164-8-67 (PMC1832190; doi:10.1186/1471-2164-8-67)
Supplement: Additional file 3 — Arabidopsis core octamers (Table S2.pdf). Contains octamer sequences and parameters. [file 1471-2164-8-67-S3.pdf]

Table S2. Arabidopsis core octamers

Sequence	Peak Position	Peak Width	RPH	RPA	Peak Area/ basal fluctuation	(Peak hight-Base Line)/sd	Occurence/Promoter	p value
TCTCTCTC	-9	159	31.3569	0.4856	14.3745	54.8468	0.2932	0.00E+00
GAGAGAGA*	-9	23	6.2092	0.0496	7.3453	10.8921	0.2054	1.65E-12
TTCTCTCT	-9	86	19.4479	0.2857	11.2244	32.0303	0.1290	0.00E+00
TCTTCTCT	-9	77	18.9255	0.2683	10.9558	32.3933	0.1184	0.00E+00
TTCTTCTC	-9	71	23.1159	0.2389	10.7134	41.5705	0.1123	0.00E+00
TTTCTCTC	-9	87	20.8044	0.2489	7.3563	30.2303	0.1004	0.00E+00
TCTTTCTC	-9	73	16.2630	0.2121	7.8924	26.8879	0.0955	0.00E+00
CTTTCTCT	-9	84	14.9842	0.2498	7.7512	21.9016	0.0893	0.00E+00
CTTCTCTT	-9	55	10.8777	0.1723	7.1370	15.6370	0.0812	0.00E+00
ACACACAC*	-9	26	10.3286	0.0966	7.7453	12.2702	0.0705	5.77E-15
TTCTTCCT	-9	52	15.6098	0.2160	9.6766	20.1014	0.0694	0.00E+00
ATTTCTTC	-9	14	9.1211	0.0447	6.6090	12.6149	0.0687	1.62E-07
CTTCTCTC	-9	82	20.1613	0.3062	9.4200	26.4847	0.0682	0.00E+00
TCATCTTC	-9	39	9.2127	0.1305	6.9331	12.8924	0.0682	0.00E+00
TCTTCCTC	-9	50	17.6471	0.2224	10.2299	25.7709	0.0673	0.00E+00
CTCTTCTC	-9	79	17.9574	0.2898	8.9195	23.0550	0.0668	0.00E+00
TCTTCTCC	-9	42	23.4375	0.1942	10.6041	33.0123	0.0653	0.00E+00
CTTCCTCT	-9	32	10.8565	0.1275	7.1625	12.7910	0.0501	0.00E+00
TCTCCTTC	-9	62	9.7403	0.2056	6.1767	10.6026	0.0500	0.00E+00
CTTCTCCT	-9	52	16.1290	0.2200	7.5427	18.5247	0.0458	0.00E+00
TCTCCTCT	-9	50	16.3265	0.1938	6.2388	17.4007	0.0443	0.00E+00
CTCTCTCC	-9	21	22.6843	0.1645	15.4298	20.9340	0.0433	0.00E+00
TCCTCCTC	-9	24	15.6250	0.1231	9.0666	17.1176	0.0408	3.55E-15
CATTTCTC	-9	21	11.6618	0.0909	6.7355	12.4868	0.0388	1.04E-11
TTCTCCTC	-9	29	17.3193	0.1219	6.5408	18.7001	0.0379	1.03E-14
TTCTCTCC	-9	27	11.8110	0.1289	7.8130	12.4947	0.0373	2.89E-15
TTCCTCTC	-9	26	15.9011	0.1189	6.6221	14.5216	0.0341	5.92E-14
TCTCCTCC	-9	18	11.5044	0.0824	6.8754	10.7663	0.0339	3.06E-10
CTCCTCCT	-9	21	12.5725	0.1110	9.1677	11.7853	0.0332	4.27E-13
CGTCTTCT	-9	41	17.2414	0.2033	7.6578	14.3391	0.0304	0.00E+00
CTCCTCTC	-9	22	20.3349	0.1277	8.5033	17.2420	0.0291	4.97E-14
CTCTCCTC	-9	22	21.8818	0.1504	10.3619	20.3415	0.0289	0.00E+00
TCTCTCCC	-9	27	23.1214	0.1734	10.3531	19.5301	0.0280	0.00E+00
TCCCTCTC	-9	22	26.0870	0.1532	8.7807	20.2966	0.0230	5.66E-15
TCCTCTCC	-9	22	21.3675	0.1333	6.2330	16.4148	0.0196	9.61E-13
TCTCTCGC	-9	7	25.8216	0.0563	8.0364	16.2896	0.0146	8.54E-05
AGAGAGAG*	-10	24	7.8311	0.0579	8.4432	14.7457	0.1997	5.13E-14
TTTCTTCT	-10	54	8.3280	0.1258	8.2540	18.3333	0.1846	0.00E+00
CTCTTTCT	-10	69	13.5135	0.2410	8.6785	19.5749	0.0857	0.00E+00
TTCTCTTC	-10	56	11.6009	0.1787	7.0322	15.7420	0.0840	0.00E+00
CACACACA*	-10	25	8.1433	0.0854	7.4460	10.4814	0.0761	6.08E-14
CTTCTTCC	-10	64	19.7769	0.2564	9.6615	25.9038	0.0690	0.00E+00
TCTCTCCT	-10	23	14.9254	0.1210	9.4177	15.1921	0.0407	5.88E-15
CTCTTCCT	-10	22	9.7276	0.0991	6.9606	10.5105	0.0405	9.17E-13
TCTTTCCT	-10	12	10.2041	0.0472	6.4692	11.4326	0.0390	3.16E-06
CCTCCTCC	-10	23	10.7004	0.0912	6.4242	10.1294	0.0294	1.22E-10
CTCTCTCG	-10	30	19.6970	0.1393	6.4211	15.1240	0.0252	2.55E-14
ACACTCTC	-10	24	12.6582	0.1236	5.9999	10.5966	0.0223	1.65E-12
CTCCCTCT	-10	16	19.5035	0.1224	7.3738	13.8589	0.0162	4.24E-11
CTCCCTCC	-10	13	18.1818	0.0940	6.2401	10.0589	0.0106	2.47E-07
CTTCTTCT	-11	83	15.5562	0.2866	14.6151	36.5892	0.2612	0.00E+00
CTTTCTTC	-11	64	14.6341	0.1957	7.2438	21.0720	0.0778	0.00E+00
TCTCTTCC	-11	56	11.3941	0.1950	6.3970	12.4764	0.0481	0.00E+00
CCTCTCTC	-11	78	23.9085	0.3209	8.9192	23.1997	0.0415	0.00E+00
CTTCTTCG	-11	44	10.1404	0.1681	6.0317	10.4021	0.0381	0.00E+00
TCACTCTC	-11	24	12.9450	0.1091	6.9602	12.8751	0.0373	1.99E-13
CCTTCTCT	-11	25	14.5548	0.1141	7.9812	15.9460	0.0349	1.31E-13
CTCTCATT	-11	20	10.6195	0.0909	7.1601	10.4246	0.0335	4.06E-11
GTCTCTTC	-11	20	11.7878	0.0924	7.1754	11.3784	0.0286	1.20E-10
CTCCTTTC	-11	19	14.2518	0.1086	7.3457	12.6780	0.0239	1.68E-11

CGTCTTCC	-11	16	16.6667	0.1159	6.7760	10.5044	0.0134	7.26E-10
CCCTTCCC	-11	6	30.8642	0.0860	10.1195	12.0921	0.0051	9.68E-05
TCTTCTTC	-12	80	18.3464	0.3079	18.5013	45.4515	0.3062	0.00E+00
CTTCTTCA	-12	45	8.9523	0.1390	7.7626	13.7426	0.0860	0.00E+00
CAGAGAGA*	-12	20	8.3945	0.0773	7.8111	11.0994	0.0526	2.47E-11
CCTTCTTC	-12	63	9.5613	0.1963	6.0657	11.8864	0.0526	0.00E+00
CTCTCTCA	-12	70	15.5096	0.2390	6.5890	15.7506	0.0516	0.00E+00
TCTCTCAT	-12	23	10.1215	0.0943	7.4139	12.0803	0.0444	1.21E-12
TCGTCTTC	-12	63	17.5439	0.2689	8.3505	18.4363	0.0408	0.00E+00
TCCTTCTC	-12	24	11.5562	0.1117	7.9373	13.2877	0.0375	1.06E-13
TTCCTCCT	-12	23	13.1086	0.1078	7.5409	13.3865	0.0316	1.40E-12
CTTCACTC	-12	27	18.3486	0.1376	6.4360	14.9570	0.0214	1.86E-13
TTCTTCTT	-13	67	11.0387	0.2205	15.4474	29.7726	0.3174	0.00E+00
TCTCTCTT	-13	78	15.3302	0.2831	12.9989	26.9014	0.1352	0.00E+00
CTCTTCTT	-13	89	15.3641	0.2372	7.6245	24.8463	0.1013	0.00E+00
TCTCTTTC	-13	101	14.7679	0.2597	7.0536	22.7837	0.0965	0.00E+00
TCTCTCAC	-13	30	15.2284	0.1344	9.0760	15.7851	0.0438	0.00E+00
TTTCCTCC	-13	11	11.4679	0.0510	6.2913	10.1220	0.0249	1.58E-05
CCTCTCCT	-13	21	18.2927	0.1772	8.8646	12.5600	0.0164	4.88E-15
CCCTCTCC	-13	19	24.4755	0.1473	6.1307	12.2416	0.0090	1.42E-10
TCCGTCTC	-13	9	20.8333	0.0816	6.9410	10.6878	0.0086	9.50E-06
CCCGTCTT	-13	9	26.6667	0.1088	7.0744	10.2977	0.0039	1.59E-05
TCTCTTCT	-14	55	16.6558	0.2409	13.0227	26.3221	0.1127	0.00E+00
CTCTCTTC	-14	81	21.5405	0.3499	11.2953	26.4464	0.0677	0.00E+00
TTCCTTCT	-14	19	8.6486	0.0695	7.1095	11.3928	0.0503	3.72E-10
ACTCTCTC	-14	26	14.5161	0.1445	10.9081	14.7393	0.0464	0.00E+00
CTCTCTCT	-15	158	30.2498	0.4817	14.5979	53.5672	0.2813	0.00E+00
CTTCCTTC	-15	19	10.3627	0.0989	7.8267	10.0868	0.0338	5.57E-12
TCTCTCGT	-15	19	16.5017	0.1075	7.0137	12.3357	0.0212	6.45E-11
CTCTCTTT	-16	22	11.6732	0.0968	12.6920	17.9047	0.1072	0.00E+00
CGTCTCTC	-16	24	25.5102	0.1649	8.8456	15.2850	0.0180	1.11E-14
ATCTCTCT	-17	69	12.8952	0.2561	9.4229	16.8899	0.0892	0.00E+00
GTCTCTCT	-17	68	15.2395	0.2363	7.2314	16.5251	0.0522	0.00E+00
TCCTCTCT	-17	71	12.7186	0.2492	6.8905	13.0404	0.0446	0.00E+00
CATCTCTC	-17	27	14.9007	0.1384	8.3661	14.8000	0.0386	0.00E+00
CCTCTTCT	-17	19	19.5896	0.1277	12.1405	18.1221	0.0376	3.33E-15
CCTCTCTT	-17	33	15.0376	0.1337	7.4637	15.2613	0.0350	2.11E-15
CCTCTTTC	-17	17	17.9063	0.0962	6.9263	14.1124	0.0229	3.51E-10
GTCGTCTC	-17	11	21.5054	0.0888	7.9499	11.6261	0.0136	1.10E-07
CTCCCACT	-17	16	21.7391	0.1252	6.7795	10.7335	0.0096	2.49E-09
CATCTTCT	-18	50	7.8631	0.1565	6.3320	10.1716	0.0612	0.00E+00
CACTCTCT	-18	23	14.0105	0.1262	9.1637	13.5621	0.0387	3.33E-15
CTCTCTGT	-18	25	12.3106	0.1171	8.1900	11.2905	0.0360	5.04E-14
CTTTCTCC	-18	15	11.8671	0.0717	7.9100	13.1177	0.0352	3.92E-09
TTCCCTCT	-18	23	16.5816	0.1128	7.0485	13.7014	0.0265	2.71E-12
CGTCGTCT	-18	19	16.0643	0.1077	6.3179	10.7504	0.0176	3.26E-10
CCCTCTTC	-18	19	26.1628	0.1614	8.0823	13.9972	0.0124	9.26E-13
GCCGTCGT	-18	11	26.3158	0.0830	7.0337	10.6940	0.0077	1.46E-05
TGCCTCCC	-18	10	33.3333	0.1356	8.4531	10.1582	0.0027	6.38E-06
GCGCCGCC	-18	6	39.2157	0.1186	10.1516	12.5947	0.0025	7.00E-05
TCTCTCTG	-19	55	11.7801	0.1711	6.0040	13.6317	0.0508	0.00E+00
TATCTCTC	-19	57	12.9310	0.2114	7.0435	12.0051	0.0436	0.00E+00
CTTCGTCT	-19	46	22.9226	0.2746	8.7881	18.5543	0.0266	0.00E+00
CCTCCTCT	-19	25	14.7493	0.1332	6.3360	10.9575	0.0222	2.81E-13
CTTCCCTC	-19	25	25.5319	0.1745	8.1721	17.4222	0.0179	2.78E-15
CTCTCCCG	-19	9	27.7778	0.0938	8.6803	10.6324	0.0054	2.07E-05
CCCTAGTC	-19	5	52.0833	0.1030	16.3217	15.5874	0.0036	5.60E-05
TCTTCGTC	-20	42	16.1597	0.2148	8.4746	16.0505	0.0320	0.00E+00
CTCCTCTT	-20	20	11.5453	0.1021	7.4294	10.9473	0.0319	4.67E-12
TCTCACTC	-20	26	12.0614	0.1036	6.1409	10.9942	0.0282	1.09E-11
CCTCCCTC	-20	18	21.2766	0.1534	6.7110	11.6973	0.0085	9.49E-11
CGTCTCCG	-20	10	21.5517	0.1085	7.5297	10.6998	0.0066	6.10E-07
AACCTCCG	-20	10	25.0000	0.0976	6.3867	11.7851	0.0065	3.71E-06
CACACCCC	-20	12	39.6825	0.1704	8.3364	14.4133	0.0034	2.46E-08

CTCTGCGC	-20	10	31.7460	0.1431	7.3960	10.7287	0.0030	1.26E-06
TTCTTCGT	-21	31	11.6861	0.1353	7.1900	11.6613	0.0376	0.00E+00
CCTTCCTC	-22	23	20.3390	0.1267	6.2084	14.4541	0.0196	3.22E-12
ATCGCCGT	-22	9	26.3158	0.0817	6.7382	11.3926	0.0061	7.29E-05
CCCTCTCT	-24	21	22.1088	0.1640	10.0889	15.0560	0.0230	8.88E-16
CGCTCTCT	-24	18	20.3488	0.1316	6.5679	11.0567	0.0115	1.87E-10
CGCTTCCC	-25	13	40.9836	0.1318	7.8318	13.8883	0.0044	3.80E-07
CGTTCGTG	-25	8	28.9855	0.1014	7.1530	11.0028	0.0035	8.06E-05
AACCTCTC	-26	25	19.7368	0.1487	6.9016	15.0695	0.0200	5.22E-14
CCCCTCTC	-26	18	26.3158	0.1701	7.9905	12.4065	0.0080	1.74E-11
AAACAGCG	-26	9	21.2766	0.0807	6.1781	10.3864	0.0078	2.07E-05
AGGGCACA	-26	7	35.0877	0.1184	9.8183	11.7584	0.0031	2.01E-05
AACCCTAA	-27	60	8.9514	0.1941	7.1516	11.8759	0.0722	0.00E+00
AAACCTCT	-27	21	12.4334	0.0743	6.0731	12.9513	0.0334	2.96E-09
AAACCCTA	-28	69	11.2812	0.2341	9.3047	17.6180	0.0852	0.00E+00
AAACCCTT	-28	26	12.6476	0.0940	6.3349	13.2584	0.0378	6.22E-12
TAAACCCA	-28	12	11.3636	0.0512	7.4274	11.2356	0.0368	1.24E-06
AACCCCTC	-28	8	26.0417	0.0948	8.6102	10.5536	0.0061	8.59E-06
AAAACCCT	-29	60	19.7152	0.2347	9.4464	26.4680	0.0665	0.00E+00
AATCTCTC	-29	26	9.9778	0.0978	7.6746	12.2603	0.0574	3.72E-14
ATAAACCC	-29	18	26.7176	0.1367	13.5573	26.3302	0.0358	8.88E-16
TAAATCCC	-29	9	24.4361	0.0720	8.1989	17.7398	0.0165	1.03E-06
AAATACGC	-29	9	31.0078	0.0824	7.5812	15.3030	0.0086	8.20E-06
ATAAAATC	-30	15	10.8853	0.0424	7.2643	16.5867	0.0896	5.76E-08
ATATACAC	-30	17	14.8280	0.0787	8.2671	18.0153	0.0495	2.85E-11
TATAAACC	-30	22	35.7686	0.1974	17.0395	40.9960	0.0451	0.00E+00
TAAAACCC	-30	28	29.7521	0.1319	8.9115	31.0872	0.0449	0.00E+00
TAAACCCT	-30	37	15.1976	0.1508	7.3419	16.9815	0.0429	0.00E+00
ATATAGAG	-30	11	13.6571	0.0610	7.3049	14.2025	0.0335	1.19E-07
ATAAATCC	-30	15	20.8333	0.0817	8.3649	19.4866	0.0334	4.17E-10
TATATAGA	-31	12	11.6518	0.0616	10.3421	17.0433	0.0748	1.51E-10
TATATAGT	-31	12	9.5498	0.0459	7.5295	13.6390	0.0739	5.78E-08
ATATATTC	-31	17	8.2897	0.0539	6.3074	11.4026	0.0626	1.02E-08
ATAAAACC	-31	18	18.4758	0.0988	11.0191	21.9821	0.0599	1.85E-14
ATATAAGA	-31	16	10.7481	0.0520	6.3140	16.0501	0.0578	3.74E-08
TAAATAGA	-31	17	15.0966	0.0862	9.1399	16.3854	0.0546	1.38E-12
ATAAACAC	-31	17	18.8067	0.0917	9.6790	22.8252	0.0454	1.91E-12
ATAAAAGC	-31	19	15.8479	0.0814	7.6913	17.1935	0.0417	6.35E-11
ATAAAGAG	-31	13	11.8881	0.0634	8.1165	13.9173	0.0412	1.24E-08
ATAAACTC	-31	17	12.9573	0.0696	6.2434	13.7526	0.0372	4.48E-09
ATAAAGAC	-31	8	15.3846	0.0513	9.2186	14.9718	0.0315	3.46E-06
ATAAAGCA	-31	12	11.9454	0.0640	7.6629	12.3405	0.0313	8.36E-08
AAATACTC	-31	14	15.7993	0.0653	6.4723	14.9109	0.0303	7.47E-08
TATATACC	-31	12	15.6863	0.0983	9.7249	12.7967	0.0272	4.92E-11
TAAATACG	-31	17	35.2349	0.1408	9.5905	26.9330	0.0195	2.63E-13
AAATACCC	-31	16	26.0417	0.1060	7.5899	20.2927	0.0189	2.44E-10
TAAACCCC	-31	17	19.4346	0.1196	7.2583	15.2101	0.0167	5.46E-11
TAAATAAA	-32	15	5.8807	0.0268	7.6223	13.7203	0.2293	2.13E-07
AATAAATA	-32	18	5.5394	0.0269	6.1213	12.1087	0.2226	2.51E-07
ATAAATAA	-32	21	11.1283	0.0521	11.0563	27.5628	0.2166	3.09E-13
TATATATT	-32	15	6.8326	0.0452	11.3007	17.8753	0.2095	1.20E-11
TAAATATA	-32	14	7.2266	0.0378	7.4513	13.5929	0.1350	2.15E-08
TAAAAAGA	-32	13	7.4169	0.0368	7.4739	11.0665	0.1197	8.80E-08
ATAAAAAG	-32	24	12.5142	0.0733	8.7762	20.8968	0.1161	3.61E-14
ATAAAAAC	-32	22	11.4334	0.0558	7.4540	20.1562	0.1122	3.45E-11
TATATACA	-32	15	14.1243	0.0723	12.8698	26.8533	0.1046	1.53E-13
TATAAACA	-32	18	24.1996	0.1119	13.5304	37.1799	0.0777	0.00E+00
ATAAATAC	-32	23	53.7408	0.2147	22.0721	66.1178	0.0733	0.00E+00
TAAATACA	-32	17	17.0507	0.0900	13.4708	25.8125	0.0707	3.50E-14
ATATATCT	-32	18	9.0386	0.0568	6.7305	13.2214	0.0672	2.05E-09
TATAAAGA	-32	22	21.1786	0.1223	11.8366	30.6669	0.0634	0.00E+00
TATATAAG	-32	17	20.2874	0.1169	13.0332	28.8786	0.0625	0.00E+00
TATAAACT	-32	17	9.4851	0.0582	6.8342	12.8284	0.0616	2.55E-09
TAAATATC	-32	17	14.7493	0.0691	8.3620	19.7116	0.0590	1.03E-10

ATTTAAAG	-32	12	9.0000	0.0374	6.2428	11.1474	0.0571	1.00E-05
TAAATACT	-32	22	19.9035	0.0799	6.6014	24.2359	0.0498	1.92E-11
AAATACAC	-32	17	13.2509	0.0600	6.2944	13.8640	0.0368	8.20E-08
ATAAAAGG	-32	21	17.7066	0.0872	6.1552	17.4725	0.0352	6.67E-11
TAAATACC	-32	17	48.7805	0.2005	16.0202	37.0658	0.0248	0.00E+00
TATAAAGC	-32	23	25.3333	0.1457	8.6230	21.6753	0.0245	1.05E-14
TATAAAGG	-32	19	27.5229	0.1324	8.9755	22.6314	0.0221	3.48E-13
TAAATAGC	-32	22	20.1729	0.1360	7.0936	16.2595	0.0210	3.03E-13
ATATATAT	-33	19	7.1050	0.0483	14.1707	27.8980	0.6212	0.00E+00
ATATATAA	-33	21	14.9313	0.0899	16.6656	41.2068	0.2079	0.00E+00
ATATAAAA	-33	19	11.0812	0.0567	10.7085	26.0093	0.1983	9.07E-14
TATAAAAA	-33	25	11.8639	0.0648	9.4748	27.3855	0.1980	3.00E-15
ATAAATAT	-33	19	14.3242	0.0680	12.1796	33.4569	0.1693	4.55E-15
ATATAAAT	-33	20	18.1530	0.1017	18.7175	44.7924	0.1674	0.00E+00
TTATAAAA	-33	13	9.1925	0.0404	10.0962	19.0594	0.1651	1.17E-09
TATAAAAT	-33	11	7.6160	0.0344	9.2573	14.7684	0.1577	3.78E-08
TATTTAAA	-33	16	8.1058	0.0369	6.8702	15.7674	0.1331	3.71E-08
TATATATG	-33	13	6.3238	0.0353	7.0561	11.8174	0.1170	2.20E-07
ATATATAC	-33	13	19.4458	0.1053	21.0327	36.6057	0.1109	0.00E+00
ATATATAG	-33	18	13.9824	0.0835	10.5910	24.9352	0.0951	1.04E-14
ATAAAAGA	-33	14	8.7660	0.0387	7.6664	14.4587	0.0908	2.72E-07
ATATAAAC	-33	18	41.0477	0.1960	30.3057	67.0933	0.0889	0.00E+00
TATATATC	-33	17	12.8968	0.0813	11.0401	20.4541	0.0832	8.52E-14
TATAAAAC	-33	23	23.2650	0.1126	11.6595	36.3149	0.0813	0.00E+00
ATATAAAG	-33	22	29.6571	0.1475	15.2361	43.2862	0.0678	0.00E+00
TATATAAC	-33	14	17.9153	0.0966	12.9920	26.0201	0.0641	1.60E-14
ATAAATAG	-33	22	21.4505	0.1415	13.9351	27.2923	0.0629	0.00E+00
TATAAAAG	-33	18	22.5933	0.1153	14.2754	31.4139	0.0628	0.00E+00
ATAAATTG	-33	12	9.5602	0.0408	7.1724	13.0216	0.0605	1.81E-06
TATAAATC	-33	19	14.8148	0.0794	8.5827	20.9619	0.0603	3.65E-12
TATAAATG	-33	19	12.3574	0.0682	7.5785	16.8081	0.0596	1.24E-10
GTATTTAT	-33	10	8.1262	0.0342	6.5484	10.4284	0.0558	4.30E-05
TTATAAAG	-33	19	10.5152	0.0592	6.2337	13.6891	0.0543	5.10E-09
ATATAATG	-33	10	9.3458	0.0390	6.9014	12.1299	0.0479	1.55E-05
ATAAAACG	-33	10	15.3722	0.0567	8.7788	15.6837	0.0375	1.95E-07
ATATAAGC	-33	10	23.0415	0.0945	11.7831	19.6865	0.0254	2.08E-10
TATATATA	-34	20	13.2729	0.0829	24.5214	58.0240	0.6283	0.00E+00
TATATAAA	-34	25	36.4391	0.1755	27.0710	89.1592	0.2155	0.00E+00
TATAAATA	-34	19	43.2655	0.2076	37.3246	92.0622	0.1727	0.00E+00
TATATAAT	-34	18	11.7202	0.0570	9.2380	23.9878	0.1458	1.77E-12
TATAAATT	-34	20	11.8110	0.0627	9.2371	23.2545	0.1369	3.30E-13
TTATAAAT	-34	18	16.1080	0.0804	12.9000	31.5650	0.1348	0.00E+00
CTATAAAT	-34	20	57.9577	0.2685	34.6021	85.3682	0.0762	0.00E+00
CATAAATA	-34	19	9.8361	0.0504	6.0590	14.1212	0.0718	1.25E-08
GATATAAA	-34	11	10.3383	0.0490	9.3254	14.6526	0.0565	1.31E-07
CTATTTAA	-34	12	18.1113	0.0885	11.5176	20.6372	0.0443	5.52E-12
TATTTAAG	-34	11	10.2433	0.0522	7.5643	11.9610	0.0414	4.11E-07
TCTATAAG	-34	13	15.6250	0.0783	6.2056	12.1694	0.0180	1.34E-07
AATATATA	-35	17	5.9194	0.0304	6.0166	13.0270	0.2047	5.06E-08
TTATATAT	-35	18	9.7062	0.0621	11.6392	23.4577	0.1993	8.55E-15
TTTATAAA	-35	14	10.9280	0.0557	14.5383	26.2015	0.1728	5.81E-13
AATATAAA	-35	22	9.8829	0.0530	8.1608	20.9264	0.1670	2.64E-12
ATTATATA	-35	16	8.1211	0.0425	8.0124	17.3851	0.1462	1.13E-09
TTATATAA	-35	16	17.3069	0.0834	16.6560	37.3202	0.1393	0.00E+00
CTATATAT	-35	20	32.2165	0.1802	26.9070	68.3533	0.1092	0.00E+00
GTATATAT	-35	16	16.7736	0.0901	14.8517	32.2198	0.1077	0.00E+00
TCTATAAA	-35	22	43.0061	0.2162	22.1770	60.6752	0.0754	0.00E+00
CTATATAA	-35	22	71.7073	0.2918	33.8490	101.8970	0.0747	0.00E+00
CTATAAAA	-35	20	38.8437	0.1787	20.7856	56.5839	0.0715	0.00E+00
GTATATAA	-35	20	19.8939	0.1081	11.1128	28.1932	0.0656	0.00E+00
CTTTATAA	-35	17	21.9207	0.1034	12.2561	28.9222	0.0576	8.55E-15
CTATTTAT	-35	16	13.4486	0.0700	8.4340	17.8026	0.0554	1.35E-10
GTATAAAT	-35	16	23.2955	0.1010	11.8445	30.9291	0.0509	5.70E-14
ATCTATAA	-35	13	10.0111	0.0547	7.5459	12.3660	0.0488	5.39E-08

CCATATAT	-35	12	13.0024	0.0556	8.4608	16.1342	0.0485	4.14E-08
GCTATAAA	-35	15	32.5630	0.1578	14.3891	27.4288	0.0270	0.00E+00
CTATAAAG	-35	20	17.8147	0.1115	6.6674	15.3138	0.0231	1.27E-11
TGCTATAA	-35	10	15.4062	0.0576	6.1806	12.2451	0.0196	1.14E-05
CCCTTAAA	-35	10	27.4510	0.0684	8.1379	18.5834	0.0195	7.73E-07
CCTATTTA	-35	18	21.4067	0.1074	6.4656	16.4327	0.0195	1.40E-10
CCGTATAA	-35	15	23.8095	0.1177	7.5507	10.8671	0.0085	2.16E-08
TTTATATA	-36	15	11.9263	0.0599	13.7085	28.1300	0.1832	5.15E-14
CATATATA	-36	18	11.7866	0.0644	10.0964	24.6285	0.1260	4.03E-13
TGTATATA	-36	22	19.5313	0.0971	11.4039	35.9493	0.1067	0.00E+00
TCTATATA	-36	21	48.3532	0.2290	27.0727	77.1215	0.0916	0.00E+00
CATATAAA	-36	19	14.1034	0.0738	9.6383	23.6651	0.0832	9.15E-13
ACTATATA	-36	19	16.8303	0.1005	11.8737	27.0601	0.0781	0.00E+00
AGTATATA	-36	21	10.9971	0.0615	6.4859	18.9730	0.0686	3.38E-10
TGTATAAA	-36	15	12.3457	0.0650	9.5216	18.3984	0.0650	1.66E-10
ACTATAAA	-36	16	22.2841	0.1162	13.9619	29.7737	0.0619	0.00E+00
CTTATAAA	-36	17	19.3717	0.0886	10.7492	25.1040	0.0612	2.29E-13
CTTATATA	-36	21	18.0723	0.1198	11.5206	25.7119	0.0603	0.00E+00
TTCTATAA	-36	21	20.8111	0.1056	10.3848	28.0036	0.0584	4.22E-15
CAATATAA	-36	14	9.2593	0.0433	6.5024	12.2918	0.0573	1.01E-06
TCTATTTA	-36	16	14.5540	0.0853	10.6901	20.3475	0.0558	1.44E-12
GTTATAAA	-36	14	12.7551	0.0723	10.3590	17.2018	0.0550	7.12E-11
CATTATAA	-36	11	9.4340	0.0378	6.0790	11.9628	0.0477	2.52E-05
CTCTATTT	-36	15	11.4320	0.0575	6.8894	14.3753	0.0466	3.02E-08
TCCATATA	-36	11	9.0144	0.0541	7.7692	10.5619	0.0427	1.76E-07
GTGTATAT	-36	19	21.0312	0.0902	7.9213	25.7385	0.0416	6.42E-12
CCTATATA	-36	19	53.2646	0.2653	22.3168	56.6952	0.0352	0.00E+00
CTCTATAA	-36	22	42.9616	0.2134	15.7726	43.7027	0.0347	0.00E+00
CCTATAAA	-36	23	66.2252	0.2858	20.4887	59.7207	0.0335	0.00E+00
TCCTTTAT	-36	20	15.8287	0.1006	7.8517	15.1386	0.0334	4.20E-12
GCTATATA	-36	20	28.2486	0.1497	10.6282	28.3728	0.0285	9.99E-16
CCTTTATA	-36	19	31.4010	0.1569	11.6546	26.0143	0.0284	0.00E+00
CCTTATAA	-36	17	22.0386	0.1010	7.5890	18.8548	0.0229	1.29E-10
GGCTATAA	-36	15	30.1205	0.1199	7.4532	19.8255	0.0151	1.21E-10
CCCTATAA	-36	20	49.4792	0.2400	12.0425	32.4455	0.0133	0.00E+00
CGCTATAA	-36	12	31.4961	0.1511	7.3871	14.9065	0.0061	2.29E-09
AGCGTATA	-36	13	26.6667	0.1285	7.3343	10.0145	0.0050	2.25E-07
TCATATAT	-37	15	6.9444	0.0415	6.2391	11.3321	0.0862	1.13E-07
TTGTATAT	-37	12	10.9489	0.0468	8.2315	16.9219	0.0758	3.33E-08
CTTTATAT	-37	19	18.6069	0.1025	11.5173	25.8452	0.0633	3.89E-15
TCTTTATA	-37	14	21.1665	0.0966	14.7390	31.2202	0.0624	2.11E-14
TACTATAA	-37	16	10.0000	0.0592	6.6804	12.4385	0.0464	1.78E-08
CTCTATAT	-37	21	54.1455	0.2308	21.2330	59.3809	0.0427	0.00E+00
GTCTATAT	-37	23	30.3347	0.1321	8.4960	29.2394	0.0273	4.23E-14
TCCTATAT	-37	16	31.5315	0.1354	12.1892	28.4953	0.0269	2.54E-14
CATCTATA	-37	14	14.0713	0.0789	6.7475	13.1436	0.0265	6.05E-09
ACTCTATA	-37	20	20.9205	0.0934	6.5498	20.3030	0.0261	2.10E-10
TGTCTATA	-37	14	22.8758	0.1173	10.7702	20.6127	0.0255	1.55E-12
TGCTATAT	-37	11	12.4283	0.0597	7.1297	12.0394	0.0248	1.44E-06
TTGCTATA	-37	12	15.8562	0.0667	7.2829	13.7999	0.0246	2.37E-07
TTCCTATA	-37	14	18.1406	0.0867	7.5282	15.6435	0.0239	2.08E-09
TCCTATAA	-37	22	24.4565	0.1366	7.4129	20.3494	0.0224	1.40E-13
CACTATAA	-37	16	20.5656	0.1102	7.9851	16.2039	0.0223	2.31E-11
CCTTATAT	-37	20	23.5988	0.1269	7.0848	18.5851	0.0198	2.86E-12
CTATAAGT	-37	14	18.1452	0.1099	7.4676	12.1366	0.0152	7.28E-10
GCCTATAA	-37	15	49.1803	0.1881	13.3659	27.5780	0.0144	3.89E-15
TCCCTATA	-37	17	28.5171	0.1798	9.1879	19.5806	0.0142	1.54E-14
CCCTATAT	-37	17	39.5833	0.1834	9.3083	26.2281	0.0136	1.44E-14
GCCTATAT	-37	19	33.3333	0.1739	8.0480	21.6792	0.0126	1.24E-13
CCTTCTAT	-37	17	22.2222	0.1319	6.6189	14.7300	0.0126	8.13E-11
AAGGCTAT	-37	7	20.9302	0.0671	6.5129	12.0464	0.0103	5.40E-05
CTCCCTTA	-37	13	27.3438	0.1111	6.2117	12.9547	0.0076	1.48E-07
AAGCGTAT	-37	13	29.4118	0.0882	6.5724	12.1737	0.0072	8.82E-06
CTCCCTAT	-37	7	35.7143	0.1170	11.2330	17.5313	0.0069	1.20E-07

CCCCTATA	-37	12	86.6667	0.2453	17.6729	35.4002	0.0058	6.57E-14
ACCCCTAT	-37	8	30.3030	0.0869	9.0141	10.8612	0.0050	9.18E-05
CCCCTTAT	-37	7	41.6667	0.0923	11.3466	14.4643	0.0048	5.31E-05
CGCCTATA	-37	19	62.5000	0.2715	12.8567	20.0213	0.0041	1.83E-13
TTTCTATA	-38	14	11.3556	0.0604	9.9593	18.1467	0.0765	1.86E-10
TTCTATAT	-38	15	16.1421	0.0930	12.4261	24.3925	0.0664	2.95E-14
TCTCTATA	-38	21	44.1640	0.2183	20.5574	49.1775	0.0477	0.00E+00
TGTGTATA	-38	18	17.3238	0.0726	6.1132	20.9142	0.0425	6.32E-10
CTTCTATA	-38	21	23.5849	0.1593	11.1382	23.7223	0.0302	0.00E+00
CTCTTATA	-38	21	12.4224	0.0898	6.5387	11.9361	0.0277	2.90E-10
CACTATAT	-38	14	25.3700	0.1328	12.6640	24.2352	0.0248	1.01E-13
GTCTATAA	-38	14	22.4551	0.1084	7.9006	17.8169	0.0184	1.85E-10
CCTCTATA	-38	20	49.3827	0.2259	11.1418	32.1515	0.0160	0.00E+00
CTCCTATA	-38	18	36.0360	0.2019	10.6303	23.8981	0.0139	8.88E-16
TGCCTATA	-38	18	51.2048	0.2407	11.0189	28.6506	0.0103	0.00E+00
CCCTTATA	-38	15	24.5399	0.1184	6.0625	13.8199	0.0094	8.92E-09
GCACTATA	-38	9	29.6296	0.1221	8.8321	14.1675	0.0071	4.44E-08
CGCTTATA	-38	14	31.2500	0.1247	6.7523	13.5583	0.0063	7.58E-08
TCCCCTAT	-38	11	69.6203	0.1881	13.4767	29.2589	0.0055	5.63E-11
ACGCCTAT	-38	11	51.2821	0.1460	12.5016	14.6103	0.0036	2.88E-07
TTCTCTAT	-39	26	17.7515	0.1068	7.9216	21.7816	0.0537	7.66E-15
CTCTCTAT	-39	49	26.2346	0.2034	8.0104	28.3042	0.0473	0.00E+00
CTCTCTTA	-39	11	11.8110	0.0456	7.1298	11.7525	0.0315	1.99E-05
CTACTATA	-39	13	13.5659	0.0721	7.4800	12.5891	0.0263	3.53E-08
CCACTATA	-39	16	24.9169	0.1348	8.5632	19.5087	0.0171	2.85E-12
CGTCTATA	-39	9	24.2424	0.0783	6.1551	13.1972	0.0089	1.47E-05
GGGCTATA	-39	10	28.5714	0.0912	7.1875	12.7472	0.0070	6.37E-06
CCCTCTAT	-39	15	34.1880	0.1758	7.2548	15.3562	0.0061	1.03E-10
CTCCCCTA	-39	9	37.8788	0.1001	7.9683	13.8903	0.0043	3.06E-05
CTGCCTAT	-39	8	43.4783	0.1659	11.6509	15.4885	0.0036	2.87E-08
CGTCCTAT	-39	6	39.6825	0.1187	10.5365	14.1671	0.0031	1.92E-05
CCTCCTTC	-47	15	17.1103	0.1018	6.5403	11.3397	0.0162	2.08E-09
CTCCTTCT	-48	29	13.1579	0.1135	6.7902	14.2028	0.0380	6.21E-14
CTACACGC	-50	13	30.3030	0.1190	6.2559	10.0930	0.0042	2.84E-06
