# Supplementary material for: Identification of plant promoter constituents by analysis of local distribution of short sequences
Source: BMC Genomics. 2007 Mar 8;8:67. doi: 10.1186/1471-2164-8-67 (PMC1832190; doi:10.1186/1471-2164-8-67)
Supplement: Additional file 4 — Rice core octamers (Table S3.pdf). Contains octamer sequences and parameters. [file 1471-2164-8-67-S4.pdf]

Table S3. Rice core octamers

Sequence	Peak Position	Peak Width	RPH	RPA	Peak Area/ basal fluctuation	(Peak hight-Base Line)/sd	Occurence/Promoter	p value
TCCTCCTC	-9	79	13.4497	0.2587	11.5032	26.1291	0.1743	0.00E+00
CTCTCCTC	-9	97	15.8198	0.3319	10.6518	22.3193	0.0989	0.00E+00
CCTCCTCT	-9	63	14.2405	0.2497	10.1762	18.7701	0.0798	0.00E+00
TCTCCTCT	-9	107	13.2597	0.3003	6.5565	15.8104	0.0775	0.00E+00
CCCTCCCC	-9	66	12.4729	0.2156	7.2995	14.9758	0.0759	0.00E+00
CCTCCCCC	-9	73	17.6471	0.2261	7.0388	21.6199	0.0721	0.00E+00
TCCTCCCC	-9	28	17.3333	0.1396	10.4616	19.2179	0.0662	0.00E+00
TCTCTCCC	-9	70	14.4092	0.2396	6.9602	16.3740	0.0575	0.00E+00
TTCTCCTC	-9	50	10.0629	0.1604	6.1865	11.6844	0.0571	0.00E+00
CTCCTCTT	-9	19	11.1111	0.0823	8.2441	13.0750	0.0556	1.20E-11
CTCTCCCC	-9	27	19.1304	0.1482	10.9389	20.1599	0.0490	0.00E+00
CCACCCCC	-9	58	15.2284	0.1898	6.6217	12.1989	0.0436	0.00E+00
TCCCCTCT	-9	24	16.1002	0.1228	9.2787	16.4738	0.0416	1.28E-14
TCTCCCCC	-9	27	19.6759	0.1289	7.9714	16.1725	0.0386	7.99E-15
TCTTCCCC	-9	55	14.2566	0.2072	6.6767	14.2776	0.0368	0.00E+00
CCAAACCC	-9	83	22.3368	0.2959	6.0375	16.5884	0.0324	0.00E+00
CCTTCCCT	-9	18	18.8406	0.0968	7.4675	14.5547	0.0289	1.24E-10
CGCTCCTC	-9	19	15.0273	0.0924	6.5305	12.5866	0.0270	6.16E-10
GTCTCCTC	-9	31	15.7143	0.1513	6.3252	12.6466	0.0262	7.66E-15
CCTCTCGC	-9	24	20.7469	0.1275	7.1934	13.4784	0.0245	1.14E-12
CGTCTTCT	-9	15	19.0678	0.0827	6.3691	13.4500	0.0195	7.30E-08
TCCTCGAG	-9	4	45.4545	0.0713	14.6389	29.2345	0.0145	6.72E-06
CTCTCTCC	-10	87	13.7043	0.2404	7.2802	20.7295	0.0950	0.00E+00
CTTCCTCC	-10	50	10.8601	0.1623	7.1376	15.1682	0.0804	0.00E+00
CCCCTCCC	-10	86	12.1669	0.2560	7.1959	14.4040	0.0785	0.00E+00
TCTCTTTC	-10	9	9.9338	0.0388	7.1536	11.1460	0.0501	2.79E-05
CGCCTCCT	-10	21	11.9658	0.0866	7.3600	12.3729	0.0445	2.95E-11
CTCGCTTC	-10	17	18.9189	0.1206	6.7595	11.4077	0.0143	5.62E-10
GCGCTCTC	-10	11	22.4719	0.0828	6.7066	12.1771	0.0135	9.83E-07
TCTTCCTC	-11	68	12.0424	0.2010	7.1421	17.4913	0.0751	0.00E+00
CCTTCCTC	-11	55	16.2037	0.2190	6.8622	17.0022	0.0476	0.00E+00
TTCCTCCC	-11	25	13.5417	0.1251	8.7296	12.4966	0.0382	1.94E-14
CCCTTCCT	-11	43	21.1864	0.2094	6.8038	17.3817	0.0291	0.00E+00
TCGCCTCG	-11	25	17.9153	0.1228	6.5240	14.1587	0.0252	2.05E-12
CGTCTCCC	-11	13	17.9487	0.0755	7.0543	11.4277	0.0186	5.03E-07
CCCTCTCG	-11	22	19.7740	0.1272	6.8755	10.8178	0.0178	2.48E-11
CTCCTCCC	-12	73	12.5759	0.2271	8.0018	17.8483	0.0931	0.00E+00
TCCTCTCC	-12	111	15.6904	0.2958	7.4478	20.2914	0.0871	0.00E+00
TCCTCCCT	-12	22	11.6788	0.0949	6.7766	11.7428	0.0469	2.19E-12
CTCGCCTC	-12	26	23.8663	0.1466	9.8572	20.9898	0.0374	0.00E+00
CGCTCGCT	-12	15	15.3257	0.0888	6.4760	10.1518	0.0205	1.29E-08

GCCGCTTC	-12	10	22.3881	0.0726	7.3101	13.7120	0.0162	2.51E-06
CTCTCGAG	-12	9	23.8095	0.0869	7.8201	12.4069	0.0106	2.28E-06
TCCTCTCT	-13	68	14.0244	0.2176	6.7047	16.6505	0.0653	0.00E+00
CCTCCCCT	-13	64	13.3615	0.2263	6.9648	14.3384	0.0592	0.00E+00
TTCCTCTC	-13	24	12.5786	0.0991	7.0518	12.0053	0.0483	5.77E-13
CACCCACC	-13	25	17.4263	0.1087	7.9169	13.8835	0.0397	4.32E-13
CCTCTCTT	-13	19	11.2994	0.0793	6.3645	10.8080	0.0343	1.87E-09
CCTCGCCT	-13	27	20.7317	0.1343	7.2665	18.2598	0.0312	2.68E-14
CTCCCCTT	-13	63	13.1926	0.2531	6.0876	11.2341	0.0307	0.00E+00
GTCGTCTT	-13	10	19.4444	0.0781	7.1125	10.8993	0.0137	2.32E-06
GCGAGCCC*	-13	7	23.2558	0.0745	9.4786	10.4828	0.0107	2.18E-05
CCCTCGCC	-14	21	11.4723	0.0797	6.6868	11.0774	0.0383	6.80E-10
TTCCTCTT	-14	10	10.1523	0.0487	7.4647	10.4143	0.0380	5.23E-06
CCCCTCCA	-14	32	15.0602	0.1194	6.0267	12.3415	0.0311	5.13E-13
TCCTCTCG	-14	21	14.8148	0.1115	6.3419	10.4520	0.0223	5.98E-11
CCCCCACG	-14	17	16.5877	0.0879	6.3304	10.8933	0.0196	2.22E-08
CCCCGTCT	-14	20	26.3158	0.1300	7.4194	11.3926	0.0106	1.42E-09
CCCCGCAT	-14	12	32.0513	0.0920	7.6995	12.2652	0.0080	5.54E-06
CTCTCTCT	-15	177	17.0352	0.3719	9.0536	33.4247	0.2781	0.00E+00
CTCCTCTC	-15	101	15.7116	0.3126	9.1522	21.3981	0.1011	0.00E+00
CTTCCTCT	-15	26	14.2857	0.1056	7.1855	16.0315	0.0524	5.28E-14
CTCCTCCG	-15	11	9.9291	0.0510	7.3924	10.2766	0.0486	4.88E-07
TCCACCTC	-15	11	12.8205	0.0553	9.5235	13.5361	0.0419	3.47E-07
TCCTCCGC	-15	10	11.6054	0.0515	8.6100	11.0615	0.0379	2.20E-06
CCCCTCGC	-15	23	15.9011	0.1238	8.1406	12.2946	0.0277	6.76E-13
CCCTCGCT	-15	18	18.5185	0.1321	7.2737	10.5840	0.0136	1.25E-10
TCTCTCTC	-16	180	15.3689	0.3627	9.4789	32.3708	0.2936	0.00E+00
CTCCTCCT	-16	80	11.9048	0.2543	10.8696	21.3438	0.1576	0.00E+00
CCTCTCCT	-16	91	13.7457	0.2884	7.4292	16.1734	0.0782	0.00E+00
CCCACCAC	-16	25	12.4521	0.0870	7.8282	12.0526	0.0522	5.99E-12
CTCCACCT	-16	12	10.6383	0.0510	6.7514	11.1645	0.0444	9.02E-07
TCCTTCCT	-16	15	12.1269	0.0777	8.9992	11.6244	0.0407	6.75E-10
CTCGTCTC	-16	21	19.1571	0.1610	11.0170	12.7267	0.0273	9.99E-16
CACCTCAC	-16	16	14.8148	0.0698	6.1312	10.0271	0.0262	1.71E-07
CGCCCCGC	-16	12	15.6250	0.0610	6.2794	11.8988	0.0231	3.79E-06
TCTCCTCC	-17	80	13.0964	0.2690	8.7006	17.2357	0.0897	0.00E+00
TTCCCCTC	-17	25	10.8911	0.1022	7.7449	10.0106	0.0407	1.53E-12
TCGTCTCC	-17	19	17.3611	0.1341	10.1505	12.2752	0.0280	8.52E-14
CGAAGCGA*	-17	11	29.1667	0.0766	6.4740	13.9487	0.0105	1.65E-05
CCTCCTCC	-18	111	10.7204	0.2580	8.1719	19.6229	0.1918	0.00E+00
CCTCCACC	-18	22	11.2137	0.0979	8.7638	12.6226	0.0580	1.26E-13
CCCCCACC	-18	90	16.0256	0.2546	7.5797	15.8687	0.0557	0.00E+00
CCTTCTCC	-18	28	10.2180	0.1033	6.1626	10.6179	0.0525	9.15E-14
CCCTCTCT	-18	24	11.8715	0.1048	7.9551	13.2134	0.0522	6.66E-14
ATCTCCTC	-18	24	10.3578	0.0982	7.3157	10.0796	0.0384	6.83E-12
TCTCGCCT	-18	24	29.7619	0.1422	6.4216	16.4820	0.0157	6.50E-12

GTCTCCCC	-18	12	19.6078	0.0755	6.5792	10.9805	0.0137	3.87E-06
ACCCCTCG	-18	19	34.0909	0.1427	8.4305	14.6826	0.0098	4.23E-10
CTCCCTCT	-19	21	9.1463	0.0785	7.3996	11.3392	0.0652	7.74E-12
TCCCTCTC	-19	25	11.9048	0.1018	7.8646	14.0435	0.0594	3.70E-14
CCCCCCTC	-19	23	15.5678	0.1331	11.4396	14.8613	0.0495	0.00E+00
ACCCCCAC	-19	23	22.9167	0.1213	7.9495	15.9601	0.0261	1.98E-12
CACGCCCC	-19	13	21.3675	0.0750	6.0077	10.4587	0.0113	1.41E-05
CCTCTCTC	-20	108	14.2450	0.2745	6.5012	18.4598	0.0890	0.00E+00
CCTCCCTC	-20	20	11.0865	0.0969	10.1299	12.4448	0.0726	1.57E-14
CCCTTCTC	-20	44	13.2325	0.1659	6.3102	12.0114	0.0411	0.00E+00
GCTCTCTC	-20	19	14.1176	0.0966	6.9684	11.8238	0.0306	7.92E-11
CTCTCGCC	-20	23	20.0000	0.1193	6.7089	14.9027	0.0251	4.25E-12
CGCCCCCC	-20	20	22.6244	0.1173	7.4479	14.3519	0.0218	2.37E-11
CCCCCCCC	-21	111	22.8070	0.3142	8.6801	25.3907	0.1727	0.00E+00
CCCCTCTC	-21	25	13.8889	0.1325	10.6505	15.6637	0.0559	0.00E+00
CCCACCCA	-21	51	14.8810	0.1732	6.2809	11.1346	0.0389	0.00E+00
CCCCCCAC	-21	66	18.5185	0.2618	6.3632	12.4479	0.0307	0.00E+00
CCACCCCA	-21	27	17.9211	0.1075	6.3134	13.4663	0.0292	1.14E-11
TCCCCCCC	-21	26	21.6667	0.1491	7.8346	15.8868	0.0272	7.55E-15
CACCCCCA	-21	22	22.9167	0.0913	6.2919	15.0814	0.0271	7.61E-10
CCTCGCTC	-21	21	16.0643	0.1232	6.5108	11.3763	0.0194	2.29E-11
CGCCCCCA	-21	24	27.7778	0.1214	6.0504	15.9572	0.0165	1.39E-10
CGCTCTCT	-21	15	22.3881	0.1229	8.1627	12.4445	0.0164	1.08E-10
TCCCCACG	-21	21	20.0000	0.1196	6.0534	10.8784	0.0139	8.33E-10
CACTCGGT	-21	16	28.5714	0.1548	6.2527	10.4411	0.0047	3.38E-08
CCCTCCTC	-22	91	13.4228	0.2467	7.0528	17.9098	0.0785	0.00E+00
CCCCACCC	-22	100	21.7984	0.3020	7.9789	18.1345	0.0544	0.00E+00
CCCCTCCT	-22	23	15.6495	0.1403	11.3047	16.3491	0.0509	0.00E+00
CCCACCCC	-22	24	20.9497	0.1101	10.4584	16.5065	0.0482	4.50E-14
CCCCTTCC	-22	72	18.6722	0.2526	6.1357	17.2031	0.0383	0.00E+00
TCGCTCTC	-22	17	16.4835	0.1137	7.4442	10.6583	0.0218	4.62E-11
CCACTCGG	-22	10	26.8817	0.0829	6.0874	11.2297	0.0068	6.83E-05
CCACCTCC	-23	73	11.6279	0.2126	6.0788	12.2389	0.0637	0.00E+00
CTCCCCCC	-23	81	16.8421	0.2624	6.6791	15.1915	0.0451	0.00E+00
TCCCCTTC	-23	73	16.7715	0.2950	8.3266	16.8485	0.0411	0.00E+00
ACCCCTCC	-23	27	18.8679	0.1333	7.4200	14.9131	0.0290	6.89E-14
CGTCTCCT	-23	18	18.0328	0.1467	10.2528	13.4687	0.0254	2.43E-14
TCTCGCTC	-23	19	19.4444	0.1081	6.1661	10.6164	0.0166	1.50E-09
ACCCCACC	-24	76	21.4844	0.2668	6.1192	14.9093	0.0315	0.00E+00
TCTCTCGC	-24	25	22.0884	0.1350	7.2119	14.9271	0.0242	3.13E-13
CTCTCGCT	-24	23	19.6078	0.1383	7.1697	12.4765	0.0190	1.94E-12
CCCCCTCC	-25	81	16.4159	0.2699	7.2781	18.3559	0.0653	0.00E+00
CCCCACCT	-25	23	20.8333	0.0944	7.6154	15.8740	0.0375	2.09E-11
CCCATCCC	-25	28	19.7842	0.1229	7.1918	14.1089	0.0311	2.51E-13
CCATCCCC	-25	15	15.9236	0.0609	6.1313	11.9914	0.0301	6.72E-07
ACCACCCC	-25	20	21.3178	0.0914	6.2257	15.0898	0.0246	1.69E-09

AAGCCCCA	-25	14	21.3675	0.0887	6.2400	10.1685	0.0108	1.41E-06
ACCCCCCG	-25	12	35.7143	0.0793	7.2786	15.1559	0.0092	2.12E-05
TCCCCCTC	-26	22	14.4558	0.1173	10.6344	16.0588	0.0465	1.27E-14
ATCCCCGC	-26	14	23.3645	0.0962	6.7108	10.4784	0.0101	5.98E-07
TACCCCCC	-26	17	32.0513	0.1196	7.8801	12.4259	0.0089	3.04E-08
AACCCCCC	-27	18	32.5444	0.1478	10.9333	18.4925	0.0190	3.88E-13
AAGCCCTC	-27	14	25.7353	0.1127	6.3954	12.3121	0.0099	4.00E-08
AACCCCCG	-27	14	37.8788	0.1117	8.5974	14.1227	0.0074	4.07E-07
AAAGCCGG	-27	11	27.7778	0.1005	6.5317	11.3621	0.0064	5.73E-06
TAAGCGCG	-27	15	50.0000	0.1558	11.6656	12.3672	0.0038	1.49E-07
AAACCCCC	-28	48	38.9908	0.2462	7.1302	26.2326	0.0229	0.00E+00
AAACCCAC	-28	18	35.2941	0.1293	8.5417	24.1859	0.0217	2.58E-12
TAAACCCA	-28	15	21.6667	0.1138	8.1833	16.3791	0.0209	6.90E-11
AACCCCTC	-28	26	25.6410	0.1936	8.4978	16.9680	0.0200	0.00E+00
ATACCCCC	-28	8	42.5532	0.1240	13.1343	21.7202	0.0108	3.26E-09
AAACCCGC	-28	12	30.6122	0.0847	7.8216	13.4593	0.0106	3.35E-06
TAAGTAGC	-28	11	23.8095	0.1273	8.1972	13.2261	0.0102	3.09E-09
ATAACCCC	-28	14	30.3030	0.1252	7.2389	15.8313	0.0097	6.66E-09
AAAAGCCG	-28	10	44.8718	0.1137	12.6338	16.9005	0.0089	7.91E-08
AACCCTCG	-28	19	30.8642	0.1664	8.4704	12.4068	0.0079	1.03E-10
TATATCGC	-28	10	33.3333	0.1583	11.1591	13.7194	0.0063	1.98E-09
TATACCGC	-28	7	41.6667	0.1016	11.5550	12.9441	0.0041	6.71E-05
AAAAACCC	-29	11	22.5141	0.0659	12.1580	23.7622	0.0424	1.30E-08
ATATACAC	-29	12	13.9147	0.0807	8.7563	13.3098	0.0320	2.35E-09
AAACCCCA	-29	36	27.3723	0.2038	7.7210	19.0282	0.0265	0.00E+00
AAATCCCC	-29	27	29.9625	0.1535	6.8767	19.6088	0.0236	1.63E-14
AATCCCCC	-29	31	34.5912	0.1636	6.7891	19.4547	0.0176	6.99E-14
TAAAAGGG	-29	12	23.5294	0.1086	7.9823	14.8946	0.0170	1.12E-09
ATCCCCCC	-29	12	19.4444	0.0809	6.5863	10.8409	0.0147	7.88E-07
TAAAAGGC	-29	12	24.3902	0.1287	9.8688	15.7522	0.0146	1.16E-10
ACCCCCCT	-29	25	33.0579	0.1912	9.1043	15.7081	0.0135	2.12E-14
TATACCCC	-29	15	34.4828	0.1416	7.6981	19.9955	0.0120	8.53E-11
ATACCCCT	-29	9	18.8172	0.0743	6.2841	10.5006	0.0117	1.33E-05
ATAAGCTC	-29	12	30.2013	0.0970	7.0157	15.6339	0.0116	1.87E-07
AAAAGCGG	-29	14	27.7778	0.1177	6.7418	15.0788	0.0102	1.43E-08
TATAACCC	-29	9	22.4359	0.0781	6.7157	13.2399	0.0102	1.45E-05
TAAAGCCC	-29	12	21.9298	0.0783	6.1622	10.1634	0.0102	1.40E-05
AAAGCCGC	-29	11	29.4118	0.0832	7.8179	11.7339	0.0089	1.36E-05
AATAGCCG	-29	8	30.7018	0.1032	9.5766	14.5559	0.0080	8.88E-07
ATAAACGC	-29	9	26.8817	0.0805	6.8295	11.2297	0.0070	8.12E-05
AATCCCCG	-29	8	30.8642	0.0849	8.7412	11.8003	0.0068	4.75E-05
TAAACCCG	-29	7	50.0000	0.0933	13.2703	16.8452	0.0063	1.89E-05
ATATCGCC	-29	9	30.7692	0.1108	9.5215	11.1097	0.0052	4.61E-06
ATAGCCGG	-29	8	37.5000	0.1420	10.3017	15.2478	0.0047	1.61E-07
TATAACGC	-29	12	32.7869	0.1725	9.7322	11.6221	0.0043	7.84E-09
TATACGCC	-29	15	33.3333	0.1841	9.0526	10.1582	0.0036	9.69E-09

TAAAAACC	-30	17	20.3046	0.0823	6.8388	18.8340	0.0388	3.06E-10
TAAATACT	-30	10	11.4943	0.0586	7.6935	12.0310	0.0339	5.60E-07
TATAAGTA	-30	11	12.8913	0.0764	9.1294	12.0272	0.0326	5.92E-09
TATATAGC	-30	15	14.7059	0.0923	9.2013	14.4387	0.0317	1.57E-10
AAAACCCC	-30	35	32.6705	0.2155	8.6047	26.3160	0.0298	0.00E+00
ATAAAAGC	-30	22	31.9489	0.1809	8.9609	23.7141	0.0221	0.00E+00
TATAAGCA	-30	14	20.1613	0.0852	6.2565	17.2902	0.0212	2.22E-08
TAAACCCT	-30	20	25.0965	0.1194	6.4207	16.8804	0.0203	3.05E-11
ATATACCC	-30	10	25.2918	0.0869	8.2019	17.2677	0.0175	6.63E-08
TTAAACCC	-30	13	38.4615	0.1401	10.8917	21.6609	0.0153	1.11E-11
TAAACCCC	-30	21	84.0000	0.3236	19.5141	41.6673	0.0147	0.00E+00
GATAAACC	-30	10	21.5054	0.0952	8.2549	11.8489	0.0136	8.06E-08
TAATCCCC	-30	11	41.0448	0.1238	12.3405	20.2549	0.0135	5.21E-10
ATAAAGCC	-30	16	29.2398	0.1308	7.0097	17.4694	0.0122	3.89E-10
AATACCCC	-30	13	43.3333	0.1375	9.6224	24.0628	0.0120	1.62E-10
TAAATCCG	-30	13	27.2727	0.1018	6.4178	14.7439	0.0117	7.41E-08
TATAAGCG	-30	11	20.9790	0.0969	6.1469	10.3496	0.0090	1.11E-06
AATACCCG	-30	11	22.5225	0.1171	7.7971	10.8731	0.0077	1.40E-07
TAAAAGCG	-30	7	43.0108	0.1008	11.7896	19.0790	0.0075	2.07E-06
ATACACGC	-30	14	31.5789	0.1204	6.7637	13.3192	0.0074	1.10E-07
TATACGCG	-30	9	26.8817	0.0979	7.9279	11.2297	0.0067	6.67E-06
ATAGCCCC	-30	7	29.0698	0.0961	9.6107	11.6836	0.0060	1.65E-05
TAAGCCCC	-30	13	31.7460	0.1301	8.6391	11.0745	0.0057	1.81E-07
ATAACCGC	-30	6	44.8718	0.1090	12.2100	16.6977	0.0054	4.94E-06
ATAAATAA	-31	16	7.4875	0.0383	6.2048	13.6329	0.1074	2.47E-07
ATATATAG	-31	17	11.5845	0.0641	7.2502	16.8527	0.0766	1.62E-10
AAACCCTA	-31	57	14.3824	0.2027	6.3263	14.3961	0.0459	0.00E+00
ACAAAACC	-31	8	12.6263	0.0407	9.3563	13.3978	0.0450	2.69E-05
TATAAATC	-31	16	15.2990	0.0964	8.2173	16.0885	0.0429	3.64E-12
TATAAATG	-31	15	16.2003	0.1050	10.6242	18.7790	0.0401	9.19E-13
ATATAAAG	-31	10	15.8371	0.0714	10.0022	17.4012	0.0385	5.94E-09
ATAAAACC	-31	16	17.7570	0.0968	9.7411	16.9473	0.0375	1.17E-11
ATAAAAGG	-31	11	27.5229	0.1428	21.0228	28.9696	0.0368	8.88E-16
CAAAACCC	-31	33	28.0000	0.1666	8.0593	24.6869	0.0326	0.00E+00
ATAAATCC	-31	25	23.2274	0.1309	7.0692	20.7688	0.0290	1.08E-13
ATATAAGC	-31	20	37.9581	0.1916	11.1039	33.3288	0.0247	0.00E+00
AAAAGCCC	-31	13	21.8254	0.0783	7.9107	14.0371	0.0245	3.53E-08
TAAAACCC	-31	39	32.9861	0.1949	6.0449	23.6003	0.0235	0.00E+00
AAATACCC	-31	14	44.0141	0.1915	15.7609	29.5686	0.0227	0.00E+00
ATAAACCC	-31	24	67.2646	0.2936	15.1696	42.4786	0.0214	0.00E+00
TAAATAGG	-31	15	24.3506	0.1166	7.8556	18.8594	0.0196	7.26E-11
AAATAGCC	-31	17	25.0896	0.1079	6.7302	18.9410	0.0194	4.06E-10
TAAATAGC	-31	17	48.8281	0.2049	12.5753	35.5382	0.0185	0.00E+00
ATAAGTAG	-31	11	18.3824	0.0918	8.0108	12.2769	0.0182	1.75E-08
TATAAACG	-31	11	30.5556	0.1197	7.9138	17.9566	0.0108	6.67E-09
AAATACGC	-31	10	36.2319	0.1072	9.2583	18.6323	0.0107	5.58E-08

ATAAAGGC	-31	14	43.1655	0.1684	9.8897	22.4239	0.0103	7.01E-12
ATATACGC	-31	17	40.6504	0.1890	9.4015	20.7051	0.0096	9.14E-13
ATAAGCCC	-31	13	29.4118	0.1236	8.2470	12.7455	0.0086	2.10E-08
ATAAAGCG	-31	12	26.0417	0.0992	6.4026	11.2239	0.0073	3.12E-06
TATATATG	-32	13	8.0813	0.0446	7.8347	13.5733	0.1092	1.35E-08
ATATATAC	-32	18	18.6294	0.1037	14.7589	33.3103	0.0927	0.00E+00
TTATAAAT	-32	12	8.0815	0.0397	6.9251	11.5508	0.0825	8.33E-07
ATAAAAAG	-32	10	10.4167	0.0411	7.4305	13.1595	0.0641	2.61E-06
ATAAAAAC	-32	17	16.0085	0.0726	8.1241	19.8974	0.0627	6.56E-11
TATATATC	-32	18	12.4575	0.0844	8.5772	15.2227	0.0584	4.15E-12
ATATAAAC	-32	18	27.9543	0.1347	13.0088	34.3717	0.0532	0.00E+00
TATATAAG	-32	18	29.4479	0.1582	14.2345	35.9855	0.0507	0.00E+00
ATAAATTC	-32	13	11.4943	0.0581	6.6407	12.7515	0.0457	7.61E-08
GTATATAA	-32	16	18.1311	0.0988	10.2624	23.1313	0.0442	1.51E-12
GTATAAAT	-32	13	20.9790	0.1134	14.3417	23.0266	0.0365	3.67E-13
TAAAAGGA	-32	9	13.2576	0.0577	9.0713	12.9774	0.0322	1.02E-06
ACAAATAC	-32	15	12.0370	0.0705	6.0224	10.9734	0.0307	4.44E-08
TATTTAAG	-32	10	15.1515	0.0739	8.8336	14.5771	0.0283	3.41E-08
TATAAGAG	-32	11	15.6250	0.0764	7.4597	13.0491	0.0241	6.27E-08
TAAATACC	-32	19	61.4035	0.3005	17.6729	40.5680	0.0193	0.00E+00
ATATAGCC	-32	12	16.3636	0.0950	6.9210	11.5357	0.0165	1.99E-08
TATAAAGG	-32	17	32.8947	0.1603	8.2168	21.5147	0.0150	5.62E-13
ATAAACCG	-32	15	22.3881	0.1128	6.3460	13.9266	0.0133	4.08E-09
TAAATACG	-32	13	34.4828	0.1609	11.0534	20.4849	0.0128	2.43E-12
TAAAACGC	-32	11	42.8571	0.1170	8.8189	19.1667	0.0084	6.85E-08
TATATATA	-33	15	6.4748	0.0353	10.4325	19.2989	0.6213	3.99E-13
TATAAAAA	-33	9	6.4217	0.0268	8.2932	12.5188	0.1601	5.70E-06
TATAAATT	-33	10	12.1791	0.0496	11.7709	20.4946	0.0901	7.80E-09
CTATAAAA	-33	22	43.9625	0.1956	18.3418	57.7497	0.0633	0.00E+00
TATAAAAG	-33	20	27.2727	0.1412	13.2358	38.0948	0.0556	0.00E+00
TACAAATA	-33	14	12.2732	0.0589	6.3342	14.2220	0.0516	2.34E-08
ATAAATAC	-33	20	42.4460	0.2132	18.6311	48.8170	0.0502	0.00E+00
CATAAATA	-33	10	11.9904	0.0546	8.3538	14.8848	0.0464	2.09E-07
ATAAATAG	-33	19	35.7644	0.1702	13.7517	40.5377	0.0455	0.00E+00
CTTATAAA	-33	11	12.1951	0.0640	8.0010	12.9158	0.0374	5.96E-08
TATATAAC	-33	15	21.1382	0.1129	10.1722	22.6544	0.0362	4.35E-13
CTATTTAA	-33	12	22.2635	0.1036	11.9135	21.7553	0.0320	1.10E-11
TATAAACC	-33	19	42.3077	0.2286	14.8804	34.0883	0.0281	0.00E+00
ATAAAACG	-33	9	13.8889	0.0581	8.4636	11.8490	0.0279	2.43E-06
GCATAAAT	-33	11	14.3885	0.0621	6.6348	12.7416	0.0240	2.26E-06
CCTATTTA	-33	17	23.6686	0.1379	8.8044	18.4693	0.0224	4.04E-13
CTATAAGA	-33	9	24.6212	0.1054	10.6182	16.9362	0.0170	2.00E-09
GGTATAAA	-33	18	24.2537	0.1432	6.6817	15.6046	0.0164	3.43E-12
TAAAAGCC	-33	13	37.0370	0.1587	12.5764	23.0977	0.0157	4.62E-13
AAATACCG	-33	12	24.2424	0.0840	6.1538	13.9639	0.0122	1.49E-06
TATATAAA	-34	13	15.8133	0.0823	16.3336	28.7279	0.1240	3.55E-15

ATATATAA	-34	12	8.5075	0.0464	10.3989	16.3417	0.1186	3.21E-09
TATAAATA	-34	18	37.4214	0.1769	23.7440	59.5464	0.1110	0.00E+00
CATATATA	-34	12	7.3242	0.0423	8.2543	12.9475	0.1107	3.25E-08
CTATATAT	-34	22	32.4859	0.1718	17.6105	51.0250	0.0936	0.00E+00
GTATATAT	-34	17	16.3666	0.0841	10.7307	25.0968	0.0793	2.26E-13
TGTATATA	-34	14	15.0632	0.0775	11.1244	21.6068	0.0629	1.48E-11
CTATATAA	-34	18	51.6432	0.2369	24.6610	65.7269	0.0604	0.00E+00
AGTATATA	-34	16	11.9048	0.0710	8.0116	15.1367	0.0575	2.24E-10
TATAAAAC	-34	12	19.1327	0.0922	11.7589	21.4260	0.0506	2.09E-12
CCAAAACC	-34	15	14.7059	0.0704	9.5235	14.1000	0.0490	1.08E-09
GTATAAAA	-34	17	10.8043	0.0619	6.0536	13.9389	0.0477	1.71E-08
GCTATAAA	-34	17	52.8942	0.2440	24.8896	51.4848	0.0378	0.00E+00
CTACAAAT	-34	16	15.4976	0.0792	6.5049	15.2095	0.0360	1.27E-09
CTACAAAA	-34	10	13.2509	0.0466	6.1670	13.0759	0.0337	2.08E-05
TCTATTTA	-34	10	12.9534	0.0628	8.0932	12.6179	0.0329	2.15E-07
CTATAAAC	-34	8	11.8830	0.0506	7.7042	11.6343	0.0291	1.53E-05
CTATATAG	-34	13	17.3611	0.0821	6.7333	15.3944	0.0228	2.56E-08
CGTATAAA	-34	14	22.8659	0.1273	9.2828	18.9194	0.0200	8.22E-12
GCTATTTA	-34	9	19.0476	0.0685	7.0493	14.7490	0.0181	2.97E-06
GCCTATTT	-34	9	18.5185	0.0668	6.6948	13.9270	0.0174	5.70E-06
GGGTATAA	-34	11	28.1250	0.1049	7.3933	15.4845	0.0108	8.20E-08
CCTATAAG	-34	10	22.7273	0.1096	7.3141	10.7849	0.0081	2.89E-07
CGGGTATA	-34	9	36.2319	0.1045	8.9988	13.4220	0.0055	7.96E-06
GCCGTTAT	-34	11	35.7143	0.1303	8.1657	10.5018	0.0032	9.17E-06
TCTTCTTC	-35	80	10.5541	0.1980	6.3556	16.7220	0.1069	0.00E+00
CTATAAAT	-35	21	76.0135	0.3164	31.4747	93.3235	0.0724	0.00E+00
TCTATATA	-35	17	30.6022	0.1466	18.4127	46.2615	0.0619	0.00E+00
ACTATATA	-35	18	15.1844	0.0777	6.8953	18.4413	0.0535	6.08E-11
TCTATAAA	-35	19	30.3398	0.1649	15.2919	39.0755	0.0524	0.00E+00
CCTATATA	-35	19	59.7381	0.2778	26.4687	66.5570	0.0467	0.00E+00
ACTATAAA	-35	11	22.4490	0.1092	14.8360	26.4974	0.0437	1.48E-13
TGTATAAA	-35	11	11.4094	0.0480	6.4543	13.1617	0.0431	2.91E-06
TCCTATAT	-35	10	15.1745	0.0871	12.3807	17.6915	0.0370	1.40E-10
GCTATATA	-35	14	40.0372	0.2131	25.6171	45.9040	0.0354	0.00E+00
CTCTCTAT	-35	18	15.2225	0.0933	7.1930	14.2536	0.0282	3.50E-10
AGCTATAT	-35	12	12.3457	0.0653	7.0162	11.8304	0.0271	4.30E-07
AGCTATAA	-35	16	17.4419	0.0865	6.4370	15.5956	0.0248	4.74E-09
CTCTATAA	-35	22	35.0404	0.1792	9.7806	29.5847	0.0238	0.00E+00
CCTACAAA	-35	11	20.1149	0.0796	8.0289	16.5158	0.0226	4.83E-08
GTCTATAT	-35	13	21.8023	0.0809	6.6080	18.6037	0.0207	6.94E-08
GGCTATAT	-35	15	23.3333	0.1185	7.6911	18.2168	0.0186	8.16E-11
CCTTTATA	-35	14	16.6113	0.0923	6.3046	12.6413	0.0183	1.54E-08
CTGTATAA	-35	9	18.9076	0.0751	6.9497	12.4287	0.0147	2.65E-06
CGCATAAA	-35	12	25.4237	0.0932	6.2515	14.8857	0.0119	3.21E-07
CGCTATAA	-35	12	166.6667	0.4339	46.2304	67.8805	0.0094	0.00E+00
CGCCTACA	-35	11	26.0870	0.1016	7.3004	11.3682	0.0088	6.07E-07

GCGTATAA	-35	12	34.3137	0.1782	9.4666	13.4609	0.0065	1.33E-10
CGCCTATT	-35	7	43.2099	0.1347	13.2069	17.3090	0.0056	1.08E-07
CGCGCTAT	-35	14	59.5238	0.2638	15.8729	17.7047	0.0039	2.33E-12
CCTATAAA	-36	16	89.0411	0.3268	40.7575	88.7627	0.0464	0.00E+00
CTCTATAT	-36	19	35.1967	0.1851	13.4197	31.2831	0.0313	0.00E+00
CCATATAA	-36	9	15.4827	0.0667	9.5687	15.1717	0.0308	1.18E-07
GTGTATAT	-36	16	16.8067	0.0936	8.1704	16.3858	0.0291	2.48E-10
TCCTATAA	-36	11	24.2131	0.1021	11.1956	21.1565	0.0258	1.09E-10
CACTATAT	-36	16	23.2843	0.1027	7.3125	19.8425	0.0245	1.51E-10
CTCCTATA	-36	16	36.9231	0.1918	13.5850	30.7489	0.0223	0.00E+00
ACCTATAA	-36	15	24.7525	0.1182	8.6296	18.4602	0.0215	2.19E-11
GCCTATAT	-36	21	45.7516	0.2344	12.3113	36.5147	0.0213	0.00E+00
CAGCTATA	-36	17	18.9655	0.1046	6.5121	14.3858	0.0199	6.20E-10
CACCTATA	-36	11	35.0877	0.1556	13.9762	24.5507	0.0192	1.02E-13
TGTCTATA	-36	13	20.4082	0.0971	6.3495	15.0444	0.0167	1.17E-08
TCTATAAG	-36	9	16.6052	0.0684	6.3117	11.4117	0.0159	6.97E-06
CTGCTATA	-36	12	28.2051	0.0969	6.6060	15.8731	0.0131	7.87E-08
CCTCTTTA	-36	11	20.6186	0.0955	6.4403	12.5790	0.0116	2.51E-07
CCGTATAA	-36	14	31.2500	0.1072	6.1851	17.6086	0.0111	4.38E-08
CCCCAATA	-36	10	22.8571	0.0910	6.4579	12.7461	0.0105	1.14E-06
GGTCTATA	-36	8	18.5185	0.0854	7.3958	10.8919	0.0094	6.32E-06
CGCTATAT	-36	13	53.0303	0.2534	14.9695	26.3936	0.0093	0.00E+00
GCCTTTAT	-36	10	21.2766	0.0855	6.2931	10.9033	0.0093	6.60E-06
GCTGTATA	-36	9	25.0000	0.0933	6.3002	11.6875	0.0069	1.17E-05
TCGCCTAT	-36	10	64.5161	0.2741	20.3995	27.5586	0.0069	2.44E-15
TCGCTATA	-36	10	41.6667	0.1865	13.1466	16.8170	0.0068	3.41E-11
CGACTATA	-36	18	32.0513	0.1756	6.8121	12.4259	0.0055	7.31E-10
CCCCGCTA	-36	11	49.0196	0.1362	11.0261	14.6474	0.0049	2.36E-07
GCGCTATA	-36	9	47.6190	0.1834	12.8140	17.6831	0.0041	3.48E-09
TCTCTATA	-37	14	18.3824	0.1036	9.0454	17.1879	0.0311	1.46E-11
CGTATATA	-37	11	15.3846	0.0646	6.3556	10.6504	0.0247	9.71E-07
GGCTATAA	-37	16	24.4648	0.1481	9.2011	18.3989	0.0205	1.74E-13
CCCTATAT	-37	17	42.9553	0.2092	12.5473	31.5110	0.0201	0.00E+00
CACTATAA	-37	11	43.4028	0.1722	15.3454	31.5993	0.0193	6.88E-15
CCCTATAA	-37	16	85.5615	0.3048	20.3396	48.8604	0.0167	0.00E+00
GCCTATAA	-37	15	102.3392	0.3732	28.2542	63.5279	0.0161	0.00E+00
CCTTATAA	-37	13	19.1571	0.1035	6.6460	12.5467	0.0159	4.99E-09
CCTCTATA	-37	18	52.3810	0.2707	13.8459	35.5573	0.0148	0.00E+00
GTCTATAA	-37	13	30.9917	0.1621	10.6747	22.8790	0.0146	5.81E-13
CCACTATA	-37	15	44.1860	0.1741	9.8631	26.8579	0.0146	1.01E-13
CCCCTATA	-37	17	81.9672	0.2806	14.8139	50.1809	0.0135	0.00E+00
GTCCTATA	-37	10	20.1005	0.0762	6.0034	12.6644	0.0123	6.60E-06
CCCCCGCT	-37	13	25.2525	0.0864	7.8718	11.4742	0.0113	1.57E-06
GCACTATA	-37	9	24.5902	0.0852	7.0450	14.2955	0.0113	2.07E-06
GCTCTATA	-37	13	43.3333	0.1621	10.3966	21.6948	0.0112	7.34E-12
CCCCCATA	-37	17	24.3902	0.1197	6.6296	11.9994	0.0107	6.92E-09

GCCTCTAT	-37	10	29.1667	0.1097	8.0260	14.1930	0.0082	2.72E-07
CGGCTATA	-37	14	37.6344	0.2374	14.1632	14.5327	0.0078	2.53E-14
ACGCTATA	-37	7	29.6610	0.0908	8.2409	12.9492	0.0072	1.29E-05
CGTCTATA	-37	13	51.7241	0.1930	11.8757	21.7908	0.0069	1.24E-11
GGCTCTAT	-37	7	32.6087	0.0842	8.9671	13.1864	0.0069	5.07E-05
TTCGCCTA	-37	9	27.3973	0.0975	8.5535	10.4140	0.0059	1.51E-05
ACGCTACA	-37	9	27.7778	0.1166	8.1137	11.3621	0.0056	1.30E-06
CGCGCCTA	-37	15	28.0899	0.1605	6.6877	11.6725	0.0055	4.53E-09
CCCGCTAT	-37	11	55.5556	0.2383	17.5974	19.9805	0.0054	1.08E-12
CCCGCCTA	-37	19	53.0303	0.2650	10.7635	17.6814	0.0054	8.00E-14
TCCGCTAT	-37	9	78.4314	0.2533	26.0239	24.0538	0.0051	4.06E-13
CCGCGCTA	-37	11	28.9855	0.1139	7.2934	11.0028	0.0050	3.99E-06
CCGTCTAT	-37	10	38.4615	0.1539	11.0807	13.7552	0.0049	2.70E-08
CGCCTTTA	-37	13	41.6667	0.1696	8.9765	13.5420	0.0044	9.84E-09
GCGTCTAT	-37	10	27.7778	0.1104	6.5657	10.6324	0.0044	1.45E-05
GCGCCCTA	-37	12	37.7358	0.1684	9.8134	12.6369	0.0040	2.58E-08
CCCGTCTA	-37	11	55.5556	0.1727	13.3276	13.0213	0.0034	4.78E-08
ACTCTATA	-38	9	13.7363	0.0658	6.9684	10.6956	0.0198	3.16E-06
TCTCCTAT	-38	10	16.0772	0.0651	6.2940	12.1854	0.0188	5.16E-06
TGCCTATA	-38	20	46.0829	0.2196	9.7780	30.2457	0.0149	0.00E+00
TCCCTATA	-38	15	40.4040	0.2049	12.7052	24.7971	0.0146	1.44E-15
ACCCTATA	-38	12	25.2525	0.1204	8.5971	15.9204	0.0135	9.62E-10
GGCCTATA	-38	14	28.7356	0.1260	7.1755	17.4055	0.0114	1.49E-09
TCCCCCTA	-38	7	26.7857	0.0792	8.9109	14.6911	0.0113	6.17E-06
CGCCTATA	-38	16	164.5570	0.5231	41.5309	69.7387	0.0101	0.00E+00
CCCCCTAT	-38	15	57.2519	0.1914	10.6131	29.4845	0.0100	4.62E-13
CCGCCTAT	-38	18	90.3614	0.3470	19.9560	38.2064	0.0086	0.00E+00
CTCGCCTA	-38	16	27.2727	0.1336	6.6102	11.6043	0.0084	5.54E-09
CGCGTATA	-38	12	28.4553	0.1491	8.8979	13.2969	0.0083	7.20E-10
CGGCCATA	-38	10	26.5957	0.0974	7.9426	11.3101	0.0076	3.32E-06
ATCGCCTA	-38	9	23.8095	0.0910	7.0913	10.5456	0.0071	1.36E-05
GCCCCTAT	-38	9	36.2319	0.0954	9.0404	13.8518	0.0061	1.76E-05
CTCGCTAT	-38	10	27.7778	0.0941	6.2201	10.7778	0.0060	2.36E-05
CGCTCTAT	-38	9	42.2535	0.1504	11.3589	15.3244	0.0050	3.45E-08
GCGCCTAT	-38	8	41.6667	0.1452	11.5946	14.4643	0.0040	3.15E-07
CCCTCCCT	-39	34	13.5135	0.1196	6.5999	13.0773	0.0464	7.88E-15
CTCCTCTA	-39	19	22.5225	0.1143	6.4940	18.1502	0.0214	5.00E-11
CCTCCTAT	-39	21	29.1667	0.1497	6.4028	20.3249	0.0157	1.97E-12
TCCCCTAT	-39	12	22.3881	0.0939	6.4626	12.9432	0.0135	1.13E-07
CTGCCTAT	-39	15	27.7778	0.1369	6.4031	15.0645	0.0099	9.25E-10
GCCCTATA	-39	10	40.6504	0.1634	12.3891	19.8291	0.0087	6.44E-11
CCGCTATA	-39	15	218.7500	0.4622	47.7961	66.4433	0.0081	0.00E+00
CGGCCTAT	-39	12	33.3333	0.1010	6.7704	13.8748	0.0072	2.54E-06
CCGCCATA	-39	13	36.4583	0.1414	8.0571	16.2516	0.0069	8.79E-09
ACCGCTAT	-39	7	28.7356	0.1118	9.4261	11.0107	0.0052	4.32E-06
CGGTCTAT	-39	6	39.6825	0.1010	10.5365	14.1671	0.0041	7.26E-05

CTCCCCCT	-40	12	14.0977	0.0527	8.0994	14.0641	0.0429	6.53E-07
CCTCCCTA	-40	13	23.1092	0.1089	6.9091	15.0899	0.0145	3.93E-09
GCCGCTAT	-40	18	33.3333	0.1908	8.3102	12.4480	0.0060	5.85E-11
CGCCCTAT	-40	10	36.5854	0.1262	8.9746	14.7091	0.0059	2.48E-07
TCTCTCTA	-41	11	11.5163	0.0600	8.8934	11.2657	0.0354	2.74E-07
TCCCCCCA	-41	12	17.6471	0.0592	6.0212	11.9719	0.0222	7.72E-06
TCCGACCC	-46	10	24.3902	0.0779	6.2635	11.3282	0.0092	2.74E-05
CGTTTCCC	-47	11	23.2558	0.0758	6.8482	11.9238	0.0117	1.01E-05
AGCGACTC	-50	12	28.9855	0.1201	7.6855	11.1862	0.0055	9.78E-07
