# Supplementary material for: Identification of plant promoter constituents by analysis of local distribution of short sequences
Source: BMC Genomics. 2007 Mar 8;8:67. doi: 10.1186/1471-2164-8-67 (PMC1832190; doi:10.1186/1471-2164-8-67)
Supplement: Additional file 5 — Arabidopsis REG octamers (Table S4.pdf). Contains octamer sequences and parameters. [file 1471-2164-8-67-S5.pdf]

Table S4. Arabidopsis REG octamers

Sequence	Peak Position	Peak Width	RPH	RPA	Peak Area/ basal fluctuation	(Peak hight-Base Line)/sd	Occurence/Promoter	p value
GGGCCTTA	-103	298	18.4650	0.6437	14.2048	33.1843	0.0384	0.00E+00
ATTGGGCC	-80	371	16.4096	0.6310	17.3609	49.4156	0.0826	0.00E+00
AAGGCCCA	-80	300	16.3496	0.6199	20.8945	45.9412	0.0950	0.00E+00
AGGCCCAT	-90	252	17.4686	0.6119	28.0038	57.9533	0.1025	0.00E+00
GCCCAATA	-90	356	15.2756	0.5986	17.3894	48.2310	0.0852	0.00E+00
AGGCCCAA	-80	334	14.1240	0.5919	18.1446	42.3106	0.0927	0.00E+00
GCCCATTA	-93	279	14.8587	0.5822	18.0117	40.9210	0.0929	0.00E+00
TAGGCCCA	-82	247	17.2682	0.5799	23.0668	48.2200	0.0747	0.00E+00
AAAGGCCC	-81	258	16.2846	0.5770	17.9627	40.0620	0.0479	0.00E+00
GGGCCTAA	-82	245	17.2903	0.5709	16.0519	38.5284	0.0393	0.00E+00
AATGGGCC	-79	275	15.2871	0.5688	20.1619	47.8438	0.1003	0.00E+00
ATAGGCCC	-71	244	16.1347	0.5553	14.9064	31.5541	0.0286	0.00E+00
CTGGGCCC	-128	223	14.8788	0.5379	5.3357	11.6752	0.0053	3.42E-10
GGCCCATA	-81	263	12.5368	0.5337	16.9959	36.4652	0.0567	0.00E+00
GGGCTTTA	-98	290	10.6136	0.5282	11.7583	21.4680	0.0489	0.00E+00
CACGTGTC	-77	316	13.5516	0.5269	12.3139	35.0861	0.0714	0.00E+00
CACGTGGC	-82	259	14.4412	0.5228	14.8337	33.8016	0.0492	0.00E+00
ACGGCCCA	-105	278	11.9923	0.5178	7.9898	17.8054	0.0223	9.66E-15
ACGCTGCG	-70	203	12.8595	0.5161	6.8921	10.1996	0.0080	3.55E-11
CAGGCCCA	-96	230	12.6120	0.5153	14.9110	32.9893	0.0329	0.00E+00
ACGTGGCG	-81	257	13.6389	0.5118	8.9350	22.8559	0.0261	3.22E-15
AATGGGCT	-99	308	10.6602	0.5102	13.0596	29.1191	0.0797	0.00E+00
GGCCCAAA	-78	344	10.1085	0.5094	9.5471	24.0658	0.0635	0.00E+00
GGGCCTGA	-87	203	13.2703	0.5093	10.9975	22.2116	0.0161	1.99E-13
CCGACCCG	-58	278	17.2973	0.5059	8.2127	28.4816	0.0252	5.44E-15
AAAGCCCA	-84	316	10.7514	0.5053	13.7856	32.1927	0.1222	0.00E+00
AACGGCCC	-62	177	14.5165	0.5032	10.5336	16.2729	0.0106	6.61E-12
AAGCCCAT	-93	316	10.2734	0.4985	12.2744	30.4228	0.1038	0.00E+00
ACGTGGCA	-82	252	10.8697	0.4981	13.9919	27.3572	0.0494	0.00E+00
ATGGGCCG	-83	312	11.5504	0.4959	7.4995	21.5063	0.0328	0.00E+00
AGCGCGTG	-55	259	12.2860	0.4955	7.1226	17.4813	0.0163	2.99E-13
ACACGTGG	-81	326	11.9162	0.4954	8.2498	24.7649	0.0521	0.00E+00
CGACCCGA	-60	286	16.3527	0.4928	5.8669	21.3718	0.0179	1.58E-13
ACTGGGCC	-65	232	11.3183	0.4877	10.5519	18.3263	0.0146	9.61E-13
ACACGCGC	-54	251	10.4351	0.4872	7.4824	15.5478	0.0128	2.78E-12
AAATGGGC	-85	326	9.3771	0.4869	14.6500	34.1225	0.0714	0.00E+00
GGGCCCTA	-85	199	15.4820	0.4863	6.2952	15.1422	0.0064	4.34E-10
GACGTGGC	-69	229	10.0490	0.4822	10.5517	20.7592	0.0288	4.44E-15
ACGTGTCA	-86	318	9.5575	0.4796	8.0686	19.9326	0.0554	0.00E+00
GACCCGAC	-65	246	17.2786	0.4796	7.7870	26.2249	0.0156	7.96E-13
ATGGGCCC	-68	206	12.5747	0.4783	10.0370	21.3913	0.0251	1.70E-14

GGGCCCAA	-100	203	10.8403	0.4766	10.6659	16.6789	0.0246	2.16E-14
TCGGCCCA	-86	227	12.3040	0.4645	12.0574	28.1072	0.0307	5.44E-15
GCCCATAA	-81	300	9.0472	0.4635	8.6707	21.0741	0.0432	0.00E+00
GCGCGTGA	-69	288	9.8942	0.4604	5.4185	13.6279	0.0168	9.14E-13
CCCATTAA	-106	330	8.5006	0.4519	9.8776	24.1562	0.0694	0.00E+00
GGCCCAGA	-81	144	12.9832	0.4509	7.9306	14.6435	0.0105	4.73E-11
CAAGGCCC	-96	206	9.1590	0.4508	7.2267	13.1992	0.0156	2.45E-12
GCCCGTTA	-104	165	11.5620	0.4497	6.5632	11.1610	0.0093	1.20E-10
AGGGCCCA	-86	264	9.6573	0.4496	7.1325	14.5284	0.0139	6.12E-12
AACGGGCC	-74	163	10.4191	0.4492	7.1222	10.8172	0.0107	4.44E-11
AGCCCAAT	-82	281	9.7915	0.4484	13.5044	31.1011	0.0719	0.00E+00
GATGGGCC	-104	288	9.2878	0.4457	5.8150	15.1024	0.0246	7.89E-14
CCCGGCCC	-69	175	12.1371	0.4456	5.6427	8.7794	0.0070	9.56E-10
ACCCGACC	-63	188	15.4670	0.4437	8.4236	25.1802	0.0213	2.74E-13
GGTCCCAC	-104	258	9.8638	0.4429	6.2715	14.1209	0.0120	2.40E-11
AAGCCCAA	-88	304	9.0608	0.4425	15.2100	38.3160	0.1091	0.00E+00
CACGTGGA	-109	261	7.6959	0.4363	9.2680	16.8374	0.0384	2.78E-15
AAAAGCCC	-88	275	9.3011	0.4338	10.7205	25.2371	0.0669	0.00E+00
AGGCCCAG	-81	159	10.6258	0.4319	9.0507	15.0679	0.0142	1.04E-11
AGAGGCCC	-73	188	12.5897	0.4317	6.1775	13.4060	0.0091	2.65E-10
CCGGTTTA	-66	286	7.7878	0.4301	13.2783	25.5086	0.0739	0.00E+00
GCCACGTA	-87	254	8.5391	0.4293	7.8307	16.8367	0.0217	4.49E-13
CGGCCCAA	-105	189	10.4066	0.4289	9.8758	18.2214	0.0283	5.23E-14
AAACGACG	-64	328	9.8813	0.4276	8.1944	26.2406	0.0564	0.00E+00
GGCCTTAA	-72	193	9.5767	0.4255	10.7132	17.4850	0.0260	1.23E-13
CCCAATAA	-82	337	8.1729	0.4245	10.0492	24.5515	0.0722	0.00E+00
AATTGGGC	-80	244	8.7357	0.4245	10.2530	19.8063	0.0512	0.00E+00
CCACGTCA	-129	286	7.2060	0.4219	8.4729	18.1013	0.0417	2.66E-15
TGGCCCAA	-72	289	8.0934	0.4214	7.7246	18.0823	0.0429	2.11E-15
ATTTGGGC	-103	283	9.4927	0.4214	10.0656	21.9800	0.0532	0.00E+00
TGGGCCCA	-68	182	10.6655	0.4208	11.8754	19.9816	0.0373	7.44E-15
CCGGTTCG	-53	239	8.5212	0.4134	6.1233	12.2708	0.0231	5.40E-13
AATAGGCC	-108	275	8.2620	0.4130	5.4694	13.4992	0.0205	1.43E-12
ATAAGGCC	-82	202	9.3623	0.4127	7.1761	14.2921	0.0244	3.68E-13
TAAGCCCA	-87	238	8.7137	0.4113	11.8966	24.8977	0.0646	0.00E+00
GGGCCGAA	-90	123	14.2248	0.4101	14.1424	21.6449	0.0147	2.04E-11
ACGACACG	-127	213	9.7860	0.4100	7.6466	13.3878	0.0175	5.39E-12
ACGGGCCT	-71	203	10.2292	0.4067	5.3599	8.5413	0.0072	3.16E-09
GGCCTAAA	-85	299	8.6554	0.4061	5.9431	16.9547	0.0295	1.05E-13
ACGACGTC	-59	203	11.3438	0.4046	11.0660	24.9767	0.0401	8.77E-15
ATATGGGC	-82	210	8.6393	0.4037	14.6098	26.3010	0.0521	9.99E-16
ACAGGCCC	-82	186	9.9741	0.4016	6.0933	13.0333	0.0126	8.80E-11
GCCCAGTA	-84	215	8.0163	0.4015	5.3148	9.9125	0.0119	1.36E-10
GGGCCTAC	-106	196	13.9349	0.4007	5.3304	10.9466	0.0106	3.12E-10
AAACCGGT	-85	264	6.6107	0.4005	8.3009	15.2198	0.0672	0.00E+00
ATGGCCCA	-108	290	7.2507	0.3987	7.5888	15.3867	0.0346	4.05E-14

ATGACACG	-95	250	6.5922	0.3968	7.6961	11.3641	0.0262	4.28E-13
AACGACGC	-65	196	12.3522	0.3945	7.2315	18.5394	0.0151	3.22E-11
CACGTCAG	-71	283	6.8792	0.3937	5.1616	11.1281	0.0207	3.09E-12
AACCGCGT	-72	240	9.5135	0.3908	5.5499	13.2217	0.0120	1.95E-10
CCCGACCC	-62	131	14.8592	0.3895	9.8496	22.2834	0.0190	7.14E-12
CCCATTTA	-87	333	6.4107	0.3840	6.4708	15.2218	0.0611	0.00E+00
CGTGGCAC	-83	186	9.2232	0.3832	6.6385	10.8801	0.0125	1.99E-10
GTGGCCCA	-80	160	10.6185	0.3807	6.2446	11.6159	0.0151	5.85E-11
ATTGGCCC	-106	231	6.5964	0.3796	6.2714	11.3959	0.0202	7.21E-12
GAGGCCCA	-91	186	9.4750	0.3795	6.2918	13.6582	0.0244	1.68E-12
ATGCCACG	-95	278	6.3732	0.3784	5.6195	12.0656	0.0205	6.79E-12
GGCCCAAC	-69	217	9.1938	0.3769	7.1557	16.2182	0.0250	1.62E-12
ACGTGGCT	-87	211	8.2416	0.3762	7.4959	14.5277	0.0243	2.04E-12
ACCGAACC	-74	333	6.4684	0.3760	6.0732	15.7985	0.0486	7.33E-15
CGTGGCAA	-71	176	8.1882	0.3730	8.0020	12.8838	0.0213	6.58E-12
CACGTGTA	-77	254	7.6719	0.3719	7.4802	17.0883	0.0418	3.24E-14
GCCCAGCC	-103	191	9.7421	0.3717	5.5386	9.9737	0.0075	9.79E-09
AACCGGGT	-113	204	7.9285	0.3708	6.0790	11.1018	0.0220	5.68E-12
CCGAACCG	-59	275	7.2581	0.3683	7.0756	16.0775	0.0314	4.00E-13
CCGGCCCA	-75	186	8.4716	0.3683	6.7358	12.6760	0.0171	4.21E-11
CTGGGCCA	-86	156	10.3296	0.3668	7.4470	14.8904	0.0090	3.65E-09
AAAAGGCC	-85	263	6.3434	0.3667	6.7974	13.2813	0.0476	1.42E-14
CACACGTG	-98	161	8.3418	0.3660	9.1335	15.7359	0.0287	9.16E-13
CATTGGGC	-136	169	7.0539	0.3657	11.9010	15.1028	0.0235	4.37E-12
ATAAGCCC	-85	220	8.4915	0.3653	8.6917	20.0927	0.0319	4.12E-13
ACCGGGTC	-51	191	7.9536	0.3651	5.5728	9.5623	0.0113	8.69E-10
CACGTGGG	-126	177	8.0681	0.3637	6.4443	11.1853	0.0148	1.48E-10
CCACGTCG	-96	162	8.3932	0.3628	5.7459	11.1361	0.0088	4.96E-09
CACGTCAC	-72	235	8.0364	0.3612	5.5870	13.1048	0.0223	8.15E-12
GGCCTTTA	-69	166	8.4858	0.3589	8.2751	13.6504	0.0266	2.41E-12
CGTGGCAG	-105	189	7.9328	0.3586	5.6612	10.2285	0.0149	1.76E-10
AGCCCAAA	-97	259	6.1976	0.3568	10.4163	19.4478	0.0702	8.88E-16
ACGTGTCT	-79	245	7.5919	0.3567	5.8877	14.0310	0.0329	5.06E-13
CGTGGCGA	-82	150	9.8495	0.3541	7.0881	13.0960	0.0122	8.60E-10
ACGTGTCC	-71	220	6.7608	0.3528	7.7976	15.2988	0.0190	3.98E-11
CGGTTTAA	-70	256	5.8338	0.3523	9.8193	18.2732	0.0654	2.11E-15
GCCCAAAC	-67	238	7.0068	0.3514	6.4061	15.4901	0.0305	1.21E-12
CTAGGCCC	-113	131	11.0664	0.3511	7.4576	13.7016	0.0105	2.55E-09
GAAGCCCA	-98	210	7.4659	0.3505	9.9204	19.8129	0.0365	3.04E-13
AGCCCATA	-94	199	7.4008	0.3502	8.3705	16.7547	0.0491	2.71E-14
ACGTGTCG	-95	150	8.8412	0.3476	10.4934	14.9593	0.0221	1.68E-11
GGGCCATA	-94	194	8.9544	0.3459	5.9782	11.6864	0.0172	1.16E-10
CCGGTTCA	-105	210	7.6068	0.3459	7.7467	15.6023	0.0332	8.27E-13
GACACGTC	-77	232	7.0527	0.3451	5.6871	10.4310	0.0184	7.31E-11
AGGCCCAC	-111	168	7.6098	0.3433	8.5134	13.8686	0.0176	1.10E-10
ATGACGTG	-60	232	6.2301	0.3415	6.6643	12.7236	0.0335	9.76E-13

TGGGCCAA	-105	218	6.2804	0.3415	8.4610	14.7352	0.0442	1.05E-13
TGAGCCCA	-88	205	7.1126	0.3414	6.5691	13.7899	0.0237	1.37E-11
CACGCGCC	-63	196	7.1061	0.3412	7.0103	12.3327	0.0167	1.76E-10
GGCCTATA	-112	205	7.2606	0.3368	5.9979	11.3043	0.0200	6.13E-11
CCGCGTTA	-69	116	12.7349	0.3360	5.3101	10.2984	0.0046	6.93E-07
ACGTCACG	-154	178	6.9716	0.3354	5.7850	8.6674	0.0118	2.45E-09
ATGGGCCA	-112	218	6.4158	0.3345	9.7610	18.2332	0.0432	1.89E-13
CGGACCCG	-104	114	11.6905	0.3324	5.5184	11.1730	0.0040	1.69E-06
AAGCGCGT	-56	136	8.9350	0.3322	6.8590	11.4246	0.0134	1.26E-09
AACGACGT	-66	205	8.7341	0.3314	9.3366	21.4808	0.0399	4.21E-13
CCGGGTCG	-82	85	14.6472	0.3314	11.9879	19.1701	0.0110	4.65E-09
CCACGTAG	-75	162	7.4734	0.3287	7.0748	13.9515	0.0138	1.19E-09
CAAACCGG	-69	195	6.7482	0.3282	8.9175	16.6800	0.0412	3.92E-13
AACGGCGT	-63	192	9.7478	0.3282	5.8694	14.4962	0.0173	2.58E-10
CGTGTCAC	-89	188	5.1028	0.3275	6.8501	9.0096	0.0187	1.57E-10
GGGCCGTA	-122	112	10.1867	0.3275	6.8334	10.8026	0.0089	2.11E-08
AAACGGCA	-67	198	6.1447	0.3265	7.5454	12.4238	0.0290	6.73E-12
CCGGTTAA	-67	197	7.5565	0.3255	8.2361	16.2002	0.0330	2.63E-12
CCGCGTGA	-134	146	7.9675	0.3237	5.7509	7.8019	0.0066	1.53E-07
ACCGGTTA	-82	177	7.5223	0.3231	8.6845	17.4548	0.0321	3.66E-12
CATGGGCC	-103	136	9.6170	0.3230	8.8511	16.0561	0.0220	6.19E-11
CTTGGGCC	-106	199	6.7880	0.3216	6.9767	15.0821	0.0175	3.41E-10
ATTAGGCC	-90	162	6.7195	0.3213	7.6366	11.4142	0.0249	2.75E-11
CCATGGGC	-108	166	10.8236	0.3196	5.5173	13.5920	0.0129	2.87E-09
ACGCCACG	-78	151	9.5032	0.3195	5.9094	12.1219	0.0134	2.22E-09
ATATTGGG	-87	274	5.7475	0.3193	6.6247	13.5804	0.0481	1.90E-13
AGCCCACT	-68	172	7.0903	0.3191	6.0239	9.6890	0.0201	1.46E-10
CGGTTTAG	-126	260	4.7881	0.3163	7.3540	11.4800	0.0515	1.32E-13
CGCGCGAG	-67	80	15.6702	0.3149	5.2647	9.1661	0.0026	2.84E-05
ACGTGGAC	-91	192	6.2096	0.3144	6.6095	11.9398	0.0234	6.26E-11
TAAACCGA	-73	327	4.3445	0.3136	5.5643	10.9422	0.0747	6.88E-15
ACGTCAGC	-68	200	7.2741	0.3131	5.7744	12.5696	0.0181	4.06E-10
AACCCGGT	-98	170	7.6491	0.3119	7.1060	12.9531	0.0164	8.54E-10
ACACGTCA	-69	207	6.4363	0.3113	7.7169	13.9235	0.0311	9.20E-12
AAGCCCAC	-97	200	7.2047	0.3096	6.5709	14.9736	0.0302	1.25E-11
CGGTTTAC	-97	220	6.2949	0.3090	7.4918	13.8430	0.0309	1.08E-11
AAAACGAC	-62	376	4.6161	0.3084	6.2710	15.8081	0.0787	6.22E-15
AAACCGGA	-65	206	6.1901	0.3084	10.5886	18.7524	0.0702	1.64E-14
ACGGCACG	-65	106	9.9269	0.3069	7.3851	11.5161	0.0075	1.49E-07
GGACCCAC	-66	162	6.0578	0.3053	5.8501	9.4057	0.0155	1.73E-09
AAACGGCG	-63	185	7.2876	0.3047	5.2904	10.7508	0.0186	5.18E-10
CAAGCCCA	-90	217	5.5692	0.3036	8.2697	16.6391	0.0383	2.88E-12
ACGTCGTC	-63	185	8.0416	0.3029	8.0141	18.5603	0.0231	1.27E-10
AACGACAC	-80	222	6.6243	0.3016	5.5932	13.0554	0.0265	5.08E-11
ACCGGTTC	-62	215	5.4573	0.3000	6.4459	11.7444	0.0340	8.86E-12
GCCCAAAA	-136	236	4.7116	0.2996	10.0485	16.0121	0.0639	6.26E-14

GGCCTTAC	-65	146	7.4867	0.2984	5.0064	9.4948	0.0083	1.21E-07
AAAACGGC	-66	185	5.8150	0.2984	6.6724	11.1308	0.0298	2.59E-11
GCCCACTA	-69	134	9.9501	0.2967	6.6089	12.4882	0.0165	1.82E-09
GAGCCCAA	-84	239	4.9848	0.2944	6.4111	11.5923	0.0242	1.46E-10
AACCGGAA	-64	197	6.2307	0.2926	9.1743	18.3724	0.0563	2.77E-13
GGCCTAAC	-84	182	5.9636	0.2916	5.3214	8.4778	0.0139	7.21E-09
ACACGCGT	-132	137	6.3238	0.2914	6.3893	7.8009	0.0127	1.29E-08
ACGACGCC	-67	131	10.5120	0.2913	7.1535	14.8238	0.0145	5.45E-09
ACGCGCCG	-75	140	9.6908	0.2886	5.5508	11.5810	0.0076	3.20E-07
CGGGTCGA	-62	130	8.1340	0.2865	5.7157	10.0445	0.0079	2.81E-07
ACGGCGTC	-62	190	7.9034	0.2862	5.4059	13.8330	0.0157	4.26E-09
CACGTGCG	-61	121	10.1887	0.2850	5.4244	10.1316	0.0116	3.00E-08
AAAACCGG	-105	206	4.8952	0.2840	8.2360	13.6053	0.0442	3.23E-12
GCCCAACA	-85	202	5.1915	0.2828	5.7998	9.7573	0.0257	1.84E-10
ACCACGTG	-86	153	6.8887	0.2825	6.2825	12.4416	0.0202	9.98E-10
GGCTTTAA	-111	276	4.8295	0.2821	5.4053	12.0391	0.0458	2.75E-12
ATAATGGG	-108	202	6.7545	0.2816	6.0796	14.9855	0.0494	1.58E-12
ACGTGGAT	-145	197	5.5661	0.2810	5.1020	10.3619	0.0300	6.90E-11
ATAACCGG	-84	145	5.6389	0.2806	7.5288	11.0609	0.0231	4.47E-10
GGCTTTTA	-97	277	4.8669	0.2789	5.3089	11.5981	0.0550	8.13E-13
CCAAACCG	-69	240	4.5898	0.2778	6.1406	10.9437	0.0461	3.46E-12
CAATGGGC	-76	152	5.4426	0.2777	6.8481	10.5891	0.0240	4.02E-10
AACCGGTC	-55	161	5.5822	0.2774	6.2961	9.6200	0.0232	5.14E-10
CAACGGTC	-82	201	6.3927	0.2769	5.4622	12.3786	0.0254	2.82E-10
CCGTTGGA	-72	204	5.4911	0.2759	5.7728	11.1301	0.0331	4.54E-11
ATCGGCCC	-83	102	8.7888	0.2758	7.3673	10.2900	0.0108	7.44E-08
AACCGAAC	-61	244	4.5737	0.2740	7.1001	12.8920	0.0627	3.97E-13
GACACGTA	-105	193	6.5033	0.2727	5.0115	11.5556	0.0214	1.17E-09
CCCATTAG	-91	149	7.0319	0.2709	7.5677	13.3407	0.0307	1.09E-10
CAAAGCCC	-85	206	6.1541	0.2701	5.0429	11.2709	0.0295	1.50E-10
CCGCCACG	-71	108	9.3302	0.2690	6.8394	12.1078	0.0101	1.56E-07
AACCGGAT	-69	170	6.2054	0.2686	8.1822	14.3916	0.0425	1.16E-11
ACGTGTAC	-67	202	5.1169	0.2682	5.1321	9.2745	0.0229	9.52E-10
CTTAGGCC	-81	150	7.8132	0.2672	5.1204	12.5613	0.0116	7.48E-08
GCGTTTTA	-124	212	3.8535	0.2655	5.5395	7.1533	0.0297	1.87E-10
GTCGTTTA	-72	218	5.5290	0.2652	6.6871	14.2565	0.0389	2.78E-11
AAAACGCG	-54	169	4.9359	0.2635	5.6532	8.1059	0.0219	1.69E-09
AATACCCT	-78	158	6.9893	0.2629	6.2019	12.9776	0.0236	1.08E-09
AAACCGAA	-165	270	3.6225	0.2626	7.2636	11.7092	0.1137	6.55E-15
AAACGACA	-86	224	4.5839	0.2626	9.8904	16.3589	0.0716	3.05E-13
TAAGGGTA	-83	170	5.7567	0.2619	6.3605	10.0314	0.0234	1.21E-09
TGGGCTAA	-108	175	5.4575	0.2611	5.8850	9.7407	0.0306	2.01E-10
AGATGGGC	-109	168	6.4573	0.2585	5.9786	10.7939	0.0203	3.79E-09
AACCGGAC	-102	143	6.0647	0.2555	6.6609	10.7837	0.0214	3.19E-09
AGCCCAAC	-126	140	5.4254	0.2545	6.0765	8.7163	0.0257	1.02E-09
CGAACCGA	-76	200	5.6085	0.2544	5.5224	11.0807	0.0317	2.43E-10

GAAACGAC	-68	192	6.2779	0.2542	6.0561	13.3725	0.0319	2.32E-10
CTAAGGCC	-94	137	5.8027	0.2540	5.5683	8.7223	0.0111	1.96E-07
ACGTCATC	-57	167	5.6025	0.2540	6.2051	11.4975	0.0300	3.62E-10
GAACCGGA	-70	192	4.8723	0.2525	6.6349	11.1290	0.0337	1.75E-10
AACCCGAC	-63	150	5.8522	0.2491	6.0676	11.2352	0.0155	3.54E-08
ATGGGCTA	-111	141	6.5372	0.2489	5.4668	10.0805	0.0267	1.11E-09
CCGACTTA	-59	121	6.5539	0.2485	5.6291	9.1368	0.0091	7.88E-07
CACGTGAC	-142	159	5.0137	0.2480	6.2623	9.9696	0.0278	8.92E-10
AATAGCCC	-119	145	5.8107	0.2458	5.1512	9.2518	0.0148	5.77E-08
CAAAACGC	-75	193	3.9393	0.2438	6.0679	8.3585	0.0350	2.43E-10
ATCCAACG	-74	195	5.0874	0.2432	5.7859	11.3091	0.0386	1.26E-10
GGGCCTCA	-84	95	7.9745	0.2431	6.1425	9.4218	0.0130	1.43E-07
ACGTGTGA	-77	145	5.0749	0.2429	6.3382	8.8135	0.0269	1.56E-09
AAAAAGCC	-96	217	3.7248	0.2428	6.2969	9.0523	0.0659	2.48E-12
AACACGTG	-72	152	5.1460	0.2425	8.7678	13.7998	0.0441	5.11E-11
CCCATGGG	-74	164	5.6356	0.2419	5.4189	10.9466	0.0133	1.35E-07
GGGTCAAA	-87	242	4.4852	0.2413	5.2706	10.4419	0.0415	8.65E-11
CCCGGGTC	-84	87	13.7921	0.2390	7.2078	12.3163	0.0060	1.11E-05
CCGTCCGA	-79	112	6.7925	0.2372	6.1499	9.8343	0.0098	9.71E-07
ACCCCTGA	-73	88	9.4284	0.2353	5.7746	10.7229	0.0069	6.60E-06
GGGTCCCA	-83	101	6.6803	0.2326	6.0041	9.0221	0.0130	2.57E-07
GACCCGAA	-69	162	6.5686	0.2321	5.2908	12.7329	0.0180	3.88E-08
ATAAACCG	-126	184	4.1998	0.2309	6.4134	10.4862	0.0502	4.58E-11
ACGACACC	-78	128	6.7436	0.2308	5.1722	10.5528	0.0154	1.09E-07
CCCAATTA	-83	138	5.1538	0.2289	10.1469	15.6768	0.0495	5.87E-11
ATAAAGCC	-93	195	4.0068	0.2287	5.4626	8.8770	0.0410	2.28E-10
AAATACCC	-60	153	5.0394	0.2282	7.3771	12.6708	0.0340	8.53E-10
ACCGTTAG	-104	136	6.5218	0.2282	6.3685	11.9198	0.0162	9.38E-08
CCATTAAG	-140	183	4.3095	0.2239	5.0449	8.4119	0.0342	1.09E-09
CGGTTAAG	-65	153	5.5702	0.2218	5.9882	11.1289	0.0244	1.16E-08
ACGTGACA	-64	231	4.6020	0.2200	5.0345	11.5936	0.0276	5.96E-09
AGTTGGGC	-70	114	5.3699	0.2196	6.4476	9.1116	0.0207	3.62E-08
AAGCCACG	-89	114	6.1870	0.2169	6.2105	10.5249	0.0189	7.48E-08
TCAGCCCA	-73	141	4.9059	0.2168	6.5877	10.7949	0.0210	3.99E-08
CCGTTAAA	-104	173	4.4523	0.2166	5.7796	9.5563	0.0283	6.31E-09
CCAATAAG	-84	173	5.4772	0.2154	5.2969	11.2430	0.0419	5.14E-10
GGCCCACA	-110	128	6.3202	0.2148	7.0143	11.6736	0.0147	3.65E-07
GCCGTTTA	-102	129	4.7212	0.2139	5.5754	8.0024	0.0175	1.43E-07
AATACCCC	-81	110	5.5870	0.2139	5.1852	7.8021	0.0132	7.11E-07
GGGACCCA	-79	107	6.5518	0.2132	6.6940	10.3113	0.0139	5.49E-07
ATAAGGGC	-109	87	7.2526	0.2114	5.2354	7.3643	0.0079	1.13E-05
TCGGGTCA	-81	91	7.2805	0.2110	7.8178	12.4318	0.0166	2.31E-07
CACCCCAC	-187	86	8.2138	0.2103	5.1704	8.5325	0.0058	5.15E-05
CCAGGCCC	-84	84	9.3941	0.2098	6.2122	10.0464	0.0101	3.74E-06
CCCATATA	-112	159	4.6417	0.2074	6.0499	11.7118	0.0395	1.40E-09
TAACCGGA	-66	122	4.8218	0.2067	7.7495	11.6213	0.0319	5.94E-09

CCGTCAGA	-102	133	5.0299	0.2063	5.3833	10.0409	0.0109	3.08E-06
AAGGGTAT	-81	148	5.1081	0.2054	6.1858	12.1760	0.0303	9.08E-09
CCCAATAG	-88	122	4.7404	0.2052	7.2840	9.8199	0.0271	1.86E-08
ACCGTCAG	-102	97	5.7275	0.2038	5.2776	8.3032	0.0077	1.94E-05
AAACGCTG	-59	140	5.3370	0.2025	5.2397	9.0773	0.0198	1.45E-07
ACGTGGAA	-83	174	4.3108	0.1985	6.0460	10.5589	0.0330	8.79E-09
ACACGTGA	-92	154	4.4221	0.1959	6.7218	11.6944	0.0321	1.27E-08
CCGGTATA	-95	95	6.5583	0.1956	5.2184	9.0054	0.0108	5.97E-06
CGACATCG	-69	114	6.8776	0.1915	5.2724	11.3528	0.0164	8.94E-07
CGCGTGAA	-82	87	6.9955	0.1911	5.4776	8.3850	0.0127	3.57E-06
AAAGTCAA	-68	261	3.0594	0.1890	6.2197	10.9888	0.1363	8.12E-13
AAACGCGT	-109	130	4.2457	0.1875	6.6285	8.0096	0.0224	2.04E-07
CTAAGCCC	-88	95	6.3879	0.1867	7.0985	11.4577	0.0171	9.62E-07
GCCCATCA	-72	111	4.1135	0.1846	5.9202	7.3059	0.0235	1.92E-07
ACCCCTTA	-69	132	5.7652	0.1842	5.0810	9.5846	0.0106	1.31E-05
GAACCGGC	-95	84	7.1366	0.1775	5.7461	9.9894	0.0100	2.60E-05
AGTCGGTC	-75	73	5.9991	0.1763	5.5391	8.0822	0.0091	4.41E-05
GCCTTTAA	-74	124	4.4144	0.1756	7.0183	11.0944	0.0309	7.56E-08
CGGTTCAA	-100	120	4.8651	0.1729	5.5855	9.7484	0.0383	2.59E-08
AACCGACT	-78	114	4.9145	0.1690	5.2262	9.1163	0.0207	1.21E-06
AGGGGTAA	-69	98	4.8813	0.1689	5.0398	8.0471	0.0152	6.07E-06
GAATGGGC	-72	103	5.1555	0.1667	5.0554	8.6847	0.0168	4.32E-06
ACCGGAAA	-56	165	3.6202	0.1657	5.6947	9.2576	0.0434	2.21E-08
CGGTTAAA	-129	122	4.2866	0.1601	5.9564	9.7033	0.0379	8.19E-08
CGTAATTA	-128	122	4.0991	0.1599	6.9141	10.0787	0.0434	3.69E-08
CAATTGGG	-134	134	3.9637	0.1563	5.1824	8.6405	0.0269	7.94E-07
AACGACGA	-61	125	3.7873	0.1552	5.2529	8.4569	0.0351	1.95E-07
AGGCCTAT	-114	73	5.8298	0.1536	6.2169	9.1370	0.0140	2.62E-05
AAAACGCC	-64	87	5.6362	0.1456	6.1854	10.4295	0.0228	4.51E-06
AAGCCCTA	-50	69	4.7440	0.1429	7.3652	9.6645	0.0194	1.27E-05
ACGGTTTA	-137	120	3.2218	0.1373	5.9428	7.5848	0.0349	9.97E-07
CGTTTTGA	-66	158	3.0402	0.1350	5.0143	7.5320	0.0551	9.34E-08
ATTGCCAC	-112	89	4.1760	0.1299	6.0026	10.3868	0.0235	1.46E-05
CCGAACCA	-57	86	4.0383	0.1261	6.8001	9.2086	0.0331	3.75E-06
CAAAGGCC	-86	67	4.4803	0.1164	5.3678	7.6178	0.0203	9.14E-05
AAATGACG	-71	80	4.1398	0.1122	7.4523	11.2446	0.0353	1.06E-05
ACCCTAGA	-52	67	3.8862	0.1076	5.3109	7.2004	0.0321	2.65E-05
