# Supplementary material for: Identification of plant promoter constituents by analysis of local distribution of short sequences
Source: BMC Genomics. 2007 Mar 8;8:67. doi: 10.1186/1471-2164-8-67 (PMC1832190; doi:10.1186/1471-2164-8-67)
Supplement: Additional file 6 — Rice REG octamers (Table S5.pdf). Contains octamer sequences and parameters. [file 1471-2164-8-67-S6.pdf]

Table S5. Rice REG octamers

Sequence	Peak Position	Peak Width	RPH	RPA	Peak Area/ basal fluctuation	(Peak hight-Base Line)/sd	Occurence /Promoter	p value
ATGGGCCG	-86	312	9.5691	0.4997	11.1578	25.9229	0.0753	0.00E+00
GGGCCCCA	-70	449	9.1829	0.4906	9.4650	29.6325	0.0849	0.00E+00
GGGCCCAC	-71	378	10.0566	0.4845	10.7327	31.2565	0.1305	0.00E+00
ACGGCCCA	-85	253	10.7343	0.4819	13.1354	27.2637	0.0640	0.00E+00
GGCCCACC	-73	250	10.5591	0.4719	16.1401	33.7931	0.0742	0.00E+00
CTGGGCCC	-61	307	9.0479	0.4665	7.0548	14.7945	0.0273	4.07E-14
GATGGGCC	-78	294	8.0941	0.4607	10.5834	21.3960	0.0638	0.00E+00
CCGGCCCA	-85	266	9.0745	0.4536	12.6195	26.2232	0.0840	0.00E+00
CGGGCCCA	-85	334	9.1441	0.4505	6.0350	15.7810	0.0388	4.11E-15
TCGGCCCA	-75	292	8.3622	0.4503	9.4088	20.0625	0.0589	0.00E+00
GGGGCCCA	-70	389	8.7349	0.4499	9.8401	29.2974	0.0906	0.00E+00
GGCCCCAC	-72	403	8.2847	0.4485	8.8793	25.3851	0.0996	0.00E+00
GGCCCAGA	-90	289	8.4131	0.4484	7.0873	15.8493	0.0321	2.25E-14
AGGCCCAT	-66	250	9.4723	0.4458	14.2430	29.1043	0.0635	0.00E+00
CGTGGGCC	-68	294	8.3459	0.4369	9.6627	20.0947	0.0713	0.00E+00
AATGGGCC	-63	252	8.7763	0.4362	11.4227	23.5933	0.0637	0.00E+00
CGGCCCAC	-74	301	9.3068	0.4322	8.0577	23.1001	0.0538	0.00E+00
GGCCCAAC	-69	285	8.2521	0.4287	8.7740	20.2909	0.0530	0.00E+00
ATCGGACG	-66	268	8.1317	0.4285	8.5271	18.0899	0.0381	1.31E-14
GGGCCGAA	-67	220	8.4655	0.4224	9.4786	17.4843	0.0325	6.53E-14
CGGCCCAA	-103	264	7.8725	0.4151	8.6900	18.9821	0.0731	0.00E+00
CGGCCCAG	-86	265	8.6589	0.4144	9.5431	21.7298	0.0495	2.66E-15
CCAGCCCA	-61	346	7.5201	0.4143	7.2539	18.8957	0.0660	0.00E+00
CCGTCCGA	-92	224	9.6099	0.4124	9.1205	20.5841	0.0326	1.01E-13
GCCCCACC	-62	384	6.2644	0.4116	7.3757	16.8211	0.0643	0.00E+00
TAGGCCCA	-81	265	8.1157	0.4089	8.1914	16.8400	0.0448	8.33E-15
AAGGCCCA	-68	221	6.8378	0.4016	10.2039	15.8047	0.0579	1.33E-15
GCCCAGCC	-63	231	9.2519	0.3961	8.7669	19.1588	0.0447	1.61E-14
GCGGGCCC	-69	326	7.3768	0.3952	5.7483	13.9576	0.0257	1.50E-12
CAGGTGGG	-88	380	7.0940	0.3951	5.7991	16.5136	0.0593	1.44E-15
ACCGGGCC	-97	284	6.6239	0.3949	5.1754	9.7807	0.0177	2.50E-11
GGGCCCAA	-92	272	8.6352	0.3859	7.3211	20.1302	0.0394	7.66E-14
GGCCCAAA	-95	278	8.3089	0.3803	8.4704	22.1356	0.0630	1.89E-15
GCCCATTA	-100	212	8.1707	0.3777	9.7387	18.8729	0.0366	2.10E-13
GGGCCGCA	-66	225	8.4395	0.3773	6.1041	14.0397	0.0215	1.32E-11
ATTGGGCC	-56	261	7.1047	0.3771	9.3810	20.2599	0.0516	1.27E-14
GGGCCGTA	-88	244	10.0286	0.3766	5.4167	15.5942	0.0181	4.95E-11
CTGGCCCA	-66	263	6.7392	0.3681	6.5691	14.5001	0.0401	1.62E-13
AGTGGGCC	-60	256	7.2921	0.3666	8.4096	17.2878	0.0472	4.63E-14
AGATGGGC	-105	267	6.0480	0.3577	7.9915	14.8887	0.0424	1.78E-13
AAAGCCCA	-75	223	5.9118	0.3569	8.8513	15.5155	0.0568	1.64E-14
GATCCGAC	-77	201	8.5917	0.3533	6.3247	13.0565	0.0220	3.51E-11

ACTGGGCC	-87	170	8.9788	0.3516	12.2669	20.1104	0.0335	1.57E-12
CCCGGCCC	-68	201	7.2368	0.3480	8.6898	16.2875	0.0528	4.96E-14
AGGCCCAA	-111	214	7.2741	0.3463	10.1996	21.3652	0.0577	2.62E-14
CCGTGGGC	-72	194	6.8343	0.3462	8.3084	12.9382	0.0325	2.64E-12
GACGGCCC	-97	204	7.5838	0.3461	6.6505	14.0361	0.0282	7.88E-12
GGCCCATA	-97	177	7.1159	0.3460	12.9029	20.3364	0.0499	8.85E-14
CCCACGGG	-69	188	5.9452	0.3457	7.3902	10.6780	0.0176	2.41E-10
CCGGGCCC	-84	176	8.3503	0.3450	7.7474	14.3328	0.0296	5.81E-12
CACGTGTC	-71	263	7.4950	0.3430	6.4351	15.3614	0.0424	3.89E-13
GCTGGGCC	-80	177	7.9435	0.3407	7.6217	13.9915	0.0442	3.20E-13
GGGCCGGA	-79	183	7.4946	0.3389	9.8245	16.1681	0.0328	3.67E-12
GCGGCCCA	-66	190	6.3000	0.3375	9.0736	14.5754	0.0448	3.38E-13
CCCAGCCC	-58	196	8.1682	0.3367	7.3253	15.9403	0.0433	4.69E-13
CAGCCCAA	-133	308	4.7325	0.3351	6.0641	10.8513	0.0541	8.38E-14
CATGGGCC	-59	235	5.9515	0.3350	8.0284	15.7472	0.0603	3.46E-14
AGGTGGGC	-82	258	5.4189	0.3349	7.4441	13.9349	0.0518	1.23E-13
CCGTCGGA	-118	206	7.3377	0.3335	7.2353	15.3721	0.0294	1.09E-11
CTCGGCCC	-66	214	6.3842	0.3334	5.7102	10.8049	0.0333	4.28E-12
ACAGGTGG	-89	297	5.4121	0.3326	5.6671	11.9517	0.0536	1.05E-13
AAGCCCAC	-65	211	5.7765	0.3316	7.0823	11.8046	0.0336	4.46E-12
CACGGCCC	-63	248	5.5871	0.3306	6.0283	11.2610	0.0482	2.81E-13
GACGTGGC	-77	449	3.3797	0.3292	5.3075	9.4234	0.0768	6.33E-15
ATGGCCCA	-88	222	5.5779	0.3282	5.5683	9.6739	0.0422	9.11E-13
GGCCCACA	-73	203	7.5416	0.3279	8.8310	18.5610	0.0686	1.78E-14
GCCCACCA	-77	252	5.7735	0.3268	7.6896	15.6349	0.0486	3.24E-13
GCCCAGTA	-55	157	7.7601	0.3263	6.9746	11.6694	0.0189	3.88E-10
ATCCGACG	-80	168	7.1949	0.3259	7.0243	11.1201	0.0311	1.07E-11
CAGGCCCA	-79	223	7.0679	0.3240	6.8430	16.4542	0.0420	1.19E-12
GCCCAATA	-72	186	6.6652	0.3213	7.0033	11.2714	0.0383	2.90E-12
CAAGGCCC	-58	210	6.6092	0.3203	5.8364	12.5051	0.0284	2.87E-11
CCCACGCG	-69	262	4.9478	0.3198	6.1785	11.2984	0.0392	2.58E-12
CCCCACAC	-50	249	5.7999	0.3191	6.1013	13.1789	0.0336	8.75E-12
CCCACCCG	-106	224	6.1907	0.3188	7.3248	13.4259	0.0358	5.57E-12
GCCCACCC	-58	228	5.4386	0.3184	8.1290	13.4538	0.0373	4.09E-12
AGGCCCAC	-72	186	6.5972	0.3183	8.2189	14.9439	0.0376	3.95E-12
GATCGGAC	-65	183	6.9511	0.3179	7.6278	13.9263	0.0306	1.90E-11
GAGGCCCA	-93	210	5.7632	0.3162	8.9078	15.3577	0.0487	5.91E-13
GTGGGCCA	-61	211	5.8692	0.3146	6.8331	11.6704	0.0394	3.39E-12
TGGCCCAA	-121	269	5.1494	0.3047	5.2529	11.2371	0.0463	1.71E-12
CCGTTGGA	-104	152	6.1995	0.3033	8.4426	12.2485	0.0347	1.66E-11
GCCCACAA	-72	214	6.0860	0.3031	7.3919	13.4507	0.0313	3.64E-11
AAAAGCCC	-76	165	6.2222	0.3023	8.5883	14.8781	0.0411	4.99E-12
ACGTGGCG	-103	315	4.5149	0.3019	6.8347	14.0146	0.0687	8.74E-14
CTTGGGCC	-99	253	5.2329	0.3013	5.9747	13.0217	0.0488	1.39E-12
AGCCCACA	-57	218	5.8340	0.3012	7.0145	11.4740	0.0359	1.45E-11
AACGGCCC	-74	164	6.7220	0.3004	5.8873	10.7751	0.0209	7.26E-10

AACGGGCC	-112	168	6.3054	0.2984	7.1424	11.9177	0.0216	6.43E-10
GCCCATCC	-83	236	4.7943	0.2968	5.8777	9.7794	0.0363	1.75E-11
CCTGTCAG	-152	165	6.5686	0.2967	8.0013	13.8317	0.0295	7.96E-11
ATGGGCCA	-63	201	5.5795	0.2957	8.4856	15.3893	0.0543	8.58E-13
AGCCCATC	-73	229	5.6367	0.2943	5.5400	12.2138	0.0385	1.30E-11
CGTGGGGC	-57	235	5.5637	0.2927	5.2132	10.9455	0.0297	9.54E-11
GCCCCCAC	-51	248	4.4622	0.2925	6.6129	10.3170	0.0405	9.94E-12
ATCTGGGC	-88	155	6.4429	0.2925	6.3231	9.6481	0.0230	5.72E-10
AGTTGGGC	-109	193	6.4490	0.2906	6.8478	14.5081	0.0384	1.65E-11
CGGGCCCC	-78	174	7.1733	0.2905	5.5829	11.0888	0.0249	3.72E-10
ATGGGCCC	-69	199	6.2403	0.2899	6.5232	12.0050	0.0446	5.57E-12
CCAACGGC	-68	165	6.7675	0.2887	9.3601	16.4364	0.0356	3.25E-11
AGCCGTCC	-85	168	6.5394	0.2885	7.8157	13.5163	0.0231	6.82E-10
GCCCAAAA	-97	314	4.8782	0.2883	6.0204	14.4595	0.0560	1.08E-12
ACACGTGG	-74	234	5.6970	0.2882	5.7190	12.6123	0.0456	5.27E-12
ATGGGCTG	-90	215	5.8133	0.2861	6.9171	13.3956	0.0427	9.87E-12
CTGGGCCA	-82	149	6.0122	0.2860	8.5018	12.7022	0.0273	2.49E-10
AAGCCCAT	-70	181	5.5663	0.2845	7.3239	12.9855	0.0438	8.92E-12
CCAGGCCC	-64	201	6.2856	0.2842	5.1189	10.8768	0.0242	6.46E-10
AGGCCCAG	-90	220	6.2301	0.2830	5.2600	12.7062	0.0346	5.51E-11
CACCTGTC	-84	240	5.2390	0.2825	6.4165	12.5686	0.0496	3.96E-12
AAGCCCAA	-60	174	4.5094	0.2822	7.6302	9.8514	0.0529	2.46E-12
GAAGCCCA	-95	206	5.3973	0.2818	6.2752	12.1787	0.0360	4.50E-11
AGCCCACG	-95	215	5.2386	0.2778	5.2301	9.7524	0.0256	6.34E-10
AGCCCAAC	-72	178	4.5272	0.2776	6.8786	8.6662	0.0431	1.56E-11
GCCCACGC	-98	266	4.9277	0.2768	5.2899	11.5515	0.0443	1.33E-11
CCATGGGC	-79	172	5.3162	0.2761	7.4228	11.9408	0.0410	2.47E-11
TCAGCCCA	-98	225	4.7741	0.2742	6.4645	10.0310	0.0358	7.34E-11
ACAGCCCA	-90	217	4.8366	0.2724	6.2493	10.9566	0.0359	8.18E-11
GCCCACGA	-67	188	4.9025	0.2717	6.1217	10.0896	0.0309	2.43E-10
GTGGCCCA	-59	224	4.9848	0.2710	5.3169	9.7433	0.0388	5.04E-11
GCCCAACC	-88	170	5.6743	0.2699	6.4446	10.8822	0.0329	1.77E-10
CCCACCAC	-56	239	4.7156	0.2698	7.3053	14.4670	0.0779	2.65E-13
ACCCCCCA	-55	166	6.5370	0.2696	5.8104	11.2871	0.0247	1.28E-09
ACGCGTGG	-67	185	5.1619	0.2696	6.0781	9.7906	0.0275	6.27E-10
GCCCATCA	-65	207	5.3466	0.2692	6.3157	14.2070	0.0454	1.80E-11
CCGGGCCG	-101	203	5.6416	0.2682	6.8135	13.0463	0.0344	1.43E-10
AATTGGGC	-55	216	5.2068	0.2661	5.9959	10.7112	0.0350	1.43E-10
AGCCCAGC	-64	152	6.4988	0.2661	5.9743	11.0065	0.0341	1.71E-10
GCCCAAAC	-93	201	6.0656	0.2635	6.4546	15.8148	0.0363	1.30E-10
GGACGGAC	-91	193	5.7889	0.2631	5.9942	11.0449	0.0250	1.72E-09
GCCCATAA	-107	200	5.4148	0.2612	5.9836	12.6057	0.0314	4.11E-10
CACCGCAC	-128	199	4.4029	0.2560	6.1842	10.0791	0.0339	3.35E-10
GCCCGGCC	-69	205	6.0183	0.2558	8.2765	19.6972	0.0508	1.88E-11
GGCCGAAA	-122	186	4.6646	0.2537	6.7897	9.9757	0.0289	1.15E-09
ATCCAACG	-73	153	5.2343	0.2527	9.0406	13.6233	0.0457	5.04E-11

CTGGGGCC	-72	152	4.2820	0.2527	5.1419	6.0601	0.0115	3.68E-07
AATGGGCT	-86	192	4.7880	0.2523	6.9121	11.3289	0.0378	2.00E-10
CGCGTCGC	-54	285	3.9324	0.2521	5.4104	10.4202	0.0614	5.87E-12
CCCCCACG	-61	176	5.2938	0.2510	6.1938	11.1995	0.0302	1.02E-09
TAAGCCCA	-71	158	5.3635	0.2506	6.5310	10.2746	0.0240	4.72E-09
CGCACGCG	-50	214	3.9919	0.2494	6.8624	9.1372	0.0423	1.10E-10
GGCCCGGA	-107	146	7.1574	0.2472	5.0115	11.3490	0.0158	7.65E-08
AGCCCATG	-118	178	4.2053	0.2468	7.4287	10.6627	0.0362	3.89E-10
ACGTGGGC	-94	175	6.3550	0.2466	8.2071	16.1850	0.0429	1.19E-10
GGGCCAGA	-62	172	5.1875	0.2465	5.0294	8.6053	0.0221	1.02E-08
ACGTGTCC	-84	156	6.6955	0.2448	5.5031	12.2613	0.0256	4.48E-09
CACTGACA	-91	233	5.0619	0.2446	6.0582	12.9463	0.0536	2.75E-11
AAATGGGC	-62	193	4.5344	0.2445	8.2270	12.7146	0.0583	1.48E-11
AGCCCAAA	-97	173	4.3974	0.2438	8.3652	11.8436	0.0483	6.17E-11
AGCCCAAT	-57	205	4.5674	0.2432	5.0880	8.6406	0.0344	6.96E-10
CCTGGGCC	-91	163	5.1423	0.2417	6.5613	12.5299	0.0289	2.42E-09
GTCCCTCA	-188	186	3.4981	0.2414	5.6586	6.9868	0.0392	3.19E-10
CACGCCAC	-61	193	4.3944	0.2398	5.7502	9.1103	0.0408	2.67E-10
GCCCACAC	-55	211	5.3583	0.2398	5.0182	11.3221	0.0334	1.05E-09
ACGTGTCG	-68	166	5.5061	0.2390	5.8866	10.1910	0.0250	7.49E-09
CACGTGGC	-74	241	3.9875	0.2382	5.3883	10.2166	0.0620	1.48E-11
CCGATCCG	-80	173	4.7864	0.2374	5.5765	8.8979	0.0272	4.76E-09
GACTCGAC	-104	113	7.7089	0.2356	5.8592	8.6322	0.0118	7.64E-07
ACTGACAG	-77	157	4.5367	0.2333	5.3720	7.4847	0.0272	6.19E-09
TGGGCCAA	-80	160	5.4556	0.2327	8.4505	14.8695	0.0462	1.85E-10
AGCCCACC	-89	195	3.9244	0.2325	5.4024	8.2798	0.0359	1.05E-09
CCCACGAA	-55	135	6.0822	0.2315	5.1136	9.2339	0.0203	4.30E-08
ACGTGGGG	-60	153	6.5358	0.2310	5.3840	10.7436	0.0234	1.84E-08
AGGGCCCA	-72	193	4.4559	0.2304	5.5110	9.3573	0.0274	7.09E-09
GCCCAACA	-115	202	4.2234	0.2282	5.2698	8.7504	0.0444	3.35E-10
CGCGACGC	-51	176	3.9046	0.2278	10.1558	12.9439	0.0469	2.36E-10
CAACGGTC	-99	154	5.3792	0.2261	5.3643	10.2416	0.0175	1.45E-07
GACGCGAC	-126	167	4.1578	0.2261	5.8964	8.6381	0.0283	7.64E-09
CACGTGGG	-79	172	5.5690	0.2255	9.1820	16.1162	0.0565	7.37E-11
CAGCCCAG	-106	150	5.0782	0.2253	5.0213	8.7204	0.0351	2.02E-09
ACGGAACG	-102	124	5.4860	0.2231	5.9362	8.1984	0.0152	3.88E-07
CCAAGCCC	-67	168	4.7400	0.2231	6.5332	12.3755	0.0306	5.67E-09
AACTGGGC	-96	150	4.9282	0.2229	6.5126	9.6266	0.0223	4.14E-08
GGCCGAGA	-51	152	4.6665	0.2205	5.8706	9.2691	0.0275	1.33E-08
ACCGGCCC	-76	132	6.0715	0.2203	6.9420	12.6067	0.0216	6.03E-08
CGGGTCCC	-55	135	6.0087	0.2202	5.9178	8.5235	0.0153	4.44E-07
CGCGTCTC	-51	117	6.8484	0.2196	6.3136	10.5796	0.0201	9.55E-08
CAACGGCC	-75	155	6.8391	0.2188	6.1070	14.0135	0.0244	3.14E-08
ATATGGGC	-98	172	4.4453	0.2175	5.1265	8.5763	0.0416	1.11E-09
ATTTGGGC	-78	182	4.1724	0.2175	6.6839	10.0892	0.0428	9.22E-10
CGGGCCGA	-99	122	6.6981	0.2167	5.5616	10.0088	0.0196	1.33E-07

CGCGCGAG	-90	179	3.9426	0.2155	5.0871	7.3690	0.0411	1.41E-09
CCCCCGCG	-58	209	4.1599	0.2092	5.1061	10.3927	0.0394	2.91E-09
CGGACGGC	-79	183	4.0237	0.2080	5.4681	9.3334	0.0468	1.02E-09
CACGTCAC	-73	167	6.0163	0.2076	6.5249	15.5496	0.0335	9.31E-09
GGTCCAGA	-84	118	5.8747	0.2057	5.2253	9.1022	0.0176	4.91E-07
ACCCGCGC	-98	112	5.1684	0.2033	6.5941	8.8449	0.0223	1.50E-07
GGACGGCC	-83	166	4.2146	0.2024	5.1774	9.4893	0.0342	1.20E-08
ACGCGTCC	-120	143	4.7354	0.2002	5.0791	8.4331	0.0187	4.91E-07
CGACCCGC	-80	118	5.6093	0.2002	5.4821	8.4288	0.0157	1.30E-06
GGCCCGCA	-66	118	5.4383	0.1989	5.1581	6.8784	0.0163	1.13E-06
GCGCGCGA	-85	160	3.9964	0.1985	5.2060	7.7946	0.0486	1.65E-09
CGCGCGAC	-84	166	3.6879	0.1975	5.0412	6.8303	0.0310	3.11E-08
CACGTCTC	-61	111	4.4596	0.1946	5.2191	7.2588	0.0219	2.94E-07
AGCCCATA	-102	172	4.1780	0.1945	5.6224	9.5426	0.0321	3.14E-08
GCCGGCCC	-73	166	3.5702	0.1944	5.2293	7.5917	0.0563	8.56E-10
GCCGGGCC	-68	113	5.5059	0.1936	10.0731	17.2617	0.0435	5.01E-09
CAAGCCCA	-62	151	3.7789	0.1936	5.6502	8.5449	0.0392	9.76E-09
AGCCGTTG	-95	121	5.6587	0.1933	6.5191	11.3645	0.0252	1.45E-07
CCACGGCC	-60	177	4.0747	0.1927	5.0063	8.7478	0.0437	5.17E-09
AAGCCCAG	-93	151	3.7923	0.1915	5.6973	7.9250	0.0256	1.49E-07
CCCGGGCC	-93	139	5.0998	0.1914	5.6570	9.2225	0.0224	3.28E-07
AGCCCAAG	-134	162	3.5756	0.1877	6.2081	9.0692	0.0349	3.15E-08
CCCACACG	-59	108	5.6961	0.1874	8.6785	12.7633	0.0312	6.33E-08
CCACTGAC	-164	139	4.5011	0.1840	5.3921	8.7568	0.0289	1.28E-07
GCCCATAC	-119	163	4.8295	0.1817	5.1326	10.3301	0.0208	9.49E-07
CCCATCCA	-73	176	3.2628	0.1802	5.0576	7.4811	0.0517	4.80E-09
GGACCCAC	-95	192	4.7021	0.1801	5.1115	11.0644	0.0590	2.03E-09
CGTGTGGC	-112	142	4.4367	0.1758	5.7969	10.0253	0.0257	4.63E-07
CGCACCGC	-115	141	3.9780	0.1749	5.6754	8.1337	0.0381	5.02E-08
ACATGGGC	-74	155	3.3477	0.1734	5.1265	7.5920	0.0393	4.70E-08
GCCCATGA	-59	133	3.7373	0.1733	6.4061	9.7749	0.0374	6.32E-08
GGGCCTGA	-73	130	5.0209	0.1706	5.5490	12.6216	0.0216	1.73E-06
CGTGGACC	-149	136	3.6053	0.1700	5.3207	6.8825	0.0237	1.11E-06
ATCCCCCA	-67	135	4.3502	0.1680	5.4670	9.2869	0.0290	4.21E-07
GCCCCACA	-61	117	4.3561	0.1653	7.0806	10.2362	0.0441	4.56E-08
ACGTCACC	-82	103	6.4074	0.1651	6.3953	13.1264	0.0231	1.81E-06
GCAGCCCA	-101	153	3.5247	0.1645	5.3623	7.8824	0.0346	2.04E-07
CGGACGGA	-55	130	4.5295	0.1642	5.4709	8.5572	0.0272	8.08E-07
CACACACC	-54	172	3.1969	0.1620	5.0191	8.1854	0.0437	6.47E-08
AGTGACAC	-144	144	4.1162	0.1611	5.2379	9.2326	0.0269	1.08E-06
AGAGTGGG	-66	114	4.0308	0.1550	5.1927	6.6113	0.0227	4.23E-06
CCCACTCC	-51	110	4.9873	0.1530	6.7046	12.1407	0.0407	2.13E-07
AAACGGCC	-102	97	5.3827	0.1518	5.5575	9.8161	0.0232	4.94E-06
GGTCCCAC	-98	113	4.1275	0.1495	9.4937	12.3441	0.0657	1.66E-08
GGGACCCA	-94	146	4.5781	0.1485	5.1349	10.7907	0.0616	2.75E-08
CCACCAAC	-53	144	3.4899	0.1469	5.4541	9.7473	0.0530	7.97E-08

ATCTCGGC	-64	107	4.0130	0.1431	5.1009	7.4234	0.0218	1.31E-05
CAAGTGGG	-116	106	3.9280	0.1367	5.0265	7.3285	0.0314	3.63E-06
CACGCCTC	-50	104	3.4124	0.1357	5.1684	6.7337	0.0259	1.04E-05
ACGGCCCG	-97	93	3.9838	0.1351	5.6996	7.9938	0.0298	5.43E-06
CTCCCCCA	-50	145	3.5765	0.1337	5.1475	9.7080	0.0568	1.91E-07
CGATGGGC	-102	91	4.8385	0.1323	6.5319	10.6542	0.0269	1.16E-05
GCTTTCCC	-59	79	4.8187	0.1308	5.3351	6.7249	0.0232	2.68E-05
GGTGGGAC	-64	115	3.9130	0.1294	5.3654	8.3424	0.0365	3.22E-06
GGGTCCCA	-93	124	3.7617	0.1284	5.2283	8.5519	0.0590	2.64E-07
GGCGTGGA	-71	110	3.3140	0.1228	5.1060	7.3903	0.0369	5.73E-06
CATCCCCC	-68	71	4.7027	0.1195	5.4558	8.8084	0.0272	3.40E-05
CTCTCCGC	-55	88	3.5184	0.1128	5.2446	7.6858	0.0388	1.18E-05
CATCCACC	-58	106	3.1140	0.1118	5.8627	8.6971	0.0459	5.75E-06
CCGAGCCG	-71	88	3.7499	0.1112	5.6228	8.1397	0.0437	7.84E-06
AGCTGAGC	-51	54	5.1949	0.1058	7.0409	10.1312	0.0301	7.69E-05
CCTCGCCC	-51	78	4.0596	0.1057	5.5381	9.0852	0.0419	1.70E-05
